# Supplementary material for: Quantitative risk assessment and interventional recommendations for preventing canine distemper virus infection in captive tigers at selected wildlife stations in Thailand
Source: PLoS One. 2025 Apr 17;20(4):e0320657. doi: 10.1371/journal.pone.0320657 (PMC12005548; doi:10.1371/journal.pone.0320657)
Supplement: S1 File — S1 Table 1. Captive tiger population number in Department of National Parks wildlife stations in 2021. S2 Figs 3A. Scenario trees depicting the biological pathways of CDV introductions into captive wildlife stations through infected tigers with and without interventions. (3A) Scenario tree without any interventions. (3.1A) With the intervention of isolation and treatment of sick tigers. (3.2.1A) With CDV testing at the place of origin. (3.2.2A) With CDV testing at the destined wildlife station. (3.2.3A) With CDV testing in both original and destined stations. (3.3A) With CDV vaccinations. (3.4A) With all combined interventions. (3.5A) With CDV testing in both stations and vaccinations. (3.6A) With CDV testing in both stations and isolation and treatment of sick tigers. (3.7A) With isolation and treatment of sick tigers and vaccinations. S3 Figs 3B. Scenario trees depicting the biological pathways of CDV introductions into captive wildlife stations through other infected wild animals with and without interventions. (3B) Scenario tree without any interventions. (3.1B) With isolation and treatment of sick wild animals. (3.2.1B) With CDV testing at the place of origin. (3.2.2B) With CDV testing at the destined wildlife station. (3.2.3B) With CDV testing in both original and destined stations. (3.3B) With CDV vaccinations. (3.4B) With all interventions. (3.5B) With CDV testing in both stations and vaccinations. (3.6B) With CDV testing in both stations and isolation and treatment of sick wild animals. (3.7B) With isolation and treatment of sick wild animals and vaccinations. S4 Figs 3C. Scenario tree depicting the biological pathway of CDV introductions into captive wildlife stations through dog or cat reservoirs with and without interventions. (3C) Scenario tree without interventions. (3.1C) With intervention of not allowing dogs or cats enter areas near tiger cages. S5 Figs 3D-3F. Scenario trees depicting the biological pathways of CDV introductions into captive wildlif [file pone.0320657.s001.pdf]

Supporting information

S1 Table 1

Order	Wildlife Rescue Centers and Wildlife Breeding Stations	August 2021	
		Number of original tigers	Number of confiscated tigers
1.	Wildlife Rescue Center 1	-	7
2.	Wildlife Rescue Center 2	-	21
3.	Wildlife Breeding Station 1	3	24
4.	Wildlife Breeding Station 2	-	2
5.	Wildlife Breeding Station 3	-	1
6.	Wildlife Breeding Station 4	-	14
7.	Wildlife Breeding Station 5	11	-
8.	Wildlife Breeding Station 6	15	5
9.	Wildlife Breeding Station 7	12	2
10.	Wildlife Breeding Station 8	1	1
	Total	42	77

Supporting information

Figs S2

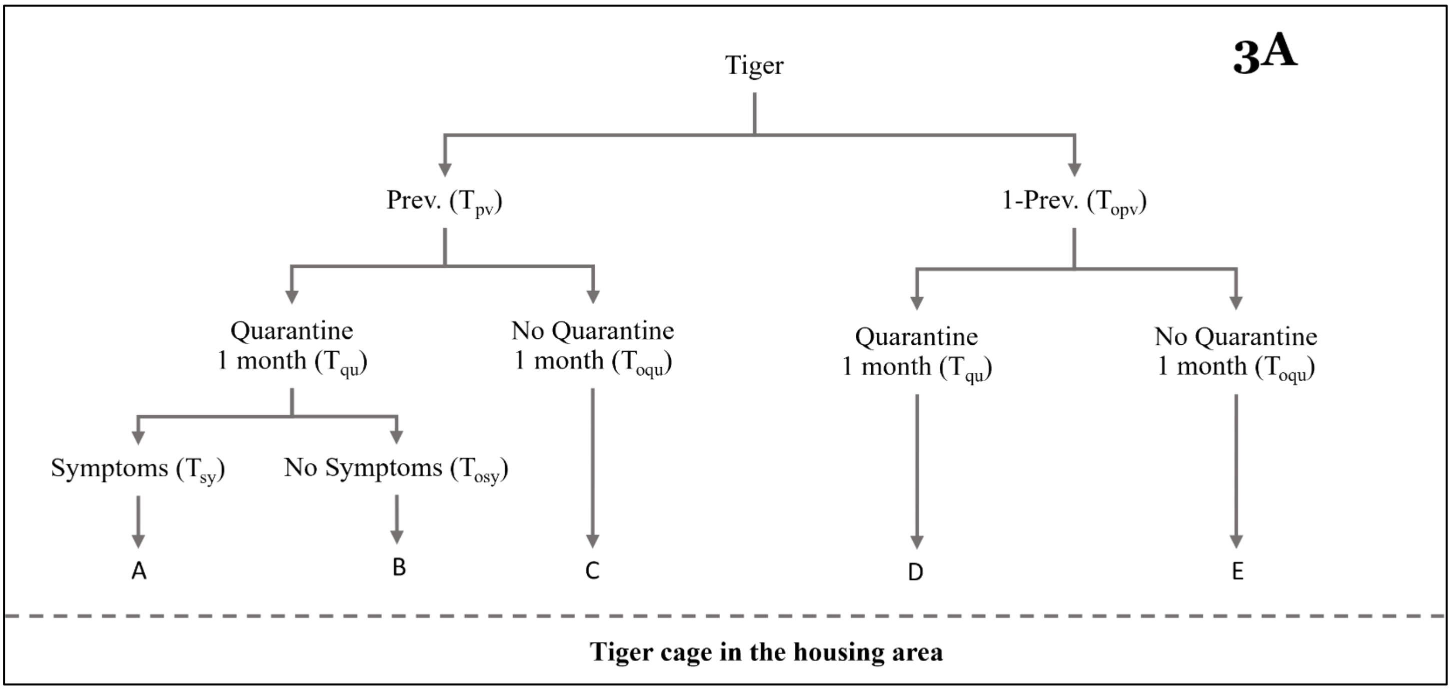

$P_{Tiger} = (A + B + C)/(A + B + C + D + E)$ (equation for Fig. 3A)

sub-equations of P_{Tiger}	$A = T_{pv} * T_{qu} * T_{sy}$
	$B = T_{pv} * T_{qu} * T_{osy}$
	$C = T_{pv} * T_{oqu}$
	$D = T_{opv} * T_{qu}$
	$E = T_{opv} * T_{oqu}$

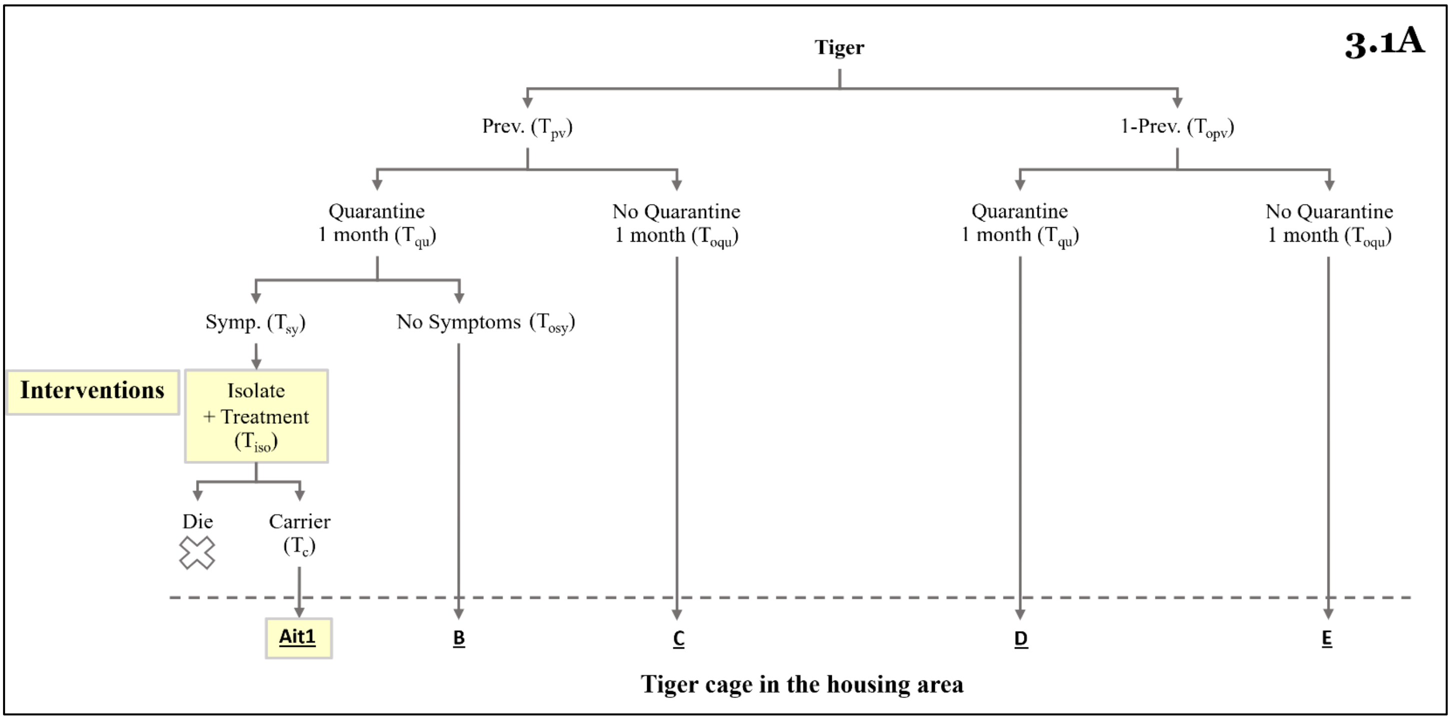

$P_{TIso} \quad = \quad (Ait1 + B + C)/(Ait1 + B + C + D + E)$ (equation for Fig. 3.1A)

sub-equations of P_{TIso}	$A_{it1} = T_{pv} * T_{qu} * T_{sy} * T_{iso} * T_c$
	$B = T_{pv} * T_{qu} * T_{osy}$
	$C = T_{pv} * T_{oqu}$
	$D = T_{opv} * T_{qu}$
	$E = T_{opv} * T_{oqu}$

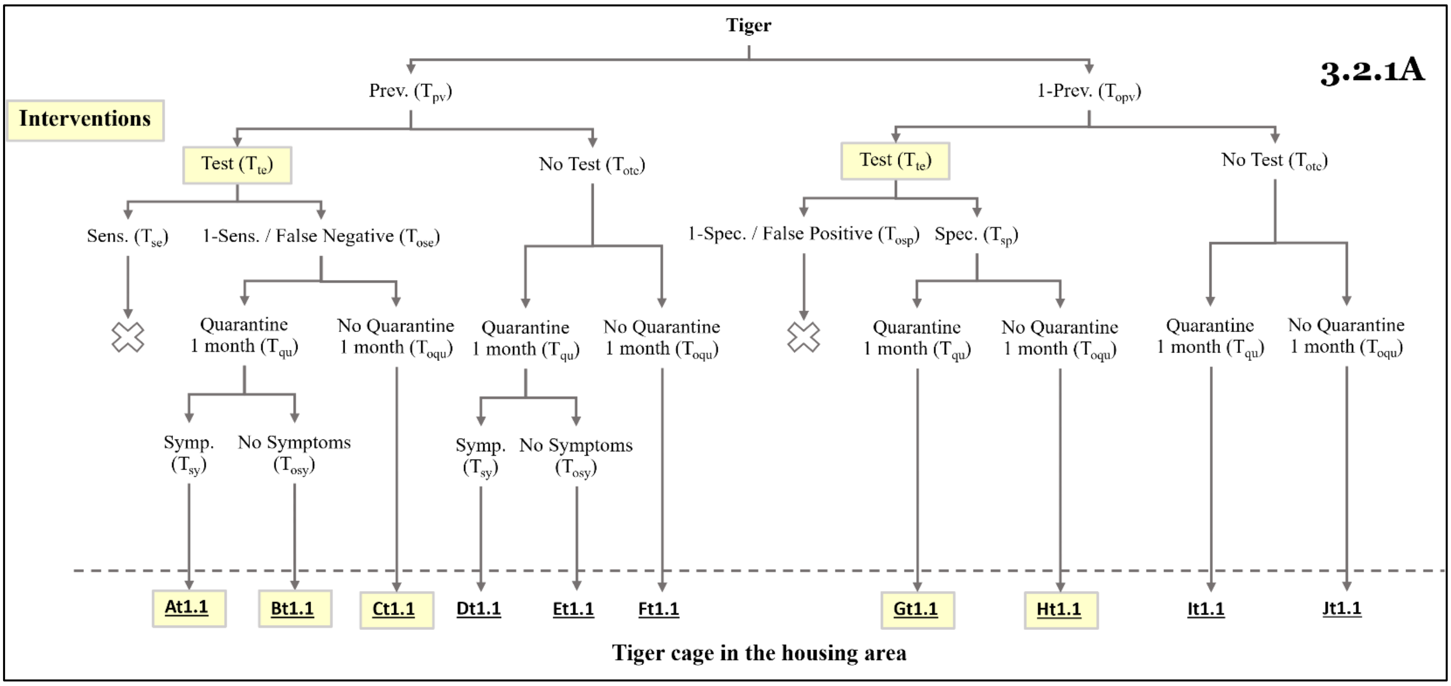

P_{TTori} = (At1.1 + Bt1.1 + Ct1.1 + Dt1.1 + Et1.1 + Ft1.1)/(At1.1 + Bt1.1 + Ct1.1 + Dt1.1 + Et1.1 + Ft1.1 + Gt1.1 + Ht1.1 + It1.1 + Jt1.1) (equation for Fig. 3.2.1A)

sub-equations of P_{TTori}	$A_{t1.1} = T_{pv} * T_{te} * T_{ose} * T_{qu} * T_{sy}$
	$B_{t1.1} = T_{pv} * T_{te} * T_{ose} * T_{qu} * T_{osy}$
	$C_{t1.1} = T_{pv} * T_{te} * T_{ose} * T_{oqu}$
	$D_{t1.1} = T_{pv} * T_{ote} * T_{qu} * T_{sy}$
	$E_{t1.1} = T_{pv} * T_{ote} * T_{qu} * T_{osy}$
	$F_{t1.1} = T_{pv} * T_{ote} * T_{oqu}$
	$G_{t1.1} = T_{opv} * T_{te} * T_{sp} * T_{qu}$
	$H_{t1.1} = T_{opv} * T_{te} * T_{sp} * T_{oqu}$
	$I_{t1.1} = T_{opv} * T_{ote} * T_{qu}$
	$J_{t1.1} = T_{opv} * T_{ote} * T_{oqu}$

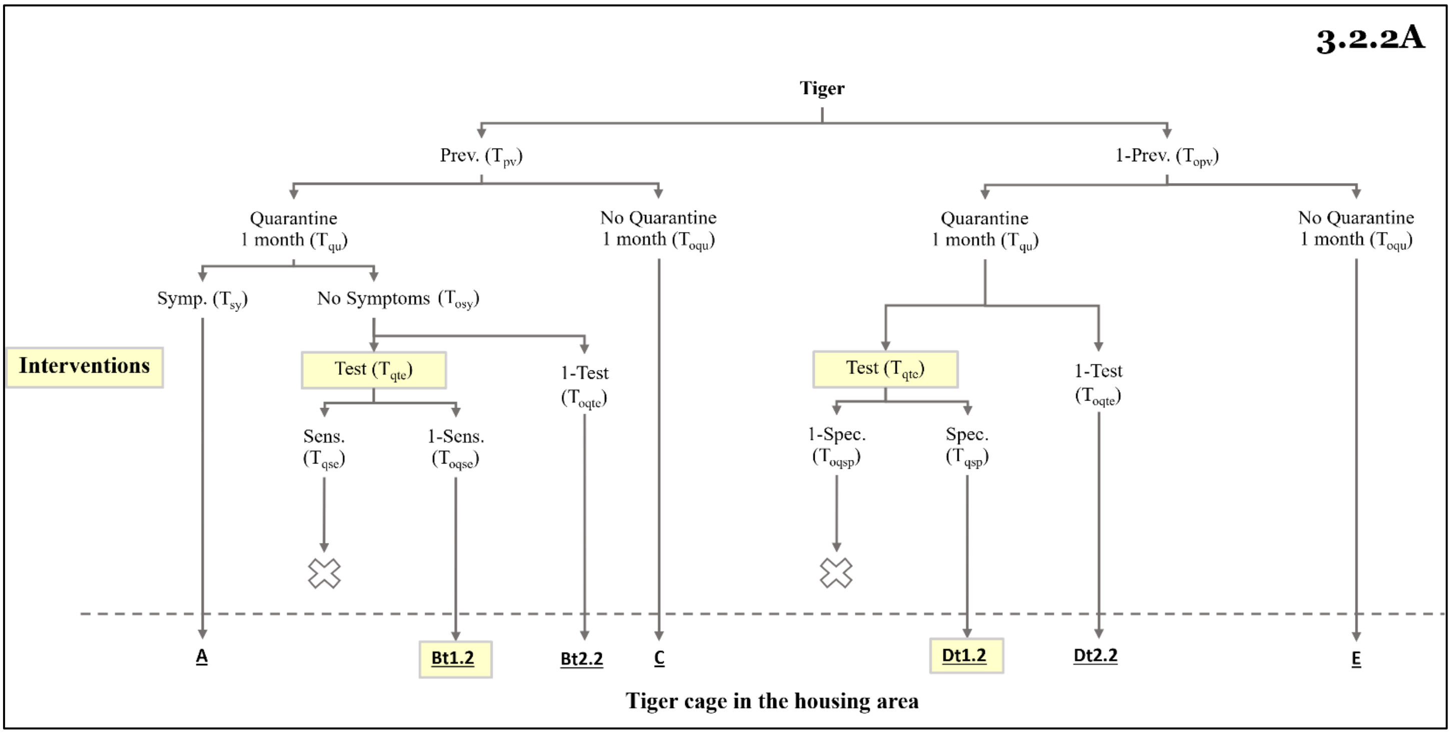

$P_{TTsta} = (A + Bt1.2 + Bt2.2 + C)/(A + Bt1.2 + Bt2.2 + C + Dt1.2 + Dt2.2 + E)$ (equation for Fig. 3.2.2A)

sub-equations of P_{TTsta}	$A = T_{pv} * T_{qu} * T_{sy}$
	$B_{t1.2} = T_{pv} * T_{qu} * T_{osy} * T_{qte} * T_{oqse}$
	$B_{t2.2} = T_{pv} * T_{qu} * T_{osy} * T_{oqte}$
	$C = T_{pv} * T_{oqu}$
	$D_{t1.2} = T_{opv} * T_{qu} * T_{qte} * T_{qsp}$
	$D_{t2.2} = T_{opv} * T_{qu} * T_{oqte}$
	$E = T_{opv} * T_{oqu}$

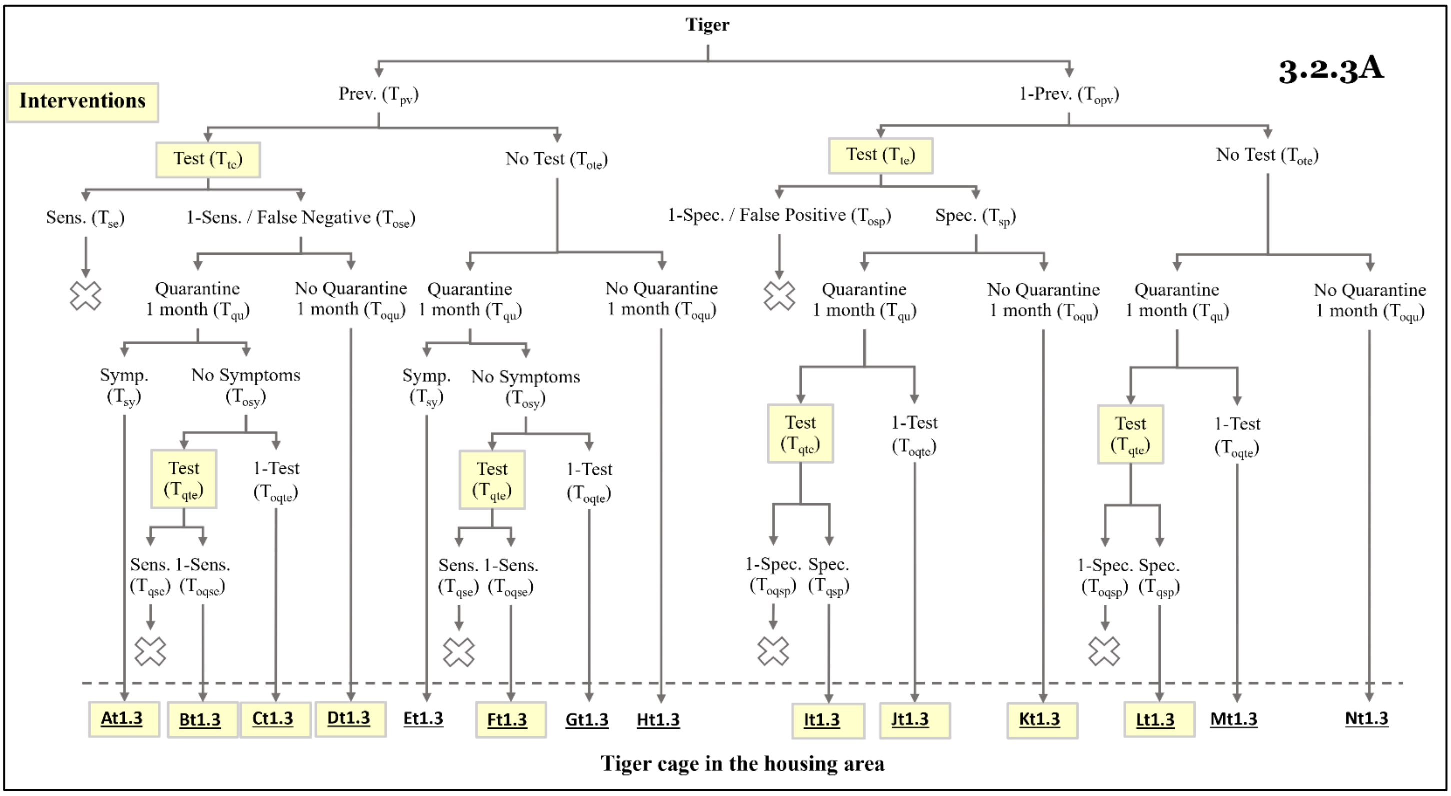

P_{TTos} = (At1.3 + Bt1.3 + Ct1.3 + Dt1.3 + Et1.3 + Ft1.3 + Gt1.3 + Ht1.3)/ (At1.3 + Bt1.3 + Ct1.3 + Dt1.3 + Et1.3 + Ft1.3 + Gt1.3 + Ht1.3 + It1.3 + Jt1.3 + Kt1.3 + Lt1.3 + Mt1.3 + Nt1.3) (equation for Fig. 3.2.3A)

sub-equations of P_{TTos}	$A_{t1.3} = T_{pv} * T_{te} * T_{ose} * T_{qu} * T_{sy}$
	$B_{t1.3} = T_{pv} * T_{te} * T_{ose} * T_{qu} * T_{osy} * T_{qte} * T_{oqse}$
	$C_{t1.3} = T_{pv} * T_{te} * T_{ose} * T_{qu} * T_{osy} * T_{oqte}$
	$D_{t1.3} = T_{pv} * T_{te} * T_{ose} * T_{oqu}$
	$E_{t1.3} = T_{pv} * T_{ote} * T_{qu} * T_{sy}$
	$F_{t1.3} = T_{pv} * T_{ote} * T_{qu} * T_{osy} * T_{qte} * T_{oqse}$
	$G_{t1.3} = T_{pv} * T_{ote} * T_{qu} * T_{osy} * T_{oqte}$
	$H_{t1.3} = T_{pv} * T_{ote} * T_{oqu}$
	$I_{t1.3} = T_{opv} * T_{te} * T_{sp} * T_{qu} * T_{qte} * T_{qsp}$
	$J_{t1.3} = T_{opv} * T_{te} * T_{sp} * T_{qu} * T_{oqte}$
	$K_{t1.3} = T_{opv} * T_{te} * T_{sp} * T_{oqu}$
	$L_{t1.3} = T_{opv} * T_{ote} * T_{qu} * T_{qte} * T_{qsp}$
	$M_{t1.3} = T_{opv} * T_{ote} * T_{qu} * T_{oqte}$
	$N_{t1.3} = T_{opv} * T_{ote} * T_{oqu}$

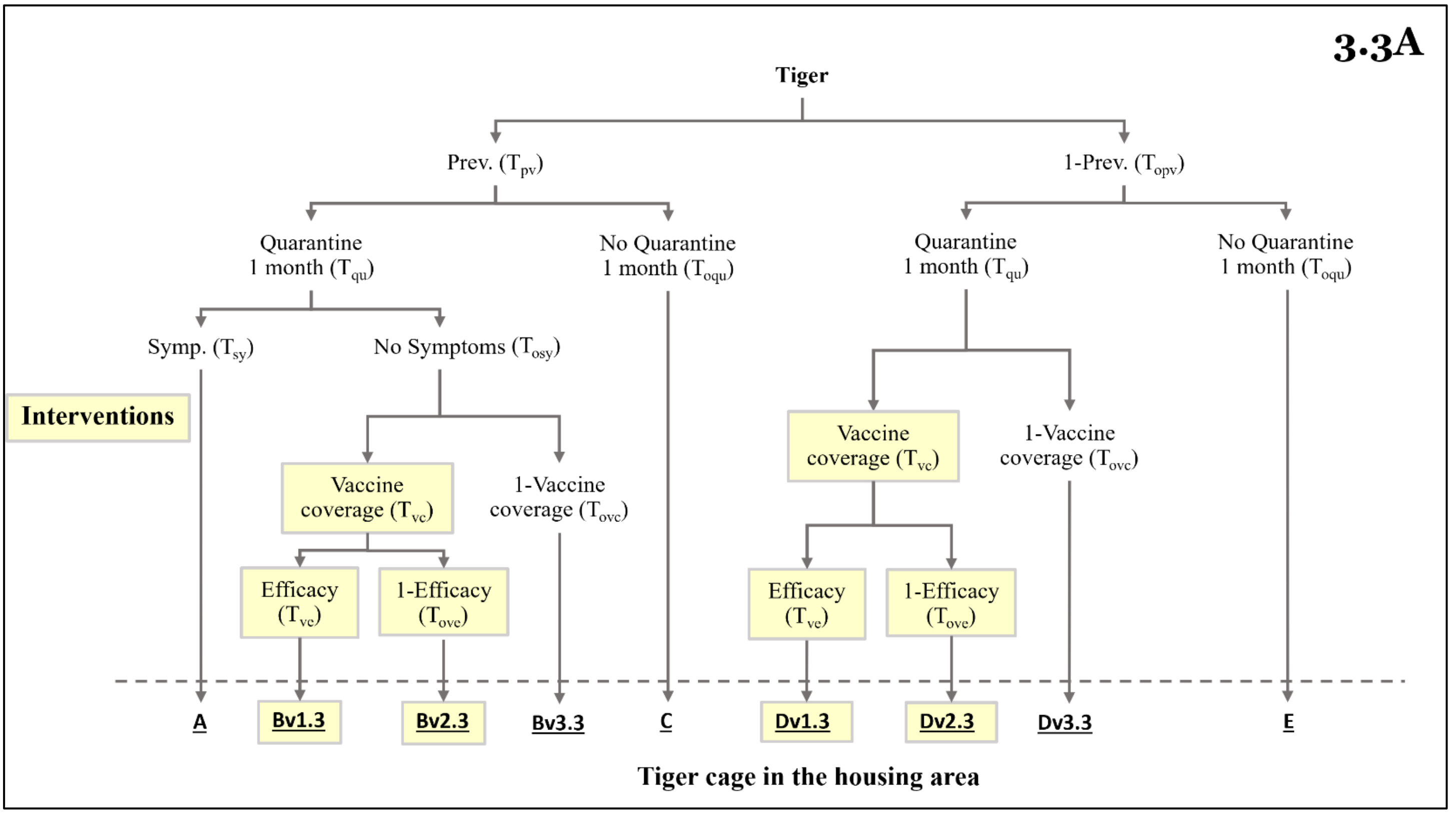

$P_{TVac} = (A + Bv2.3 + Bv3.3 + C)/(A + Bv1.3 + Bv2.3 + Bv3.3 + C + Dv1.3 + Dv2.3 + Dv3.3 + E)$ (equation for Fig. 3.3A)

sub-equations of P_{TVac}	$A = T_{pv} * T_{qu} * T_{sy}$
	$B_{v1.3} = T_{pv} * T_{qu} * T_{osy} * T_{vc} * T_{ve}$
	$B_{v2.3} = T_{pv} * T_{qu} * T_{osy} * T_{vc} * (1- T_{ve})$
	$B_{v3.3} = T_{pv} * T_{qu} * T_{osy} * (1- T_{vc})$
	$C = T_{pv} * T_{oqu}$
	$D_{v1.3} = T_{opv} * T_{qu} * T_{vc} * T_{ve}$
	$D_{v2.3} = T_{opv} * T_{qu} * T_{vc} * (1- T_{ve})$
	$D_{v3.3} = T_{opv} * T_{qu} * (1- T_{vc})$
	$E = T_{opv} * T_{oqu}$

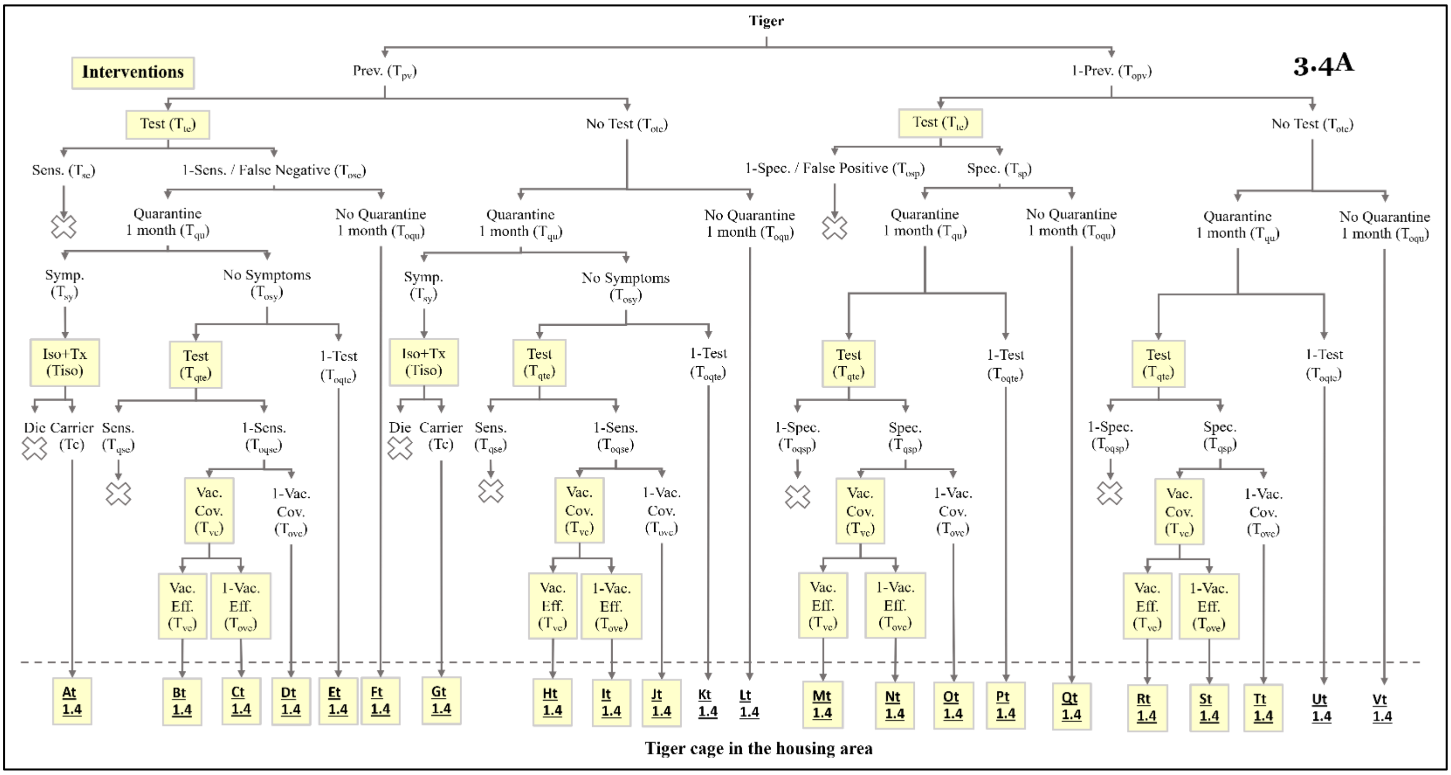

$P_{TTos+Iso+V}$ = (At1.4 + Ct1.4 + Dt1.4 + Et1.4 + Ft1.4 + Gt1.4 + It1.4 + Jt1.4 + Kt1.4 + Lt1.4)/ (At1.4 + Bt1.4 + Ct1.4 + Dt1.4 + Et1.4 + Ft1.4 + Gt1.4 + Ht1.4 + It1.4 + Jt1.4 + Kt1.4 + Lt1.4 + Mt1.4 + Nt1.4 + Ot1.4 + Pt1.4 + Qt1.4 + Rt1.4 + St1.4 + Tt1.4 + Ut1.4 + Vt1.4) (equation for Fig. 3.4A)

sub-equations of $P_{TTos+Iso+V}$	$A_{t1.4}$ = T_{pv} * T_{te} * T_{ose} * T_{qu} * T_{sy} * T_{iso} * T_c
	$B_{t1.4}$ = T_{pv} * T_{te} * T_{ose} * T_{qu} * T_{osy} * T_{qte} * T_{oqse} * T_{vc} * T_{ve}
	$C_{t1.4}$ = T_{pv} * T_{te} * T_{ose} * T_{qu} * T_{osy} * T_{qte} * T_{oqse} * T_{vc} * 1-T_{ve}
	$D_{t1.4}$ = T_{pv} * T_{te} * T_{ose} * T_{qu} * T_{osy} * T_{qte} * T_{oqse} * 1-T_{vc}
	$E_{t1.4}$ = T_{pv} * T_{te} * T_{ose} * T_{qu} * T_{osy} * T_{oqte}
	$F_{t1.4}$ = T_{pv} * T_{te} * T_{ose} * T_{oqu}
	$G_{t1.4}$ = T_{pv} * T_{ote} * T_{qu} * T_{sy} * T_{iso} * T_c
	$H_{t1.4}$ = T_{pv} * T_{ote} * T_{qu} * T_{osy} * T_{qte} * T_{oqse} * T_{vc} * T_{ve}
	$I_{t1.4}$ = T_{pv} * T_{ote} * T_{qu} * T_{osy} * T_{qte} * T_{oqse} * T_{vc} * 1-T_{ve}
	$J_{t1.4}$ = T_{pv} * T_{ote} * T_{qu} * T_{osy} * T_{qte} * T_{oqse} * 1-T_{vc}
	$K_{t1.4}$ = T_{pv} * T_{ote} * T_{qu} * T_{osy} * T_{oqte}
	$L_{t1.4}$ = T_{pv} * T_{ote} * T_{oqu}
	$M_{t1.4}$ = T_{opv} * T_{te} * T_{sp} * T_{qu} * T_{qte} * T_{qsp} * T_{vc} * T_{ve}

sub-equations of $P_{TTos+Iso+V}$	$N_{t1.4}$ = T_{opv} * T_{te} * T_{sp} * T_{qu} * T_{qte} * T_{qsp} * T_{vc} * 1-T_{ve}
	$O_{t1.4}$ = T_{opv} * T_{te} * T_{sp} * T_{qu} * T_{qte} * T_{qsp} * 1-T_{vc}
	$P_{t1.4}$ = T_{opv} * T_{te} * T_{sp} * T_{qu} * T_{oqte}
	$Q_{t1.4}$ = T_{opv} * T_{te} * T_{sp} * T_{oqu}
	$R_{t1.4}$ = T_{opv} * T_{ote} * T_{qu} * T_{qte} * T_{qsp} * T_{vc} * T_{ve}
	$S_{t1.4}$ = T_{opv} * T_{ote} * T_{qu} * T_{qte} * T_{qsp} * T_{vc} * 1-T_{ve}
	$T_{t1.4}$ = T_{opv} * T_{ote} * T_{qu} * T_{qte} * T_{qsp} * 1-T_{vc}
	$U_{t1.4}$ = T_{opv} * T_{ote} * T_{qu} * T_{oqte}
	$V_{t1.4}$ = T_{opv} * T_{ote} * T_{oqu}

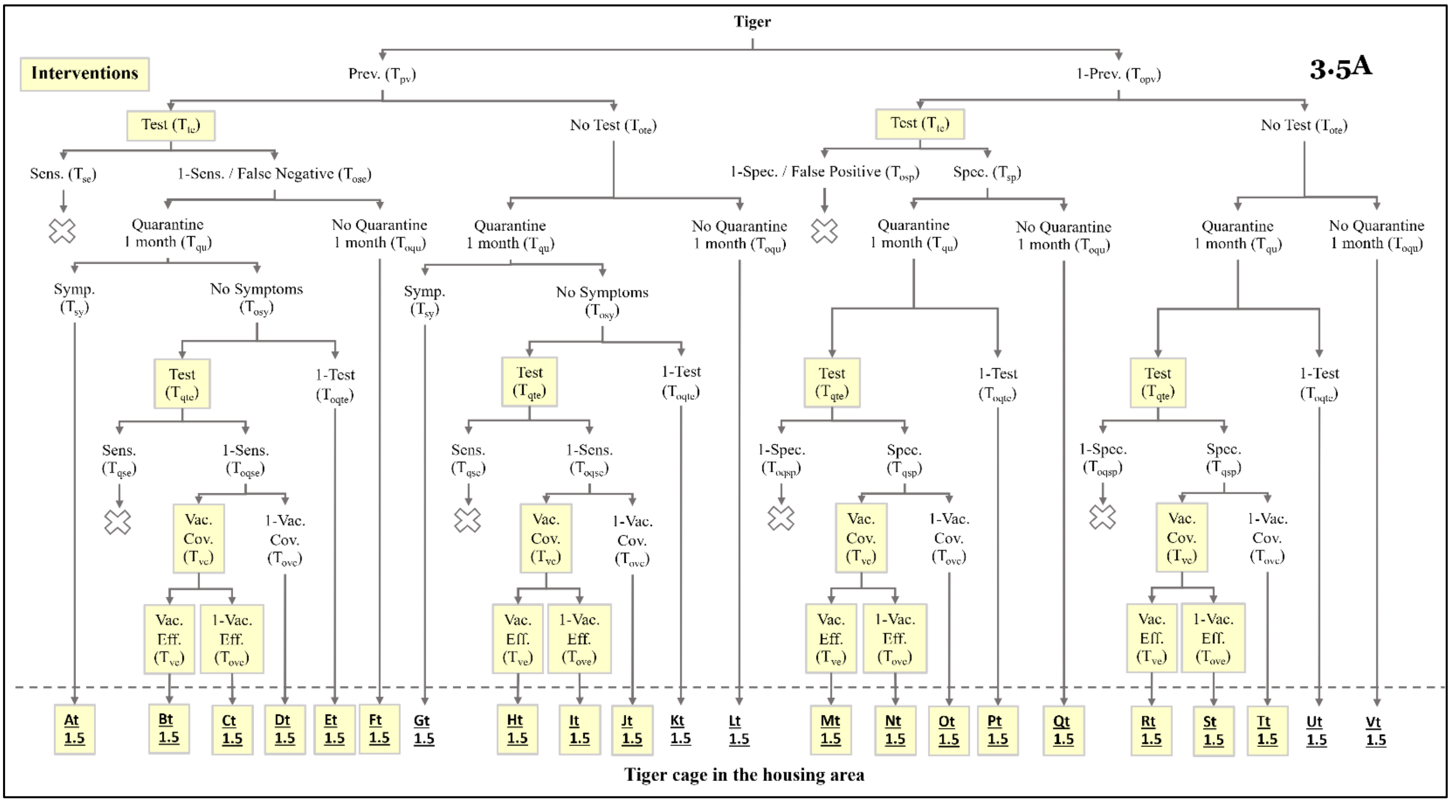

$$P_{TTos+V} = (At1.5 + Ct1.5 + Dt1.5 + Et1.5 + Ft1.5 + Gt1.5 + It1.5 + Jt1.5 + Kt1.5 + Lt1.5) / (At1.5 + Bt1.5 + Ct1.5 + Dt1.5 + Et1.5 + Ft1.5 + Gt1.5 + Ht1.5 + It1.5 + Jt1.5 + Kt1.5 + Lt1.5 + Mt1.5 + Nt1.5 + Ot1.5 + Pt1.5 + Qt1.5 + Rt1.5 + St1.5 + Tt1.5 + Ut1.5 + Vt1.5)$$

(equation for Fig. 3.5A)

sub-equations of P_{TTos+V}	$A_{t1.5} = T_{pv} * T_{te} * T_{ose} * T_{qu} * T_{sy}$
	$B_{t1.5} = T_{pv} * T_{te} * T_{ose} * T_{qu} * T_{osy} * T_{qte} * T_{oqse} * T_{vc} * T_{ve}$
	$C_{t1.5} = T_{pv} * T_{te} * T_{ose} * T_{qu} * T_{osy} * T_{qte} * T_{oqse} * T_{vc} * 1\text{-}T_{ve}$
	$D_{t1.5} = T_{pv} * T_{te} * T_{ose} * T_{qu} * T_{osy} * T_{qte} * T_{oqse} * 1\text{-}T_{vc}$
	$E_{t1.5} = T_{pv} * T_{te} * T_{ose} * T_{qu} * T_{osy} * T_{oqte}$
	$F_{t1.5} = T_{pv} * T_{te} * T_{ose} * T_{oqu}$
	$G_{t1.5} = T_{pv} * T_{ote} * T_{qu} * T_{sy}$
	$H_{t1.5} = T_{pv} * T_{ote} * T_{qu} * T_{osy} * T_{qte} * T_{oqse} * T_{vc} * T_{ve}$
	$I_{t1.5} = T_{pv} * T_{ote} * T_{qu} * T_{osy} * T_{qte} * T_{oqse} * T_{vc} * 1\text{-}T_{ve}$
	$J_{t1.5} = T_{pv} * T_{ote} * T_{qu} * T_{osy} * T_{qte} * T_{oqse} * 1\text{-}T_{vc}$
	$K_{t1.5} = T_{pv} * T_{ote} * T_{qu} * T_{osy} * T_{oqte}$
	$L_{t1.5} = T_{pv} * T_{ote} * T_{oqu}$
	$M_{t1.5} = T_{opv} * T_{te} * T_{sp} * T_{qu} * T_{qte} * T_{qsp} * T_{vc} * T_{ve}$

sub-equations of P_{TTos+V}	$N_{t1.5}$ = T_{opv} * T_{te} * T_{sp} * T_{qu} * T_{qte} * T_{qsp} * T_{vc} * 1-T_{ve}
	$O_{t1.5}$ = T_{opv} * T_{te} * T_{sp} * T_{qu} * T_{qte} * T_{qsp} * 1-T_{vc}
	$P_{t1.5}$ = T_{opv} * T_{te} * T_{sp} * T_{qu} * T_{oqte}
	$Q_{t1.5}$ = T_{opv} * T_{te} * T_{sp} * T_{oqu}
	$R_{t1.5}$ = T_{opv} * T_{ote} * T_{qu} * T_{qte} * T_{qsp} * T_{vc} * T_{ve}
	$S_{t1.5}$ = T_{opv} * T_{ote} * T_{qu} * T_{qte} * T_{qsp} * T_{vc} * 1-T_{ve}
	$T_{t1.5}$ = T_{opv} * T_{ote} * T_{qu} * T_{qte} * T_{qsp} * 1-T_{vc}
	$U_{t1.5}$ = T_{opv} * T_{ote} * T_{qu} * T_{oqte}
	$V_{t1.5}$ = T_{opv} * T_{ote} * T_{oqu}

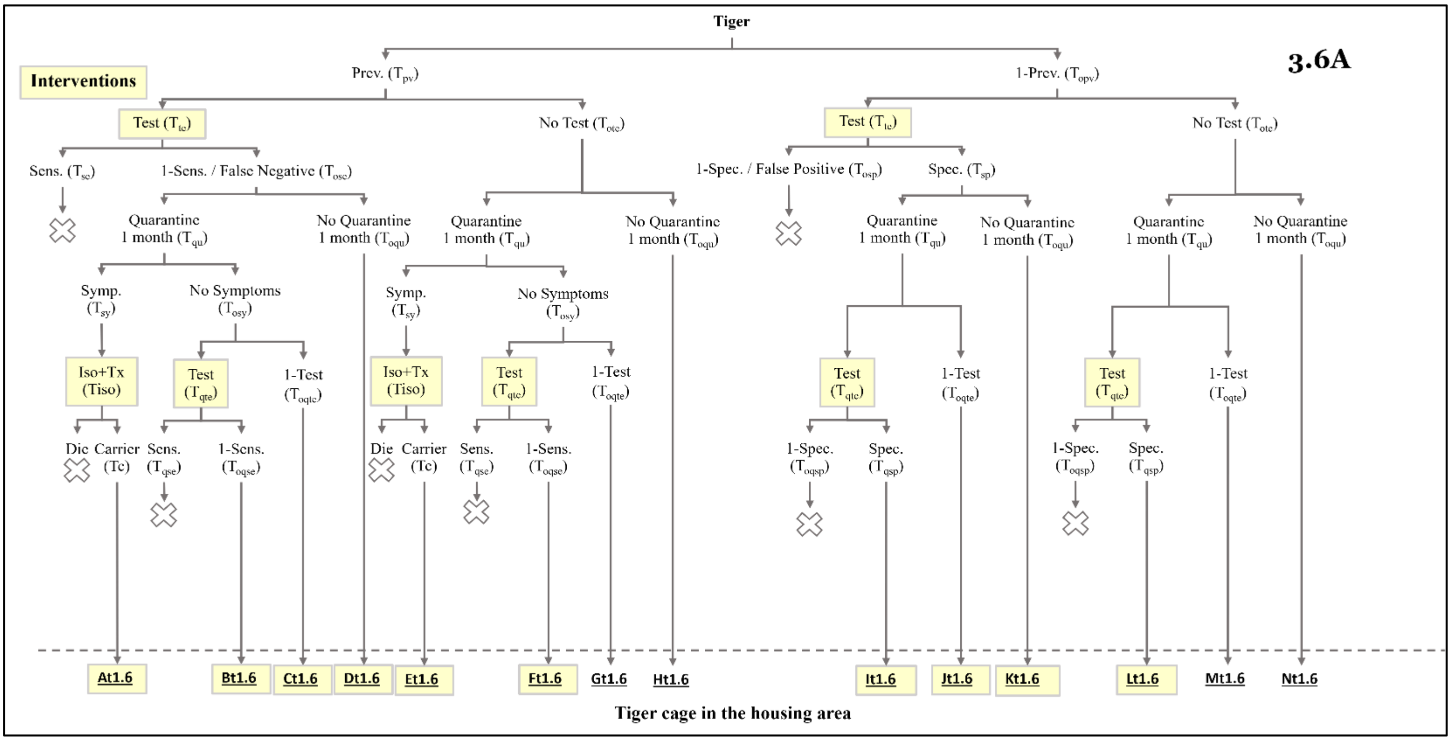

$$P_{TTos+Iso} = (At1.6 + Bt1.6 + Ct1.6 + Dt1.6 + Et1.6 + Ft1.6 + Gt1.6 + Ht1.6) / (At1.6 + Bt1.6 + Ct1.6 + Dt1.6 + Et1.6 + Ft1.6 + Gt1.6 + Ht1.6 + It1.6 + Jt1.6 + Kt1.6 + Lt1.6 + Mt1.6 + Nt1.6)$$

(equation for Fig. 3.6A)

sub-equations of $P_{TTos+Iso}$	$A_{t1.6} = T_{pv} * T_{te} * T_{ose} * T_{qu} * T_{sy} * T_{iso} * T_c$
	$B_{t1.6} = T_{pv} * T_{te} * T_{ose} * T_{qu} * T_{osy} * T_{qte} * T_{oqse}$
	$C_{t1.6} = T_{pv} * T_{te} * T_{ose} * T_{qu} * T_{osy} * T_{oqte}$
	$D_{t1.6} = T_{pv} * T_{te} * T_{ose} * T_{oqu}$
	$E_{t1.6} = T_{pv} * T_{ote} * T_{qu} * T_{sy} * T_{iso} * T_c$
	$F_{t1.6} = T_{pv} * T_{ote} * T_{qu} * T_{osy} * T_{qte} * T_{oqse}$
	$G_{t1.6} = T_{pv} * T_{ote} * T_{qu} * T_{osy} * T_{oqte}$
	$H_{t1.6} = T_{pv} * T_{ote} * T_{oqu}$
	$I_{t1.6} = T_{opv} * T_{te} * T_{sp} * T_{qu} * T_{qte} * T_{qsp}$
	$J_{t1.6} = T_{opv} * T_{te} * T_{sp} * T_{qu} * T_{oqte}$
	$K_{t1.6} = T_{opv} * T_{te} * T_{sp} * T_{oqu}$
	$L_{t1.6} = T_{opv} * T_{ote} * T_{qu} * T_{qte} * T_{qsp}$
	$M_{t1.6} = T_{opv} * T_{ote} * T_{qu} * T_{oqte}$
	$N_{t1.6} = T_{opv} * T_{ote} * T_{oqu}$

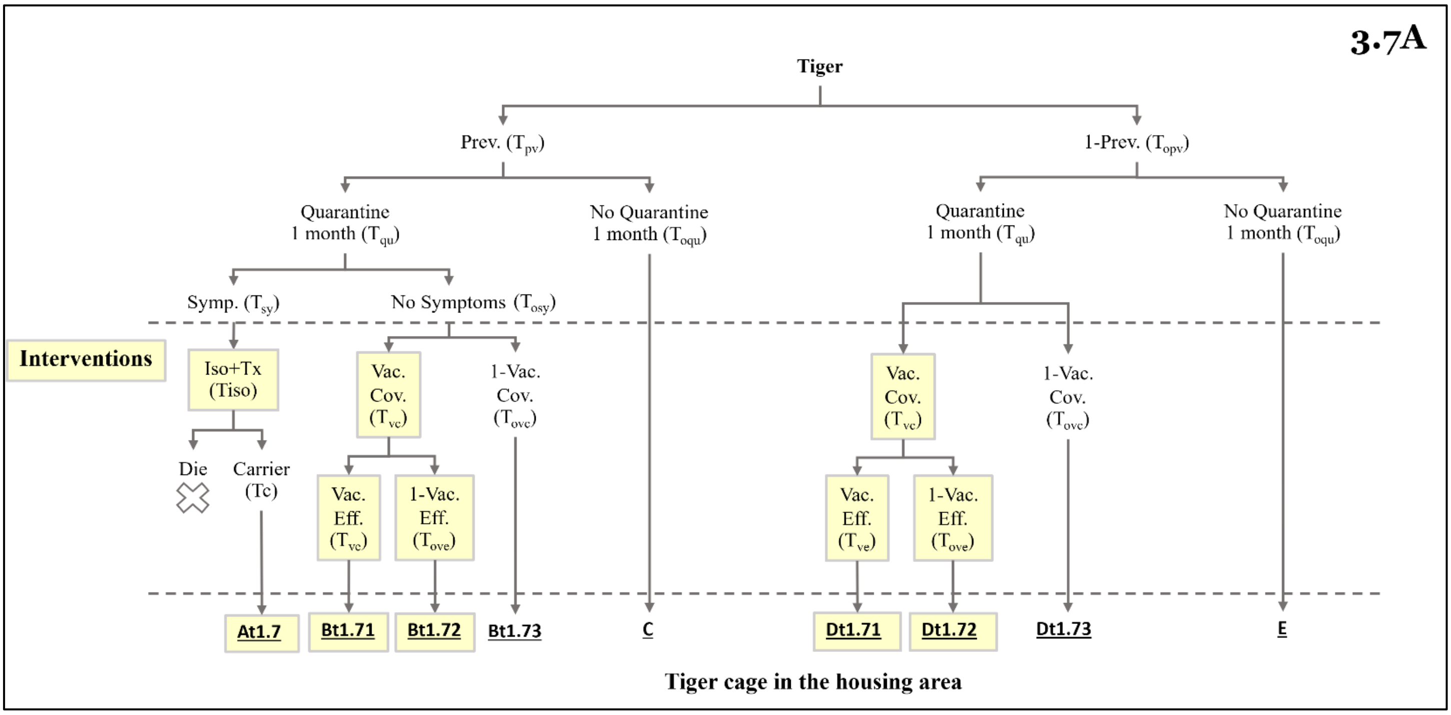

$$P_{TIso+V} = (At1.7 + Bt1.72 + Bt1.73 + C)/(At1.7 + Bt1.71 + Bt1.72 + Bt1.73 + C + Dt1.71 + Dt1.72 + Dt1.73 + E)$$

(equation for Fig. 3.7A)

sub-equations of P_{TIso+V}	$A_{t1.7} = T_{pv} * T_{qu} * T_{sy} * T_{iso} * T_c$
	$B_{t1.71} = T_{pv} * T_{qu} * T_{osy} * T_{vc} * T_{ve}$
	$B_{t1.72} = T_{pv} * T_{qu} * T_{osy} * T_{vc} * 1\text{-}T_{ve}$
	$B_{t1.73} = T_{pv} * T_{qu} * T_{osy} * 1\text{-}T_{vc}$
	$C = T_{pv} * T_{oqu}$
	$D_{t1.71} = T_{opv} * T_{qu} * T_{vc} * T_{ve}$
	$D_{t1.72} = T_{opv} * T_{qu} * T_{vc} * 1\text{-}T_{ve}$
	$D_{t1.73} = T_{opv} * T_{qu} * 1\text{-}T_{vc}$
	$E = T_{opv} * T_{oqu}$

* Abbreviations of probabilities in sub-pathways can be seen in S2 Table 2.

Supporting information

Figs S3

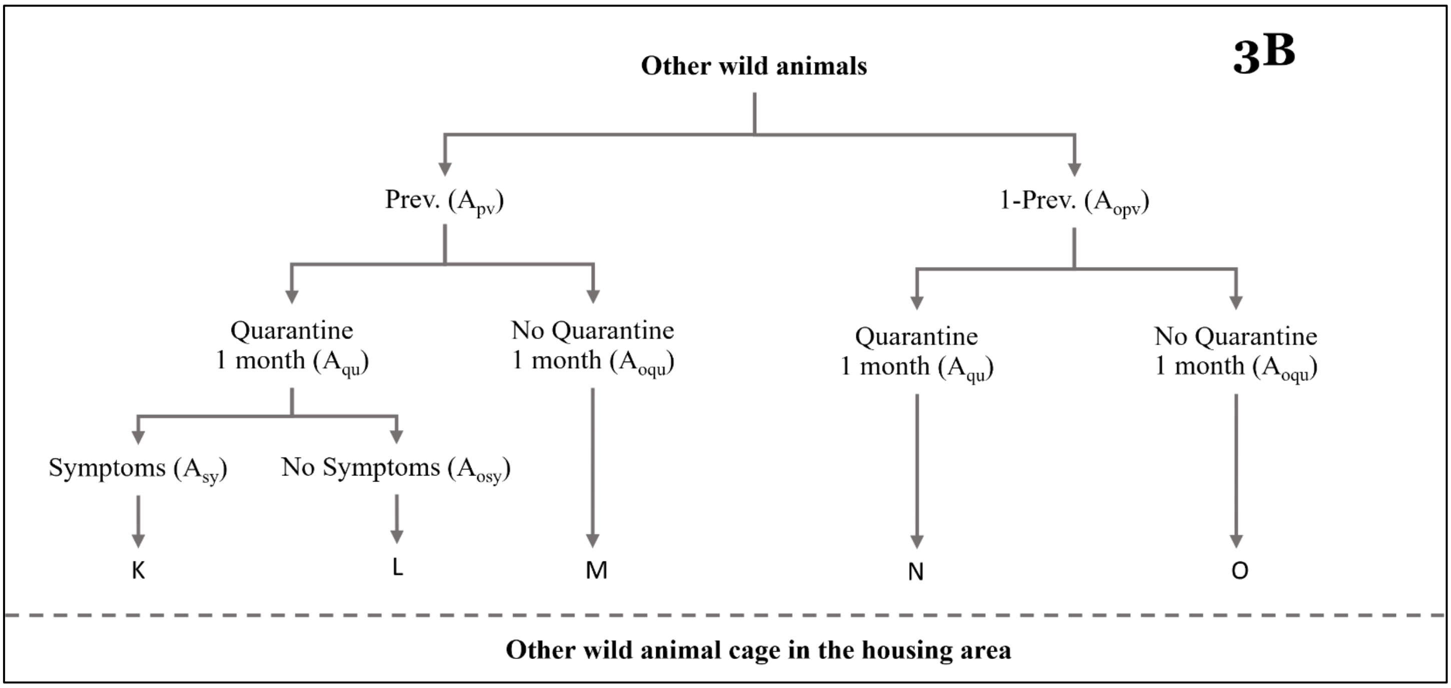

$P_{Animal} = (K + L + M)/(K + L + M + N + O)$ (equation for Fig. 3B)

sub-equations of P_{Animal}	$K = A_{pv} * A_{qu} * A_{sy}$
	$L = A_{pv} * A_{qu} * A_{osy}$
	$M = A_{pv} * A_{oqu}$
	$N = A_{opv} * A_{qu}$
	$O = A_{opv} * A_{oqu}$

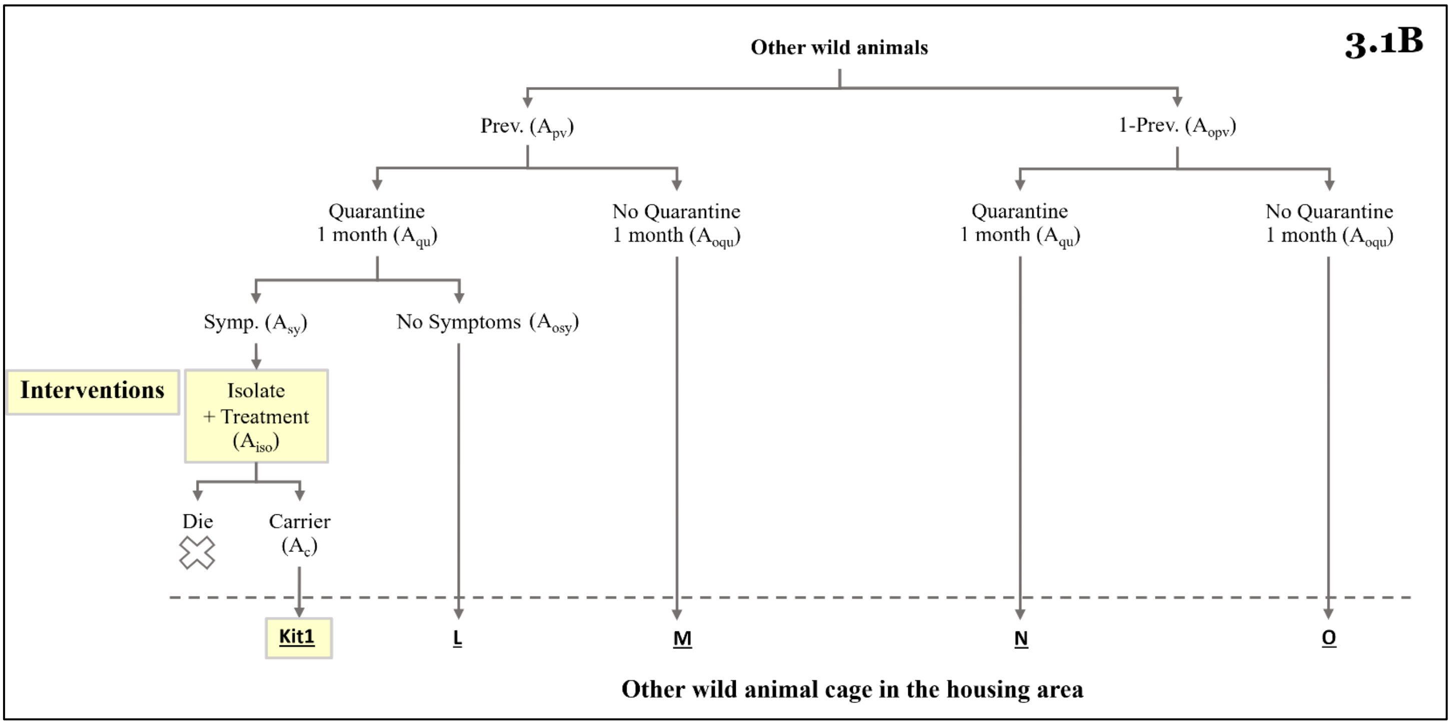

$P_{AIso} = (Kit1 + L + M)/(Kit1 + L + M + N + O)$ (equation for Fig. 3.1B)

sub-equations of P_{AIso}	$K_{it1} = A_{pv} * A_{qu} * A_{sy} * A_{iso} * A_c$
	$L = A_{pv} * A_{qu} * A_{osy}$
	$M = A_{pv} * A_{oqu}$
	$N = A_{opv} * A_{qu}$
	$O = A_{opv} * A_{oqu}$

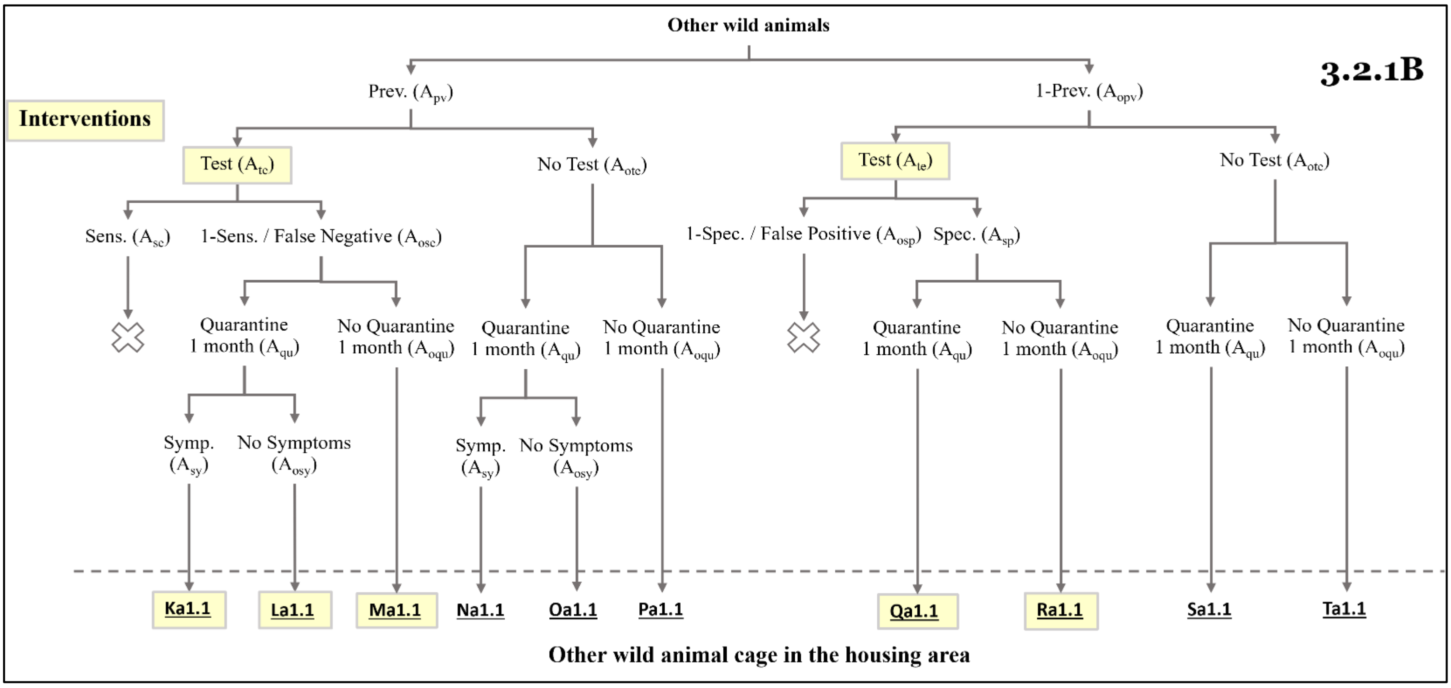

P_{ATori} = $(Ka1.1 + La1.1 + Ma1.1 + Na1.1 + Oa1.1 + Pa1.1)/(Ka1.1 + La1.1 + Ma1.1 + Na1.1 + Oa1.1 + Pa1.1 + Qa1.1 + Ra1.1 + Sa1.1 + Ta1.1)$ (equation for Fig. 3.2.1B)

sub-equations of P_{ATori}	$K_{a1.1} = A_{pv} * A_{te} * A_{ose} * A_{qu} * A_{sy}$
	$L_{a1.1} = A_{pv} * A_{te} * A_{ose} * A_{qu} * A_{osy}$
	$M_{a1.1} = A_{pv} * A_{te} * A_{ose} * A_{oqu}$
	$N_{a1.1} = A_{pv} * A_{ote} * A_{qu} * A_{sy}$
	$O_{a1.1} = A_{pv} * A_{ote} * A_{qu} * A_{osy}$
	$P_{a1.1} = A_{pv} * A_{ote} * A_{oqu}$
	$Q_{a1.1} = A_{opv} * A_{te} * A_{sp} * A_{qu}$
	$R_{a1.1} = A_{opv} * A_{te} * A_{sp} * A_{oqu}$
	$S_{a1.1} = A_{opv} * A_{ote} * A_{qu}$
	$T_{a1.1} = A_{opv} * A_{ote} * A_{oqu}$

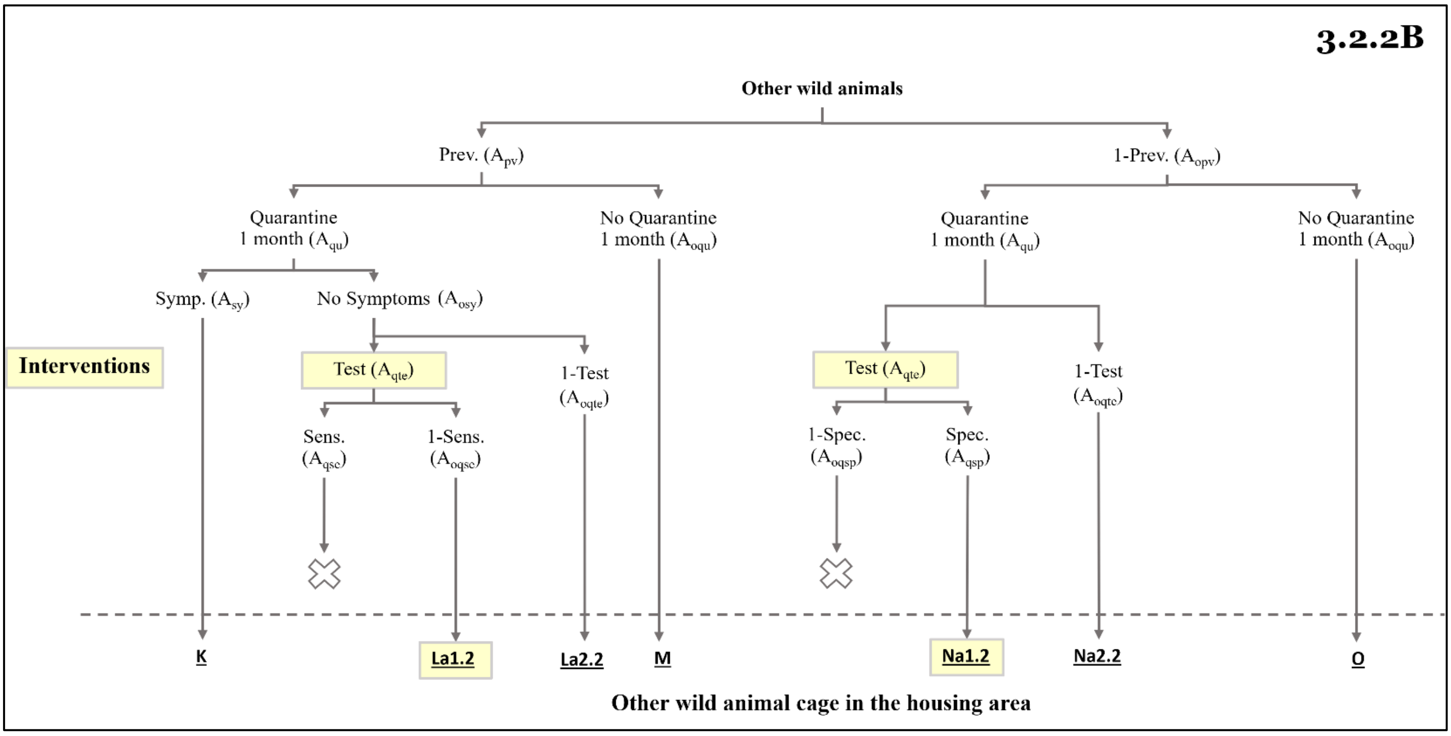

$P_{ATsta} \;=\; (K + La1.2 + La2.2 + M)/(K + La1.2 + La2.2 + M + Na1.2 + Na2.2 + O)$ (equation for Fig. 3.2.2B)

sub-equations of P_{ATsta}	$K = A_{pv} * A_{qu} * A_{sy}$
	$L_{a1.2} = A_{pv} * A_{qu} * A_{osy} * A_{qte} * A_{oqse}$
	$L_{a2.2} = A_{pv} * A_{qu} * A_{osy} * A_{oqte}$
	$M = A_{pv} * A_{oqu}$
	$N_{a1.2} = A_{opv} * A_{qu} * A_{qte} * A_{qsp}$
	$N_{a2.2} = A_{opv} * A_{qu} * A_{oqte}$
	$O = A_{opv} * A_{oqu}$

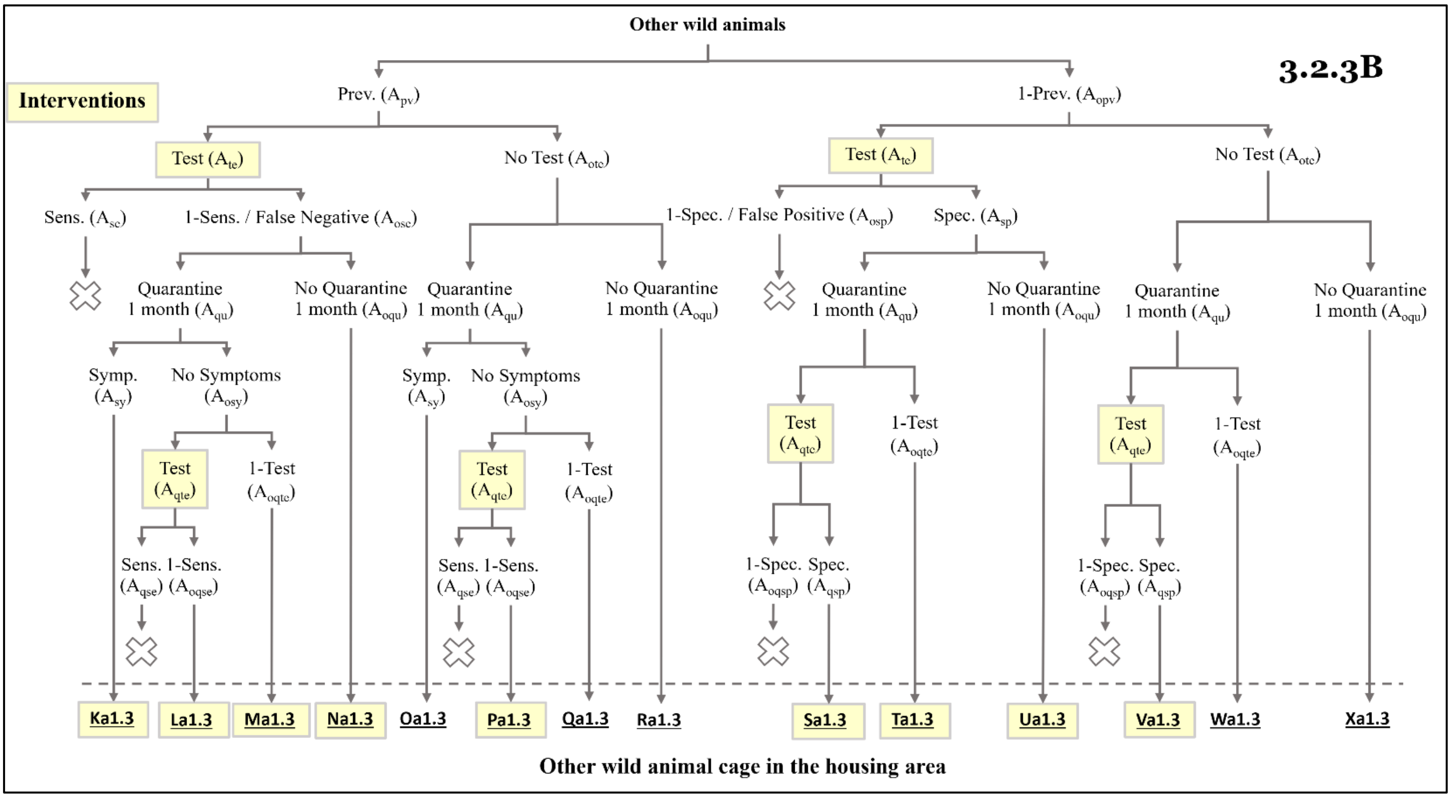

$$P_{ATos} = (Ka1.3 + La1.3 + Ma1.3 + Na1.3 + Oa1.3 + Pa1.3 + Qa1.3 + Ra1.3)/(Ka1.3 + La1.3 + Ma1.3 + Na1.3 + Oa1.3 + Pa1.3 + Qa1.3 + Ra1.3 + Sa1.3 + Ta1.3 + Ua1.3 + Va1.3 + Wa1.3 + Xa1.3)$$

(equation for Fig. 3.2.3B)

sub-equations of P_{ATos}	$K_{a1.3} = A_{pv} * A_{te} * A_{ose} * A_{qu} * A_{sy}$
	$L_{a1.3} = A_{pv} * A_{te} * A_{ose} * A_{qu} * A_{osy} * A_{qte} * A_{oqse}$
	$M_{a1.3} = A_{pv} * A_{te} * A_{ose} * A_{qu} * A_{osy} * A_{oqte}$
	$N_{a1.3} = A_{pv} * A_{te} * A_{ose} * A_{oqu}$
	$O_{a1.3} = A_{pv} * A_{ote} * A_{qu} * A_{sy}$
	$P_{a1.3} = A_{pv} * A_{ote} * A_{qu} * A_{osy} * A_{qte} * A_{oqse}$
	$Q_{a1.3} = A_{pv} * A_{ote} * A_{qu} * A_{osy} * A_{oqte}$
	$R_{a1.3} = A_{pv} * A_{ote} * A_{oqu}$
	$S_{a1.3} = A_{opv} * A_{te} * A_{sp} * A_{qu} * A_{qte} * A_{qsp}$
	$T_{a1.3} = A_{opv} * A_{te} * A_{sp} * A_{qu} * A_{oqte}$
	$U_{a1.3} = A_{opv} * A_{te} * A_{sp} * A_{oqu}$
	$V_{a1.3} = A_{opv} * A_{ote} * A_{qu} * A_{qte} * A_{qsp}$
	$W_{a1.3} = A_{opv} * A_{ote} * A_{qu} * A_{oqte}$
	$X_{a1.3} = A_{opv} * A_{ote} * A_{oqu}$

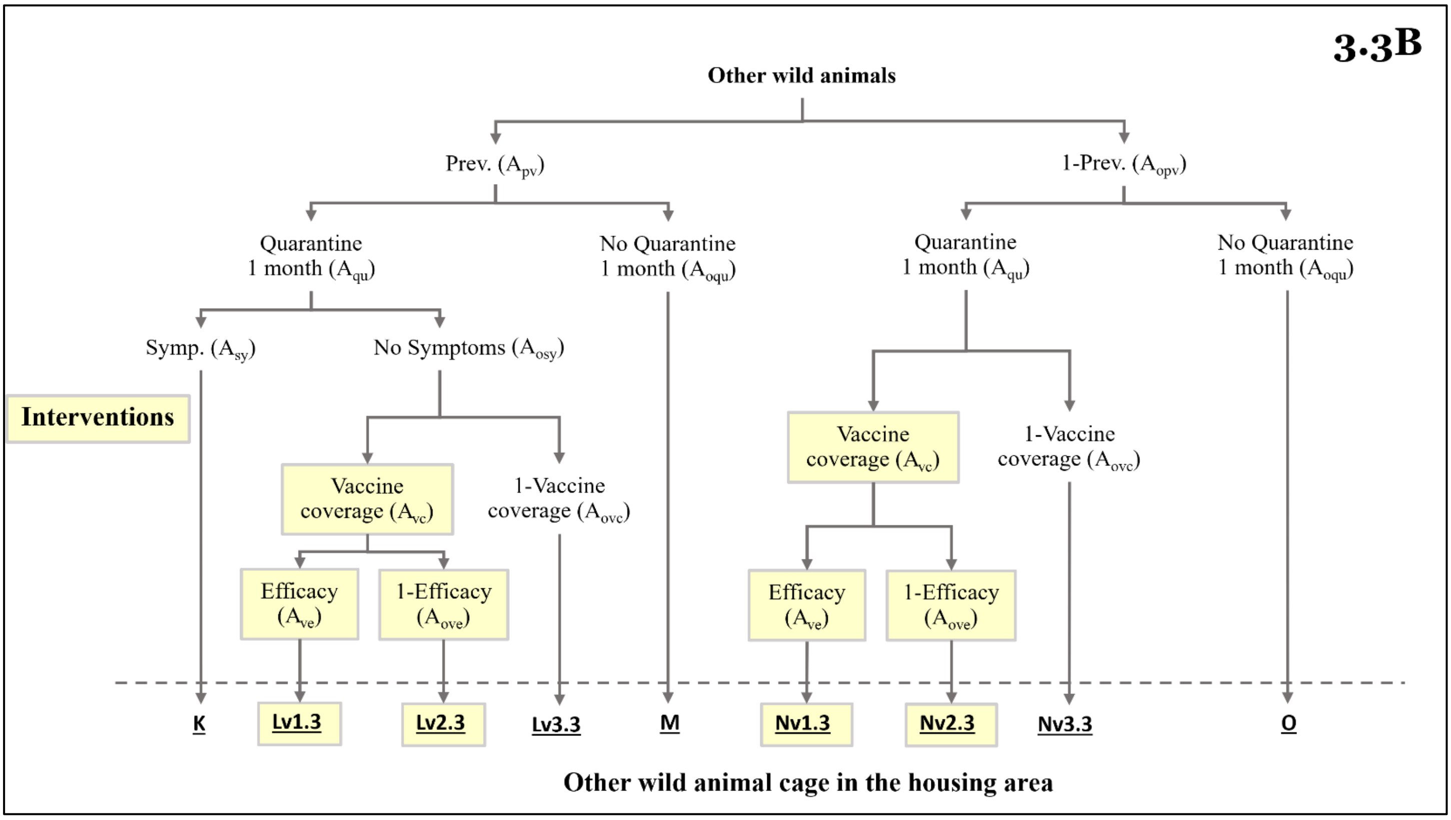

$$P_{AVac} = (K + Lv2.3 + Lv3.3 + M)/(K + Lv1.3 + Lv2.3 + Lv3.3 + M + Nv1.3 + Nv2.3 + Nv3.3 + O)$$

(equation for Fig. 3.3B)

sub-equations of P_{AVac}	$K = A_{pv} * A_{qu} * A_{sy}$
	$L_{v1.3} = A_{pv} * A_{qu} * A_{osy} * A_{vc} * A_{ve}$
	$L_{v2.3} = A_{pv} * A_{qu} * A_{osy} * A_{vc} * (1- A_{ve})$
	$L_{v3.3} = A_{pv} * A_{qu} * A_{osy} * (1- A_{vc})$
	$M = A_{pv} * A_{oqu}$
	$N_{v1.3} = A_{opv} * A_{qu} * A_{vc} * A_{ve}$
	$N_{v2.3} = A_{opv} * A_{qu} * A_{vc} * (1- A_{ve})$
	$N_{v3.3} = A_{opv} * A_{qu} * (1- A_{vc})$
	$O = A_{opv} * A_{oqu}$

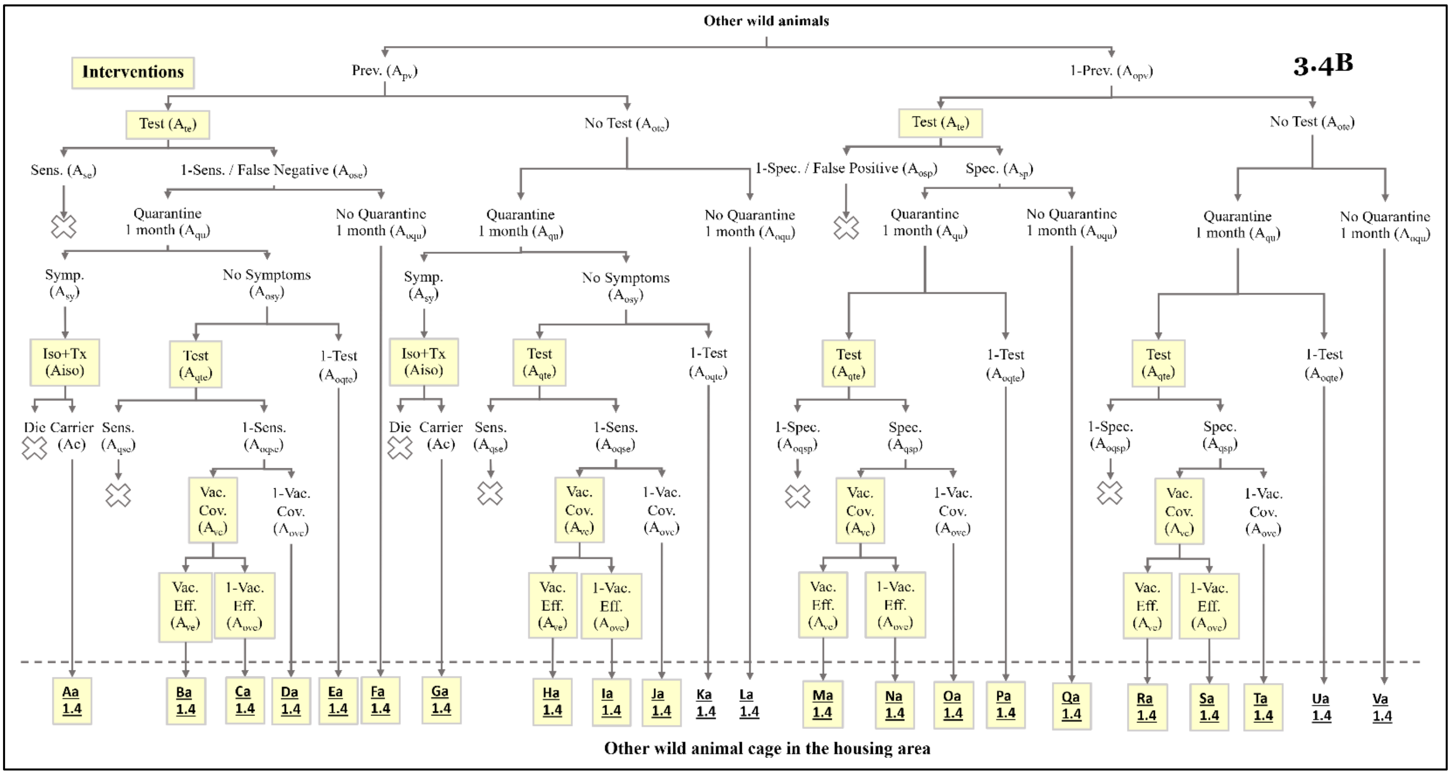

$$P_{ATos+Iso+V} = Aa1.4 + Ca1.4 + Da1.4 + Ea1.4 + Fa1.4 + Ga1.4 + Ia1.4 + Ja1.4 + Ka1.4 + La1.4)/ (Aa1.4 + Ba1.4 + Ca1.4 + Da1.4 + Ea1.4 + Fa1.4 + Ga1.4 + Ha1.4 + Ia1.4 + Ja1.4 + Ka1.4 + La1.4 + Ma1.4 + Na1.4 + Oa1.4 + Pa1.4 + Qa1.4 + Ra1.4 + Sa1.4 + Ta1.4 + Ua1.4 + Va1.4)$$

(equation for Fig. 3.4B)

sub-equations of $P_{ATos+Iso+V}$	$A_{a1.4} = A_{pv} * A_{te} * A_{ose} * A_{qu} * A_{sy} * A_{iso} * A_{c}$
	$B_{a1.4} = A_{pv} * A_{te} * A_{ose} * A_{qu} * A_{osy} * A_{qte} * A_{oqse} * A_{vc} * A_{ve}$
	$C_{a1.4} = A_{pv} * A_{te} * A_{ose} * A_{qu} * A_{osy} * A_{qte} * A_{oqse} * A_{vc} * 1\text{-}A_{ve}$
	$D_{a1.4} = A_{pv} * A_{te} * A_{ose} * A_{qu} * A_{osy} * A_{qte} * A_{oqse} * 1\text{-}A_{vc}$
	$E_{a1.4} = A_{pv} * A_{te} * A_{ose} * A_{qu} * A_{osy} * A_{oqte}$
	$F_{a1.4} = A_{pv} * A_{te} * A_{ose} * A_{oqu}$
	$G_{a1.4} = A_{pv} * A_{ote} * A_{qu} * A_{sy} * A_{iso} * A_{c}$
	$H_{a1.4} = A_{pv} * A_{ote} * A_{qu} * A_{osy} * A_{qte} * A_{oqse} * A_{vc} * A_{ve}$
	$I_{a1.4} = A_{pv} * A_{ote} * A_{qu} * A_{osy} * A_{qte} * A_{oqse} * A_{vc} * 1\text{-}A_{ve}$
	$J_{a1.4} = A_{pv} * A_{ote} * A_{qu} * A_{osy} * A_{qte} * A_{oqse} * 1\text{-}A_{vc}$
	$K_{a1.4} = A_{pv} * A_{ote} * A_{qu} * A_{osy} * A_{oqte}$
	$L_{a1.4} = A_{pv} * A_{ote} * A_{oqu}$

sub-equations of $P_{ATos+Iso+V}$	$M_{a1.4} = A_{opv} * A_{te} * A_{sp} * A_{qu} * A_{qte} * A_{qsp} * A_{vc} * A_{ve}$
	$N_{a1.4} = A_{opv} * A_{te} * A_{sp} * A_{qu} * A_{qte} * A_{qsp} * A_{vc} * 1\text{-}A_{ve}$
	$O_{a1.4} = A_{opv} * A_{te} * A_{sp} * A_{qu} * A_{qte} * A_{qsp} * 1\text{-}A_{vc}$
	$P_{a1.4} = A_{opv} * A_{te} * A_{sp} * A_{qu} * A_{oqte}$
	$Q_{a1.4} = A_{opv} * A_{te} * A_{sp} * A_{oqu}$
	$R_{a1.4} = A_{opv} * A_{ote} * A_{qu} * A_{qte} * A_{qsp} * A_{vc} * A_{ve}$
	$S_{a1.4} = A_{opv} * A_{ote} * A_{qu} * A_{qte} * A_{qsp} * A_{vc} * 1\text{-}A_{ve}$
	$T_{a1.4} = A_{opv} * A_{ote} * A_{qu} * A_{qte} * A_{qsp} * 1\text{-}A_{vc}$
	$U_{a1.4} = A_{opv} * A_{ote} * A_{qu} * A_{oqte}$
	$V_{a1.4} = A_{opv} * A_{ote} * A_{oqu}$

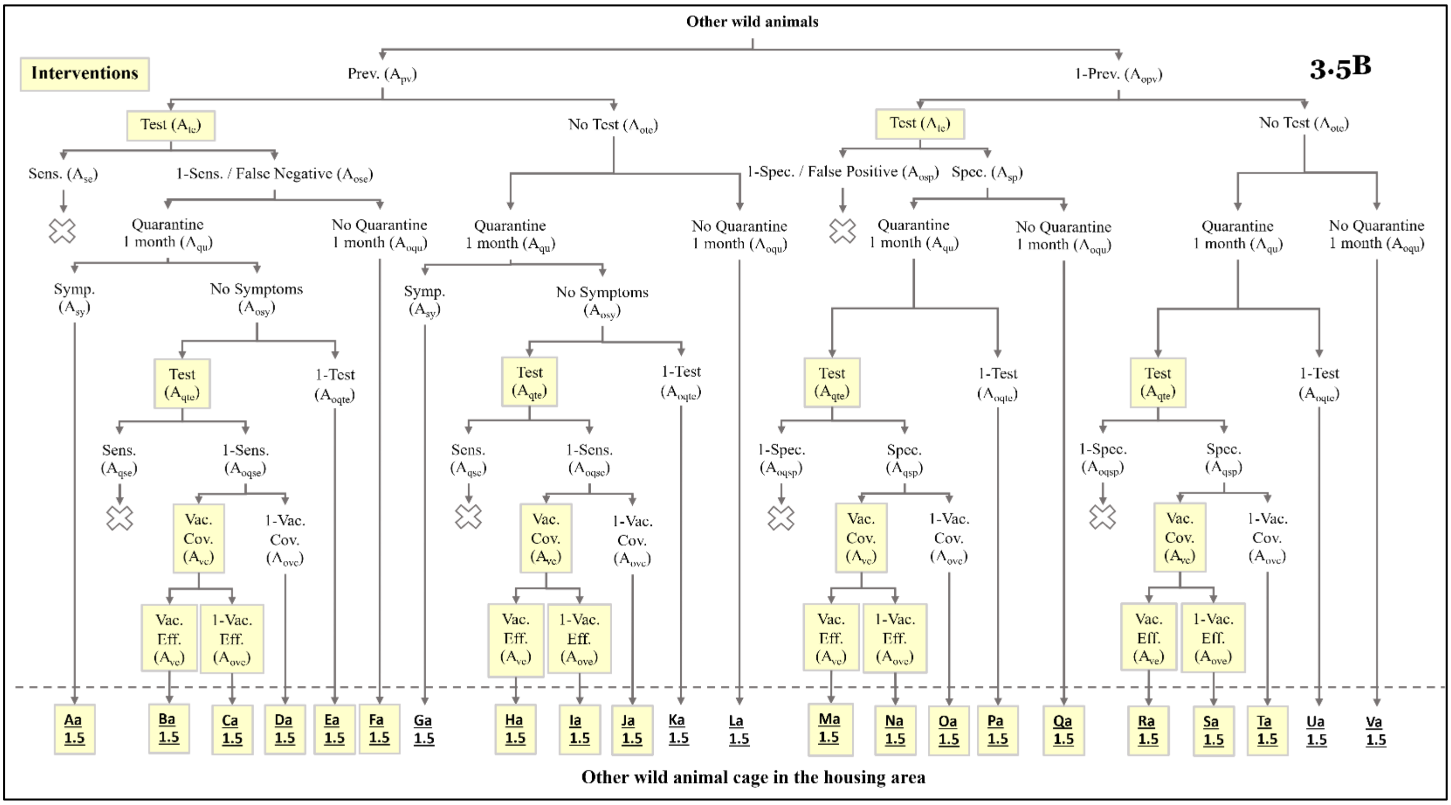

$$P_{ATos+V} = (Aa1.5 + Ca1.5 + Da1.5 + Ea1.5 + Fa1.5 + Ga1.5 + Ia1.5 + Ja1.5 + Ka1.5 + La1.5) / (Aa1.5 + Ba1.5 + Ca1.5 + Da1.5 + Ea1.5 + Fa1.5 + Ga1.5 + Ha1.5 + Ia1.5 + Ja1.5 + Ka1.5 + La1.5 + Ma1.5 + Na1.5 + Oa1.5 + Pa1.5 + Qa1.5 + Ra1.5 + Sa1.5 + Ta1.5 + Ua1.5 + Va1.5)$$

(equation for Fig. 3.5B)

sub-equations of P_{ATos+V}	$A_{a1.5} = A_{pv} * A_{te} * A_{ose} * A_{qu} * A_{sy}$
	$B_{a1.5} = A_{pv} * A_{te} * A_{ose} * A_{qu} * A_{osy} * A_{qte} * A_{oqse} * A_{vc} * A_{ve}$
	$C_{a1.5} = A_{pv} * A_{te} * A_{ose} * A_{qu} * A_{osy} * A_{qte} * A_{oqse} * A_{vc} * 1\text{-}A_{ve}$
	$D_{a1.5} = A_{pv} * A_{te} * A_{ose} * A_{qu} * A_{osy} * A_{qte} * A_{oqse} * 1\text{-}A_{vc}$
	$E_{a1.5} = A_{pv} * A_{te} * A_{ose} * A_{qu} * A_{osy} * A_{oqte}$
	$F_{a1.5} = A_{pv} * A_{te} * A_{ose} * A_{oqu}$
	$G_{a1.5} = A_{pv} * A_{ote} * A_{qu} * A_{sy}$
	$H_{a1.5} = A_{pv} * A_{ote} * A_{qu} * A_{osy} * A_{qte} * A_{oqse} * A_{vc} * A_{ve}$
	$I_{a1.5} = A_{pv} * A_{ote} * A_{qu} * A_{osy} * A_{qte} * A_{oqse} * A_{vc} * 1\text{-}A_{ve}$
	$J_{a1.5} = A_{pv} * A_{ote} * A_{qu} * A_{osy} * A_{qte} * A_{oqse} * 1\text{-}A_{vc}$
	$K_{a1.5} = A_{pv} * A_{ote} * A_{qu} * A_{osy} * A_{oqte}$
	$L_{a1.5} = A_{pv} * A_{ote} * A_{oqu}$
	$M_{a1.5} = A_{opv} * A_{te} * A_{sp} * A_{qu} * A_{qte} * A_{qsp} * A_{vc} * A_{ve}$

sub-equations of P_{ATos+V}	$N_{a1.5} = A_{opv} * A_{te} * A_{sp} * A_{qu} * A_{qte} * A_{qsp} * A_{vc} * 1\text{-}A_{ve}$
	$O_{a1.5} = A_{opv} * A_{te} * A_{sp} * A_{qu} * A_{qte} * A_{qsp} * 1\text{-}A_{vc}$
	$P_{a1.5} = A_{opv} * A_{te} * A_{sp} * A_{qu} * A_{oqte}$
	$Q_{a1.5} = A_{opv} * A_{te} * A_{sp} * A_{oqu}$
	$R_{a1.5} = A_{opv} * A_{ote} * A_{qu} * A_{qte} * A_{qsp} * A_{vc} * A_{ve}$
	$S_{a1.5} = A_{opv} * A_{ote} * A_{qu} * A_{qte} * A_{qsp} * A_{vc} * 1\text{-}A_{ve}$
	$T_{a1.5} = A_{opv} * A_{ote} * A_{qu} * A_{qte} * A_{qsp} * 1\text{-}A_{vc}$
	$U_{a1.5} = A_{opv} * A_{ote} * A_{qu} * A_{oqte}$
	$V_{a1.5} = A_{opv} * A_{ote} * A_{oqu}$

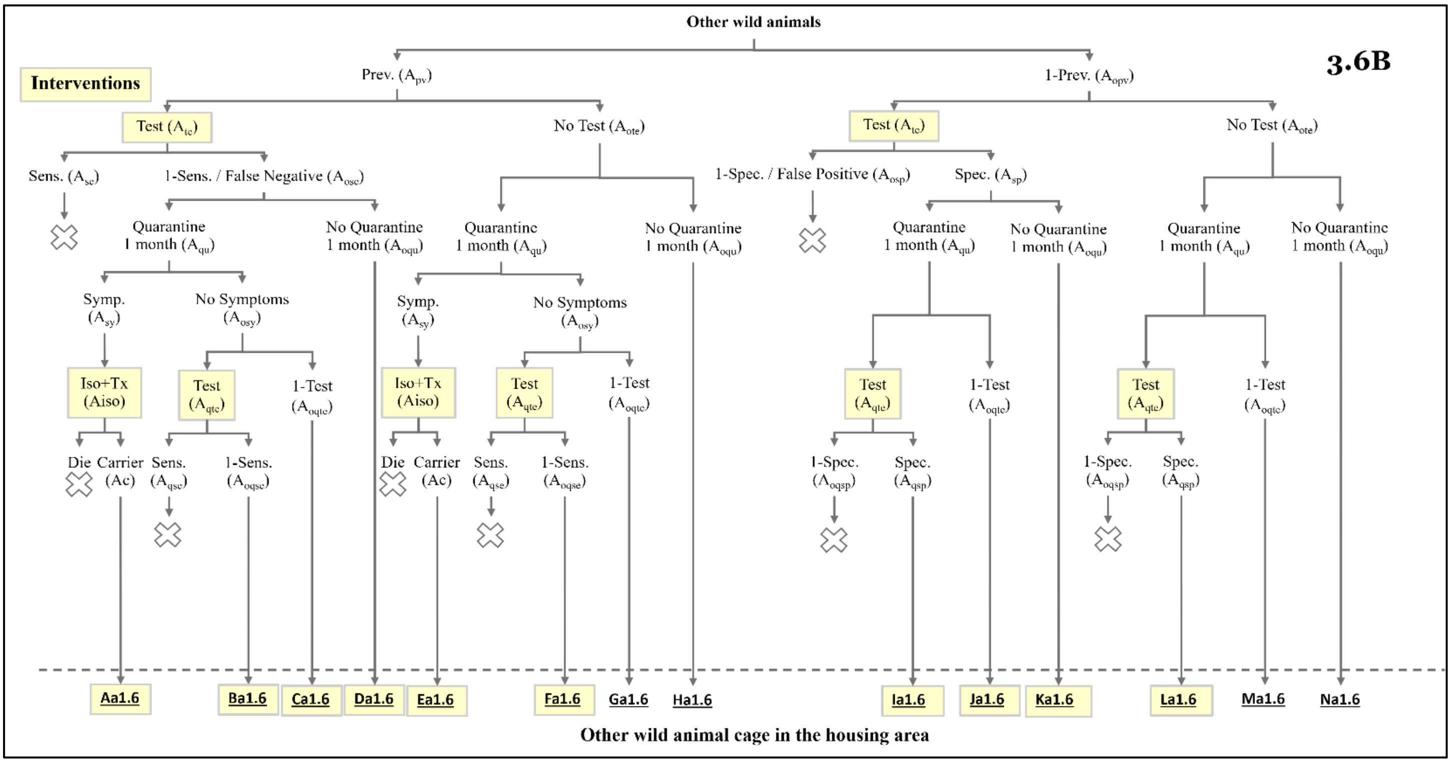

$$P_{ATos+Iso} = (Aa1.6 + Ba1.6 + Ca1.6 + Da1.6 + Ea1.6 + Fa1.6 + Ga1.6 + Ha1.6) / (Aa1.6 + Ba1.6 + Ca1.6 + Da1.6 + Ea1.6 + Fa1.6 + Ga1.6 + Ha1.6 + Ia1.6 + Ja1.6 + Ka1.6 + La1.6 + Ma1.6 + Na1.6)$$

(equation for Fig. 3.6B)

sub-equations of $P_{ATos+Iso}$	$A_{a1.6} = A_{pv} * A_{te} * A_{ose} * A_{qu} * A_{sy} * A_{iso} * A_{c}$
	$B_{a1.6} = A_{pv} * A_{te} * A_{ose} * A_{qu} * A_{osy} * A_{qte} * A_{oqse}$
	$C_{a1.6} = A_{pv} * A_{te} * A_{ose} * A_{qu} * A_{osy} * A_{oqte}$
	$D_{a1.6} = A_{pv} * A_{te} * A_{ose} * A_{oqu}$
	$E_{a1.6} = A_{pv} * A_{ote} * A_{qu} * A_{sy} * A_{iso} * A_{c}$
	$F_{a1.6} = A_{pv} * A_{ote} * A_{qu} * A_{osy} * A_{qte} * A_{oqse}$
	$G_{a1.6} = A_{pv} * A_{ote} * A_{qu} * A_{osy} * A_{oqte}$
	$H_{a1.6} = A_{pv} * A_{ote} * A_{oqu}$
	$I_{a1.6} = A_{opv} * A_{te} * A_{sp} * A_{qu} * A_{qte} * A_{qsp}$
	$J_{a1.6} = A_{opv} * A_{te} * A_{sp} * A_{qu} * A_{oqte}$
	$K_{a1.6} = A_{opv} * A_{te} * A_{sp} * A_{oqu}$
	$L_{a1.6} = A_{opv} * A_{ote} * A_{qu} * A_{qte} * A_{qsp}$
	$M_{a1.6} = A_{opv} * A_{ote} * A_{qu} * A_{oqte}$
	$N_{a1.6} = A_{opv} * A_{ote} * A_{oqu}$

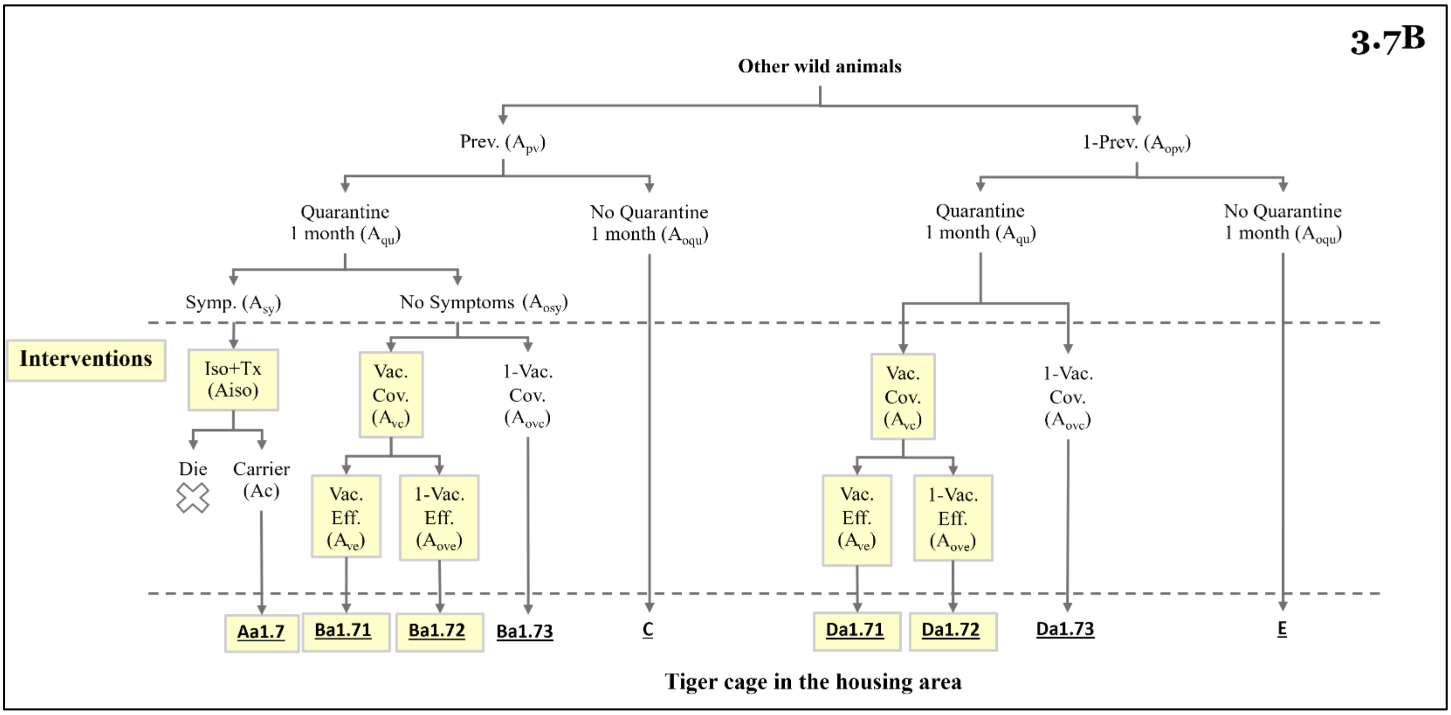

P_{AIso+V} = $(Aa1.7 + Ba1.72 + Ba1.73 + C)/(Aa1.7 + Ba1.71 + Ba1.72 + Ba1.73 + C + Da1.71 + Da1.72 + Da1.73 + E)$ (equation for Fig. 3.7B)

sub-equations of P_{AIso+V}	$A_{a1.7} = A_{pv} * A_{qu} * A_{sy} * A_{iso} * A_{c}$
	$B_{a1.71} = A_{pv} * A_{qu} * A_{osy} * A_{vc} * A_{ve}$
	$B_{a1.72} = A_{pv} * A_{qu} * A_{osy} * A_{vc} * 1\text{-}A_{ve}$
	$B_{a1.73} = A_{pv} * A_{qu} * A_{osy} * 1\text{-}A_{vc}$
	$C = A_{pv} * A_{oqu}$
	$D_{a1.71} = A_{opv} * A_{qu} * A_{vc} * A_{ve}$
	$D_{a1.72} = A_{opv} * A_{qu} * A_{vc} * 1\text{-}A_{ve}$
	$D_{a1.73} = A_{opv} * A_{qu} * 1\text{-}A_{vc}$
	$E = A_{opv} * A_{oqu}$

* Abbreviations of probabilities in sub-pathways can be seen in S2 Table 3.

Supporting information

Figs S4

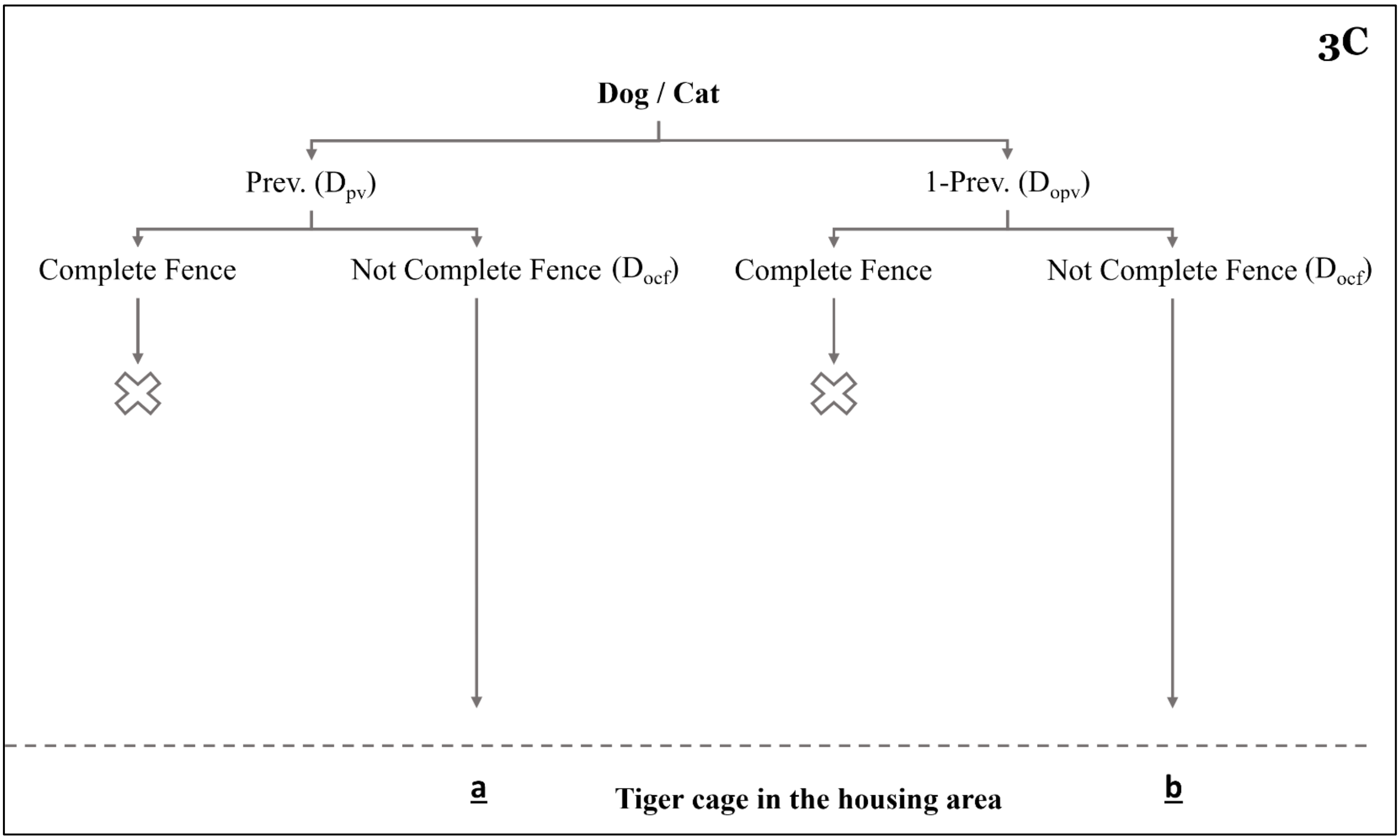

$P_{Dogcat} \quad = \quad a/(a+b)$ (equation for Fig. 3C)

sub-equations of P_{Dogcat}	$a = D_{pv} * D_{ocf}$
	$b = D_{opv} * D_{ocf}$

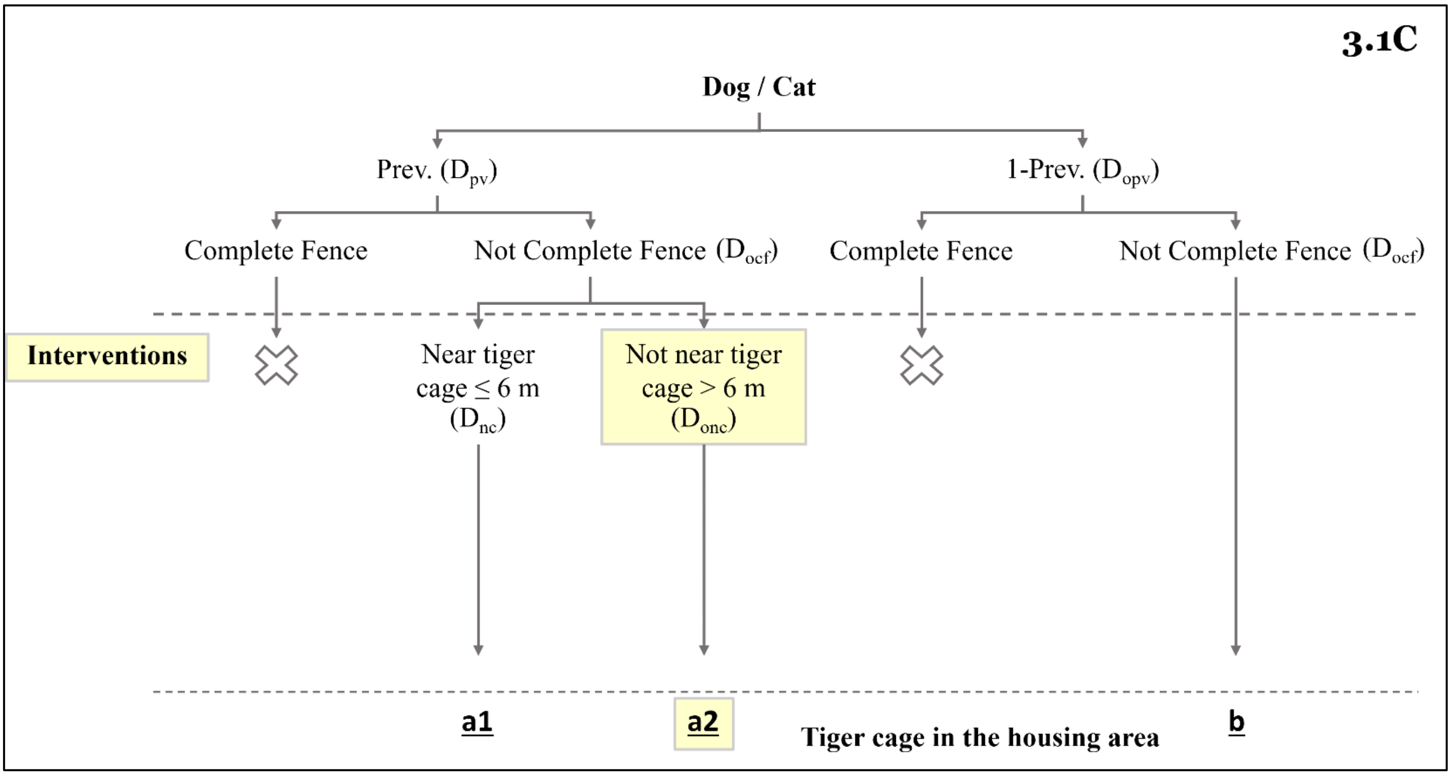

$P_{Dogcati} = a1/(a1 + a2 + b)$ (equation for Fig. 3.1C)

sub-equations of P_{Dogcat}	$a1 = D_{pv} * D_{ocf} * D_{nc}$
	$a2 = D_{pv} * D_{ocf} * D_{onc}$
	$b = D_{opv} * D_{ocf}$

* Abbreviations of probabilities in sub-pathways can be seen in S2 Table 4.

Supporting information

Figs S5

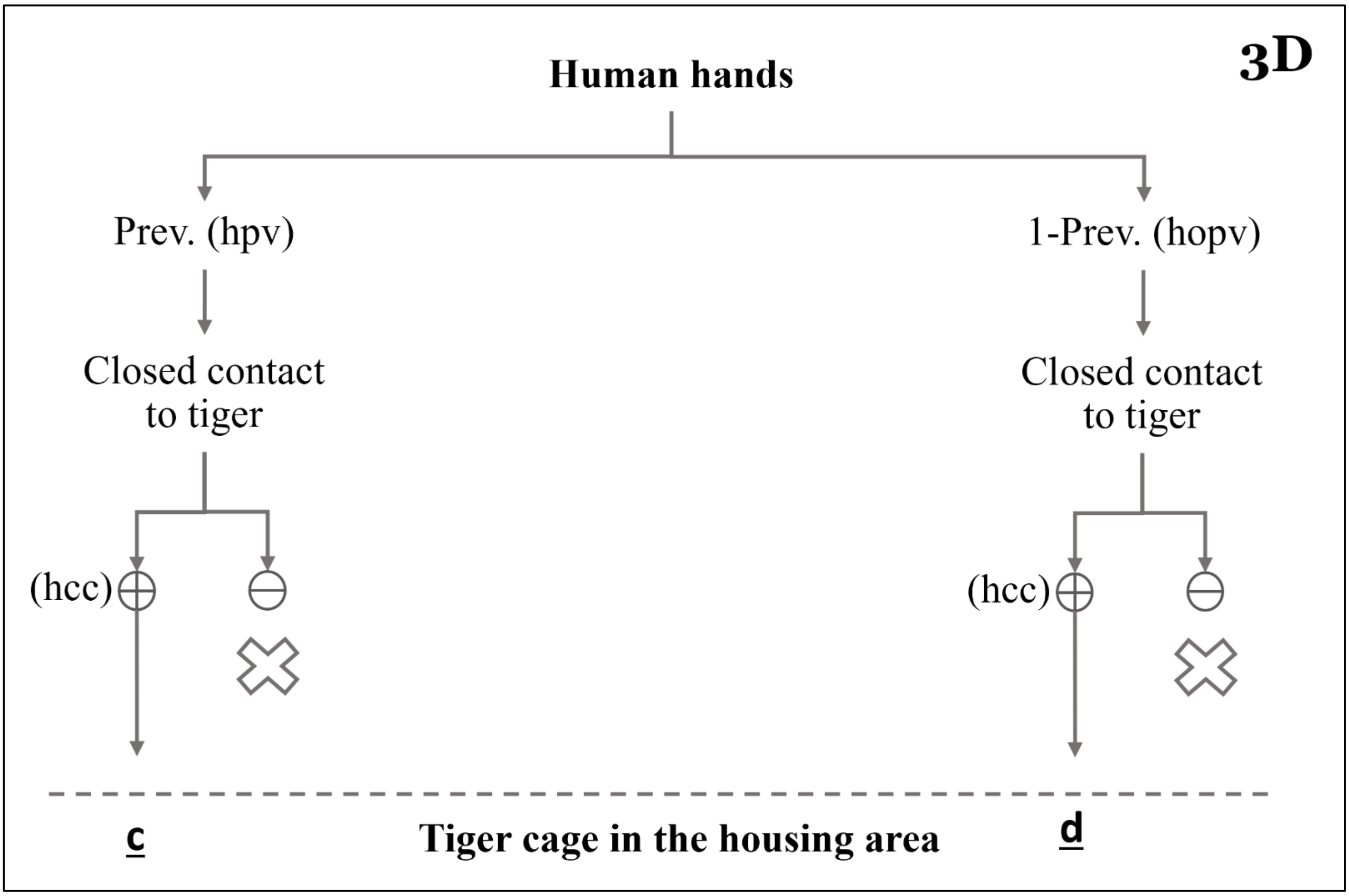

$P_{Hand} = c/(c+d)$ (equation for Fig. 3D)

sub-equations of P_{Hand}	$c = H_{pv} * H_{cc}$
	$d = H_{opv} * H_{cc}$

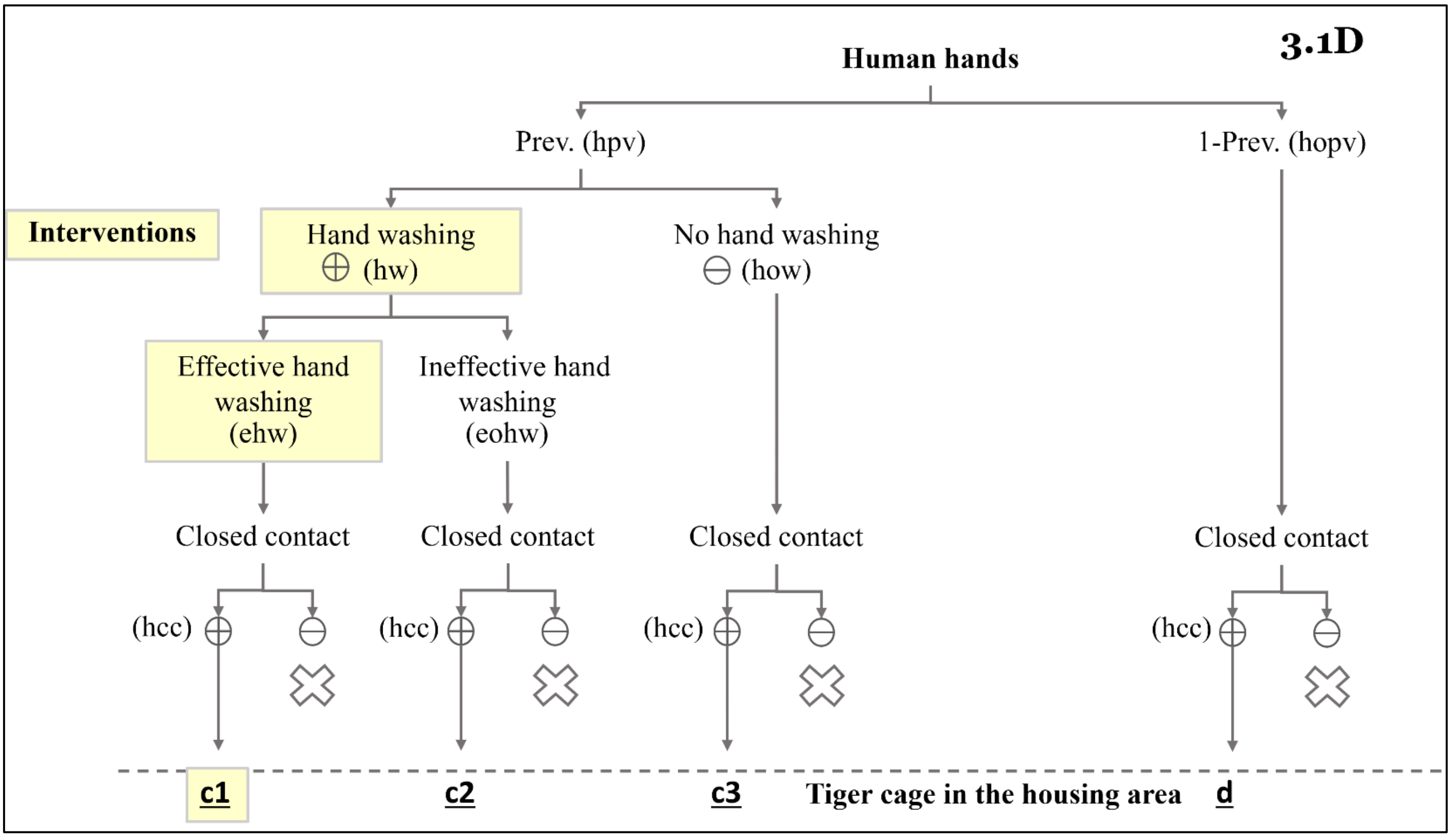

P_{Handi} = (c2 + c3)/(c1 + c2 + c3 + d) (equation for Fig. 3.1D)

sub-equations of P_{Handi}	c1 = $H_{pv} * H_{w} * E_{hw} * H_{cc}$
	c2 = $H_{pv} * H_{w} * E_{ohw} * H_{cc}$
	c3 = $H_{pv} * H_{ow} * H_{cc}$
	d = $H_{opv} * H_{cc}$

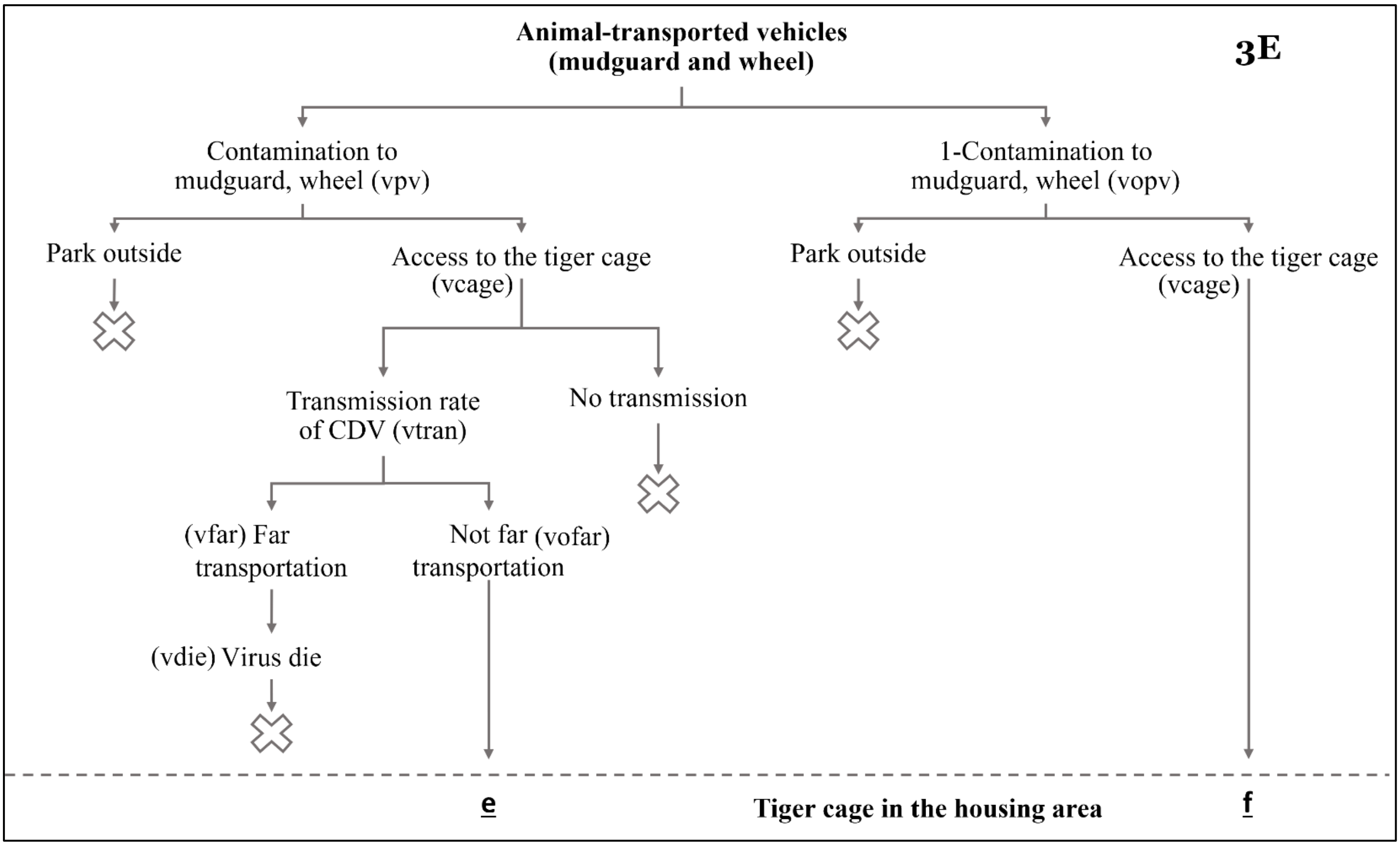

$P_{Vehicle} = e/(e+f)$ (equation for Fig. 3E)

sub-equations of $P_{Vehicle}$	$e = V_{pv} * V_{cage} * V_{tran} * V_{ofar}$
	$f = V_{opv} * V_{cage}$

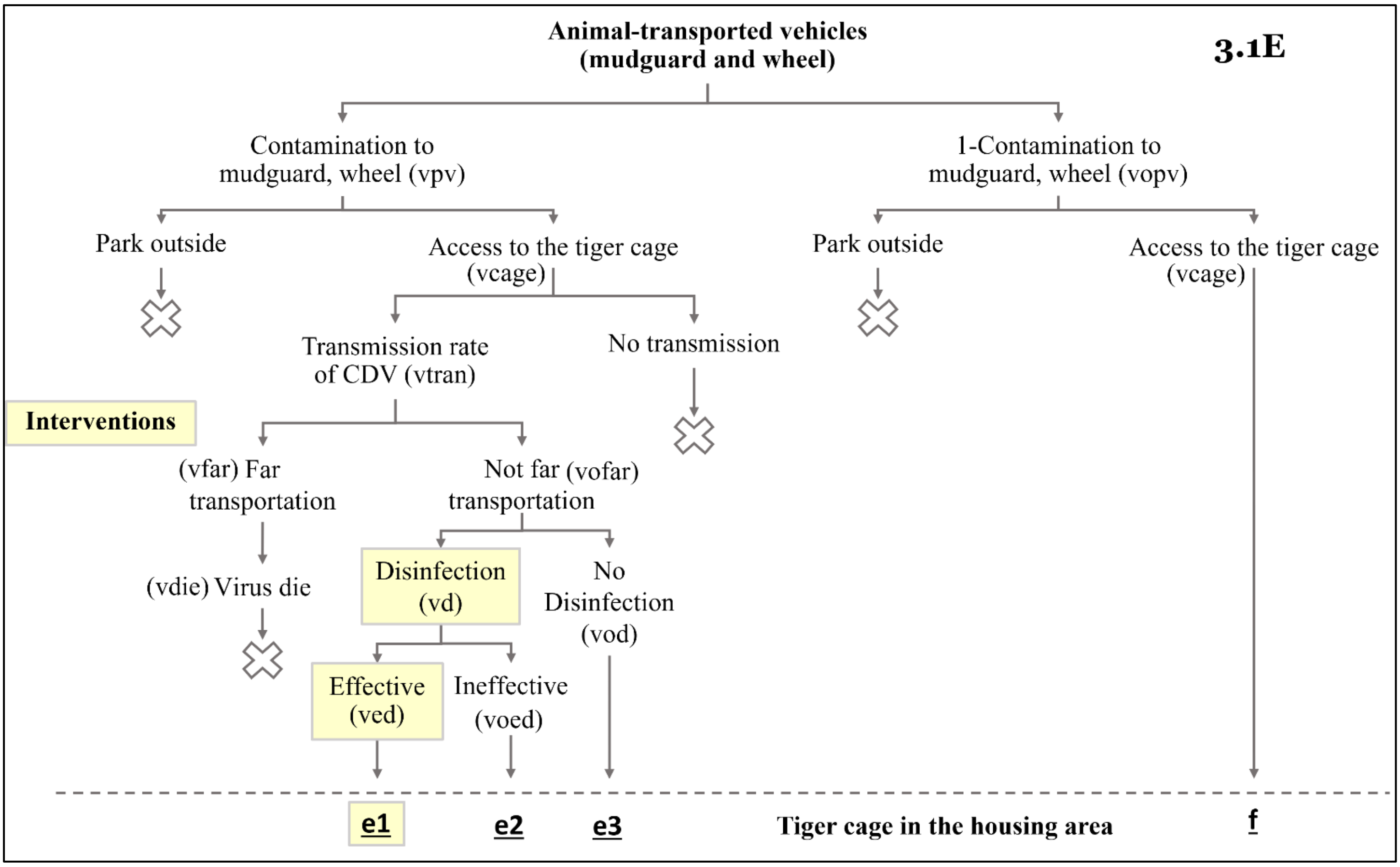

$$P_{Vehiclei} = (e2 + e3)/(e1 + e2 + e3 + f)$$ (equation for Fig. 3.1E)

sub-equations of $P_{Vehiclei}$	e1 = $V_{pv} * V_{cage} * V_{tran} * V_{ofar} * V_{d} * V_{ed}$
	e2 = $V_{pv} * V_{cage} * V_{tran} * V_{ofar} * V_{d} * V_{oed}$
	e3 = $V_{pv} * V_{cage} * V_{tran} * V_{ofar} * V_{od}$
	f = $V_{opv} * V_{cage}$

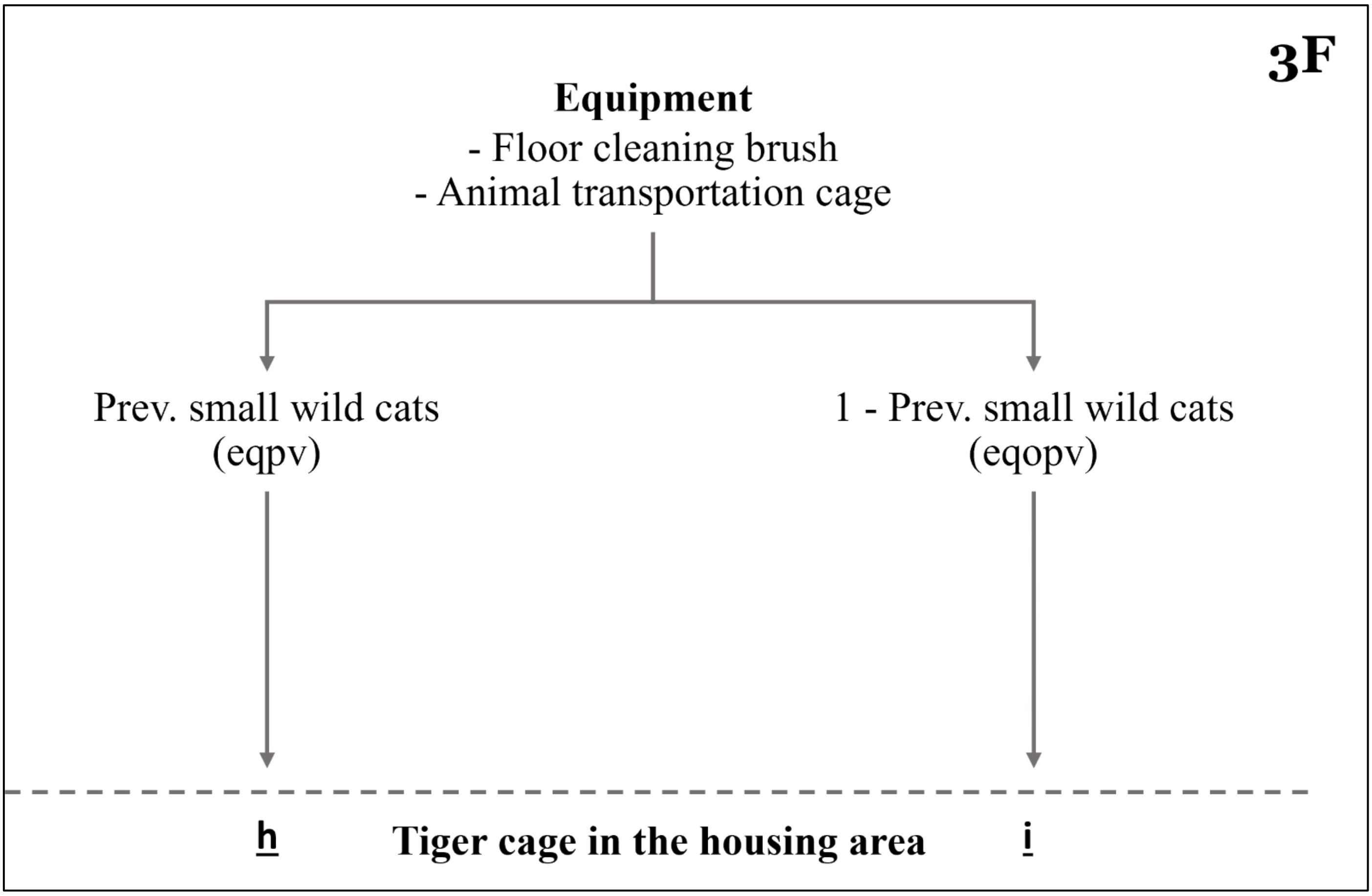

$P_{Equip} = h/(h+i)$ (equation for Fig. 3F)

sub-equations of P_{Equip}	$h = E_{qpv}$
	$i = E_{qopv}$

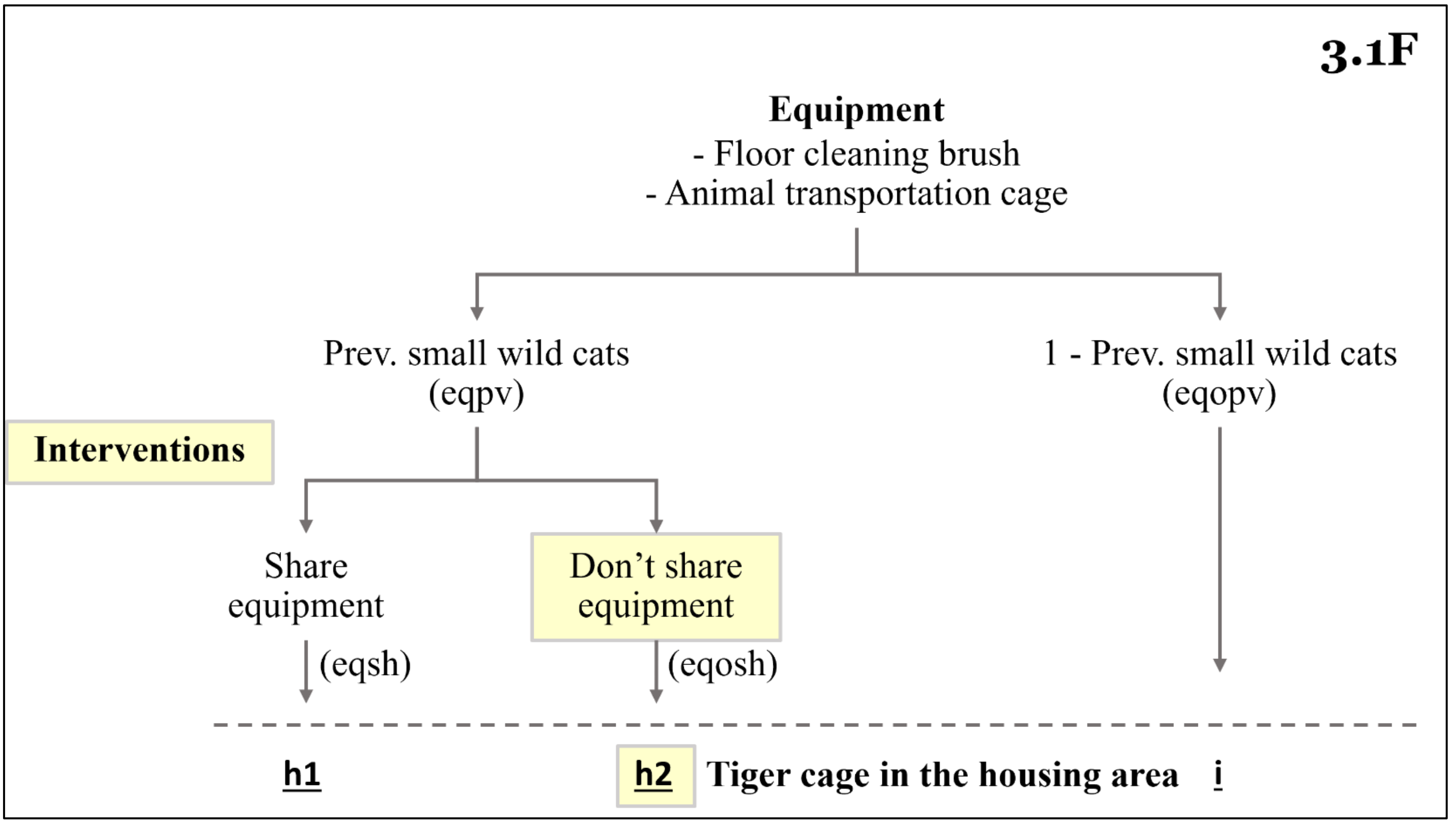

$P_{Equipi} \quad = \quad h1/(h1 + h2 + i)$ (equation for Fig. 3.1F)

sub-equations of P_{Equipi}	$h1 = E_{qpv} * E_{qsh}$
	$h2 = E_{qpv} * E_{qosh}$
	$i = E_{qopv}$

* Abbreviations of probabilities in sub-pathways can be seen in S2 Table 4.

Supporting information

S6 Tables 2-4

Table 2. Description of input parameters and probabilities utilized in the quantitative stochastic models for entry assessment of the risk of CDV introduction into wildlife stations through tiger infections.

Notation	Definition	Parametrization	Source	Values
**Entry of CDV-infected tigers**				
T_{pv}	CDV prevalence in tigers	Beta (α_1, α_2)	[1]	Beta (13, 102)
T_{opv}	non-CDV prevalence in tigers	Beta (α_1, α_2)	[1]	$1 - T_{pv}$
T_{te}	tiger was tested CDV screening before entering	PERT (min, most likely, max)	questionnaire	PERT (0.000, 0.141, 0.423)
T_{ote}	tiger was not tested CDV screening before entering	PERT (min, most likely, max)	-	$1 - T_{te}$
T_{se}	sensitivity of RT-PCR test for CDV	Uniform (min, max)	[2-4]	Uniform (0.819, 1.000)
T_{ose}	false negative of RT-PCR test for CDV	Uniform (min, max)	-	$1 - T_{se}$
T_{sp}	specificity of RT-PCR test for CDV	Uniform (min, max)	[2-4]	Uniform (0.853, 1.000)
T_{qu}	tiger had been quarantined for 30 days	Uniform (min, max)	questionnaire	Uniform (0.950, 1.000)
T_{oqu}	tiger had not been quarantined for 30 days	Uniform (min, max)	-	$1 - T_{qu}$
T_{sy}	tiger has CDV symptoms	Uniform (min, max)	[1, 5-6]	Uniform (0.473, 0.546)
T_{osy}	tiger has no CDV symptoms	Uniform (min, max)	-	$1 - T_{sy}$
T_{iso}	isolate & treat CDV-symptomatic tiger	Uniform (min, max)	questionnaire	Uniform (0.950, 1.000)
T_c	tiger carrier after recovery from CDV	Uniform (min, max)	[1, 7-8]	Uniform (0.300, 0.370)

Notation	Definition	Parametrization	Source	Values
**Entry of CDV-infected tigers**				
T_{qte}	tiger was tested CDV with RT-PCR during quarantine	PERT (min, most likely, max)	questionnaire	PERT (0.000, 0.141, 0.423)
T_{oqte}	tiger was not tested CDV with RT-PCR during quarantine	PERT (min, most likely, max)	-	$1 - T_{qte}$
T_{qse}	sensitivity of RT-PCR test for CDV during quarantine	Uniform (min, max)	[2-4]	Uniform (0.819, 1.000)
T_{oqse}	false negative of RT-PCR test for CDV during quarantine	Uniform (min, max)	-	$1 - T_{qse}$
T_{qsp}	specificity of RT-PCR test for CDV during quarantine	Uniform (min, max)	[2-4]	Uniform (0.853, 1.000)
T_{oqsp}	false positive of RT-PCR test for CDV during quarantine	Uniform (min, max)	-	$1 - T_{qsp}$
T_{ve}	CDV vaccine efficacy in tiger (Recombinant vaccine)	Beta (α_1, α_2)	[1]	Beta (21, 81)
T_{ove}	CDV vaccine without efficacy in tiger	Beta (α_1, α_2)	-	$1 - T_{ve}$
T_{vc}	CDV vaccine coverage in tiger	Beta (α_1, α_2)	questionnaire	Beta (13, 152)
T_{ovc}	did not give the vaccine in tiger	Beta (α_1, α_2)	-	$1 - T_{vc}$

Table 3. Description of input parameters and probabilities utilized in the quantitative stochastic models for entry assessment of the risk of CDV introduction into wildlife stations through other wild animal infections.

Notation	Definition	Parametrization	Source	Values
**Entry of CDV-infected wild animals**				
A_{pv}	CDV prevalence in other wild animals	Beta (α_1, α_2)	[1]	Beta (5, 8)
A_{opv}	non-CDV prevalence in other wild animals	Beta (α_1, α_2)	-	$1 - A_{pv}$
A_{te}	wild animal was tested CDV screening before entering	PERT (min, most likely, max)	questionnaire	PERT (0.000, 0.026, 0.078)
A_{ote}	wild animal was not tested CDV screening before entering	PERT (min, most likely, max)	-	$1 - A_{te}$
A_{se}	sensitivity of RT-PCR test for CDV	Uniform (min, max)	[9]	Uniform (0.936, 0.962)
A_{ose}	false negative of RT-PCR test for CDV	Uniform (min, max)	-	$1 - A_{se}$
A_{sp}	specificity of RT-PCR test for CDV	Uniform (min, max)	[9]	Uniform (0.75, 0.95)
A_{qu}	wild animals had been quarantined for 30 days	Uniform (min, max)	questionnaire	Uniform (0.950, 1.000)
A_{oqu}	wild animals had not been quarantined for 30 days	Uniform (min, max)	-	$1 - A_{qu}$
A_{sy}	wild animal has CDV symptoms	PERT (min, most likely, max)	[1, 9-11]	PERT (0.320, 0.527, 0.942)
A_{osy}	wild animal has no CDV symptoms	PERT (min, most likely, max)	-	$1 - A_{sy}$
A_{iso}	isolate & treat CDV-symptomatic wild animal	Uniform (min, max)	questionnaire	Uniform (0.950, 1.000)

Notation	Definition	Parametrization	Source	Values
**Entry of CDV-infected wild animals**				
A_c	wild animal carrier after recovery from CDV	Uniform (min, max)	[7-8]	Uniform (0.300, 0.320)
A_{qte}	wild animal was tested CDV with RT-PCR during quarantine	PERT (min, most likely, max)	questionnaire	PERT (0.000, 0.026, 0.078)
A_{oqte}	wild animal was not tested CDV with RT-PCR during quarantine	PERT (min, most likely, max)	-	$1 - A_{qte}$
A_{qse}	sensitivity of RT-PCR test for CDV during quarantine	Uniform (min, max)	[9]	Uniform (0.936, 0.962)
A_{oqse}	false negative of RT-PCR test for CDV during quarantine	Uniform (min, max)	-	$1 - A_{qse}$
A_{qsp}	specificity of RT-PCR test for CDV during quarantine	Uniform (min, max)	[9]	Uniform (0.75, 0.95)
A_{oqsp}	false positive of RT-PCR test for CDV during quarantine	Uniform (min, max)	-	$1 - A_{qsp}$
A_{ve}	CDV vaccine efficacy in civet (America-1 strain)	Beta (α_1, α_2)	[9]	Beta (89, 13)
A_{ove}	CDV vaccine without efficacy in civet	Beta (α_1, α_2)	-	$1 - A_{ve}$
A_{vc}	CDV vaccine coverage in wild animal	Beta (α_1, α_2)	questionnaire	Beta (1, 101)
A_{ovc}	did not give the vaccine in wild animal	Beta (α_1, α_2)	-	$1 - A_{vc}$

Table 4. Description of input parameters and probabilities utilized in the quantitative stochastic models for entry assessment of the risk of CDV introduction into wildlife stations through dog or cat reservoirs and through contaminated hands, animal transport vehicles, and equipment.

Notation	Definition	Parametrization	Source	Values
**Entry of dog or cat reservoirs**				
D_{pv}	CDV prevalence in dogs	Beta (α_1, α_2)	[12]	Beta (5, 99)
D_{opv}	non-CDV prevalence in dogs	Beta (α_1, α_2)	-	$1 - D_{pv}$
D_{ocf}	incomplete fence of the wildlife stations	Beta (α_1, α_2)	questionnaire	Beta (7, 2)
D_{nc}	dogs enter near the tiger cage (≤6 meters)	PERT (min, most likely, max)	questionnaire	PERT (0.000, 0.329, 0.986)
D_{onc}	dogs enter, but far from the tiger cage (>6 meters)	PERT (min, most likely, max)	-	$1 - D_{nc}$
**Entry of CDV-contaminated human hands**				
H_{pv}	CDV prevalence in human hands	Beta (α_1, α_2)	-	$H_{pv} = 1 - [(1 - D_{pv}) * (1 - T_{pv}) * (1 - V_{pv}) * (1 - E_{qpv})]$
H_{opv}	non-CDV prevalence in human hands	Beta (α_1, α_2)	-	$1 - H_{pv}$
H_w	hand washing	Beta (α_1, α_2)	questionnaire	Beta (16, 6)
H_{ow}	no hand washing and percent introduced germs	Beta (α_1, α_2)	-	$1 - H_w$
E_{hw}	effectiveness of hand washing	one value	[13]	0.920
E_{ohw}	ineffectiveness of hand washing	one value	[13]	0.080
H_{cc}	closed-contact humans (veterinarians and keepers)	Uniform (min, max)	questionnaire	Uniform (0.083, 0.182)

Notation	Definition	Parametrization	Source	Values
**Entry of CDV-contaminated animal-transported vehicles**				
V_{pv}	CDV prevalence in vehicle's wheel or mudguard contamination	Beta (α_1, α_2)	$V_{pv} = T_{pv}$	Beta (13, 102)
V_{opv}	non-CDV prevalence in vehicle's wheel or mudguard contamination	Beta (α_1, α_2)	-	$1 - V_{pv}$
V_{cage}	vehicle access to the tiger cage	Beta (α_1, α_2)	questionnaire	Beta (8, 1)
V_{tran}	the rate of transmission of CDV	one value	[14]	0.133
V_{far}	far transportation between wildlife stations	Beta (α1, α2)	questionnaire	Beta (6, 3)
V_{ofar}	not far transportation between wildlife stations	Beta (α1, α2)	-	$1 - V_{far}$
V_{die}	virus die because of far transportation (from temperature and time duration)	one value	[15-16]	0.000
V_d	disinfection of animal-transported vehicles	Beta (α1, α2)	questionnaire	Beta (4, 5)
V_{od}	no disinfection of animal-transported vehicles	Beta (α1, α2)	-	$1 - V_d$
V_{ed}	effective disinfection of the vehicle	one value	[13]	0.920
V_{oed}	ineffective disinfection of the vehicle	one value	[13]	0.080
**Entry of CDV-contaminated equipment**				
E_{qpv}	CDV prevalence of using equipment	Beta (α1, α2)	$E_{qpv} = A_{pv}$	Beta (5, 8)
E_{qopv}	non-CDV prevalence of using equipment	Beta (α1, α2)	-	$1 - E_{qpv}$
E_{qsh}	sharing equipment (e.g., floor cleaning brush, animal	Beta (α1, α2)	questionnaire	Beta (13, 9)

Notation	Definition	Parametrization	Source	Values
	transportation cage) between the tiger and other small wild cat cages			
E_{qosh}	don't sharing equipment between the tiger and other small wild cat cages	Beta (α1, α2)	-	$1 - E_{qsh}$

References for Tables 2-4

1. Suwanpakdee S, Wiratsudakul A, Chaisilp N, Prasittichai L, Skulpong A, Maneeorn P, et al. Canine distemper outbreak and laryngeal paralysis in captive tigers (Panthera tigris). BMC. Forthcoming 2024.

2. Frisk AL, Nig MK, Moritz A, Baumgärtner W. Detection of canine distemper virus nucleoprotein RNA by reverse transcription-PCR using serum, whole blood, and cerebrospinal fluid from dogs with distemper. J Clin Microbiol. 1999: 3634-43.

3. Halecker S, Bock S, Beer M, Hoffmann B. A new molecular detection system for canine distemper virus based on a double-check strategy. Viruses. 2001;13(1632): 1-9.

4. Moritz A, Baumgärtner W, Frisk A-L, König M. Sensitivity and specificity of CDV RT-PCR in the diagnosis of canine distemper. Tierarztl Prax Ausg K Kleintiere Heimtiere. 2003;31(1): 60-6.

5. Nagao Y, Nishio Y, Shiomoda H, Tamaru S, Shimojima M, Goto M, et al. An outbreak of canine distemper virus in tigers (Panthera tigris): possible transmission from wild animals to zoo animals. J Vet Med Sci. 2012;74(6): 699-705.

6. Appel MJG, Yates RA, Foley GL, Bernstein JJ, Santinelli S, Spelman LH, et al. Canine distemper epizootic in lions, tigers, and leopards in North America. Vet Diagn Invest. 1994;6: 277-88.

7. Lanszki Z, Zana B, Zeghbib S, Jakab F, Szabó N, Kemenesi G. Prolonged infection of canine distemper virus in a mixed-breed dog. Vet Sci. 2021;8(61): 1-9.

8. São João T, Machado IC, Diogo RC, Gomes JF, Tavares LM, Gil SA, et al. Characterization of canine distemper clinical presentations during the epidemic outbreak 2014-2018 in Lisbon Metropolitan Area, Portugal. Rev Port Cienc Vet. 2021;116(619): 15-24.

9. Shi N, Zhang L, Yu X, Zhu X, Zhang S, Zhang D, et al. Insight into an outbreak of canine distemper virus infection in Masked Palm civets in China. Front Vet Sci. 2021;8: 1-6.

10. Qui W, Zheng Y, Zhang S, Fan Q, Liu H, Zhang F, et al. Canine distemper outbreak in Rhesus monkeys, China. Emerg Infect Dis. 2011;17(8): 1541-3.

11. Van de Bildt MWG, Kuiken T, Visee AM, Lema S, Fitzjohn TR, Osterhaus A DME. Distemper outbreak and its effect on African wild dog conservation. Emerg Infect Dis. 2002;8(2): 211-3.

12. Posuwan N, Payungporn S, Thontiravong A, Kitikoon P, Amonsin A, Poovorawan Y. Prevalence of respiratory viruses isolated from dogs in Thailand during 2008-2009. Asian Biomed. 2010;4(4): 563-9.

13. Burton M, Cobb E, Donachie P, Judah G, Curtis V, Schmidt W-P. The effect of handwashing with water or soap on bacterial contamination of hands. Int J Environ Res Public Health. 2011;8: 97-104.

14. Nouvellet P, Donnelly CA, Nardi MD, Rhodes CJ, Benedictis PD, Citterio, et al. Rabies and canine distemper virus epidemics in the red fox population of Northern Italy (2006-2010). PLoS ONE. 2013;8(4):e61588..

15. Ho CK and Babiuk LA. A new plaque system for canine distemper: characteristics of the green strain of canine distemper virus. Can J Microbiol. 1979;25(6): 680-5.

16. Climate and average weather in Thailand. World Weather & Climate Information [Internet]. 2010-2023 [cited 2023 June 25]. Available from: https://weather-and-climate.com/average-monthly-Rainfall-Temperature-Sunshine-in-Thailand.

Supporting information

S7 Table 5

Notations	Definitions	Parameter estimations		
		5th percentile	Median	95th percentile
**Entry of CDV-infected tigers**				
T_{pv}	CDV prevalence in tigers	0.0697	0.1106	0.1640
T_{opv}	non-CDV prevalence in tigers	0.8360	0.8894	0.9303
T_{te}	tiger was tested CDV screening before entering	0.0515	0.1570	0.3161
T_{ote}	tiger was not tested CDV screening before entering	0.6839	0.8430	0.9485
T_{se}	sensitivity of RT-PCR test for CDV	0.8269	0.9117	0.9914
T_{ose}	false negative of RT-PCR test for CDV	0.0086	0.0883	0.1731
T_{sp}	specificity of RT-PCR test for CDV	0.8584	0.9222	0.9936
T_{qu}	tiger had been quarantined for 30 days	0.9528	0.9748	0.9975
T_{oqu}	tiger had not been quarantined for 30 days	0.0025	0.0252	0.0472
T_{sy}	tiger has CDV symptoms	0.4761	0.5093	0.5420
T_{osy}	tiger has no CDV symptoms	0.4580	0.4907	0.5239
T_{iso}	isolate & treat CDV-symptomatic tiger	0.9521	0.9733	0.9963
T_{c}	tiger carrier after recovery from CDV	0.3038	0.3343	0.3665
T_{qte}	tiger was tested CDV with RT-PCR during quarantine	0.0488	0.1544	0.3051
T_{oqte}	tiger was not tested CDV with RT-PCR during quarantine	0.6949	0.8456	0.9512
T_{qse}	sensitivity of RT-PCR test for CDV during quarantine	0.8275	0.9090	0.9896
T_{oqse}	false negative of RT-PCR test for CDV during quarantine	0.0104	0.0910	0.1725
T_{qsp}	specificity of RT-PCR test for CDV during quarantine	0.8602	0.9229	0.9908
T_{oqsp}	false positive of RT-PCR test for CDV during quarantine	0.0092	0.0771	0.1398
T_{ve}	CDV vaccine efficacy in tiger (Recombinant vaccine)	0.1451	0.2034	0.2764
T_{ove}	CDV vaccine without efficacy in tiger	0.7236	0.7966	0.8549
T_{vc}	CDV vaccine coverage in tiger	0.0484	0.0780	0.1139
T_{ovc}	did not give the vaccine in tiger	0.8861	0.9220	0.9516
**Entry of CDV-infected wild animals**				

Notations	Definitions	Parameter estimations		
		5th percentile	Median	95th percentile
A_{pv}	CDV prevalence in other wild animals	0.1809	0.3759	0.6218
A_{opv}	non-CDV prevalence in other wild animals	0.3782	0.6241	0.8191
A_{te}	wild animal was tested CDV screening before entering	0.0093	0.0287	0.0558
A_{ote}	wild animal was not tested CDV screening before entering	0.9442	0.9713	0.9907
A_{se}	sensitivity of RT-PCR test for CDV	0.9367	0.9479	0.9599
A_{ose}	false negative of RT-PCR test for CDV	0.0401	0.0521	0.0633
A_{sp}	specificity of RT-PCR test for CDV	0.7600	0.8493	0.9374
A_{qu}	wild animals had been quarantined for 30 days	0.9530	0.9747	0.9976
A_{oqu}	wild animals had not been quarantined for 30 days	0.0024	0.0253	0.0470
A_{sy}	wild animal has CDV symptoms	0.3859	0.5506	0.7654
A_{osy}	wild animal has no CDV symptoms	0.2346	0.4494	0.6141
A_{iso}	isolate & treat CDV-symptomatic wild animal	0.9525	0.9758	0.9975
A_{c}	wild animal carrier after recovery from CDV	0.3013	0.3104	0.3189
A_{qte}	wild animal was tested CDV with RT-PCR during quarantine	0.0088	0.0310	0.0568
A_{oqte}	wild animal was not tested CDV with RT-PCR during quarantine	0.9432	0.9690	0.9912
A_{qse}	sensitivity of RT-PCR test for CDV during quarantine	0.9371	0.9485	0.9604
A_{oqse}	false negative of RT-PCR test for CDV during quarantine	0.0396	0.0515	0.0629
A_{qsp}	specificity of RT-PCR test for CDV during quarantine	0.7599	0.8514	0.9408
A_{oqsp}	false positive of RT-PCR test for CDV during quarantine	0.0592	0.1486	0.2401
A_{ve}	CDV vaccine efficacy in civet (America-1 strain)	0.8180	0.8751	0.9221
A_{ove}	CDV vaccine without efficacy in civet	0.0779	0.1249	0.1820
A_{vc}	CDV vaccine coverage in wild animal	0.0006	0.0067	0.0289
A_{ovc}	did not give the vaccine in wild animal	0.9711	0.9933	0.9994
**Entry of dog or cat reservoirs**				
D_{pv}	CDV prevalence in dogs	0.0178	0.0453	0.0895
D_{opv}	non-CDV prevalence in dogs	0.9105	0.9547	0.9822
D_{ocf}	incomplete fence of the wildlife stations	0.5339	0.8073	0.9536
D_{nc}	dogs enter near the tiger cage (≤6 meters)	0.0970	0.3564	0.6963
D_{onc}	dogs enter, but far from the tiger cage (>6 meters)	0.3037	0.6436	0.9030

Notations	Definitions	Parameter estimations		
		5th percentile	Median	95th percentile
**Entry of CDV-contaminated human hands**				
H_{pv}	CDV prevalence in human hands	0.3721	0.5342	0.7194
H_{opv}	non-CDV prevalence in human hands	0.2806	0.4658	0.6279
H_{w}	hand washing	0.5578	0.7382	0.8695
H_{ow}	no hand washing and percent introduced germs	0.1305	0.2618	0.4422
H_{cc}	closed-contact humans (veterinarians and keepers)	0.0885	0.1346	0.1766
**Entry of CDV-contaminated animal-transported vehicles**				
V_{pv}	CDV prevalence in vehicle’s wheel or mudguard contamination	0.0682	0.1106	0.1634
V_{opv}	non-CDV prevalence in vehicle’s wheel or mudguard contamination	0.8366	0.8894	0.9318
V_{cage}	vehicle access to the tiger cage	0.6595	0.9176	0.9923
V_{far}	far transportation between wildlife stations	0.4116	0.6885	0.8949
V_{ofar}	not far transportation between wildlife stations	0.1051	0.3115	0.5884
V_{d}	disinfection of animal-transported vehicles	0.1930	0.4324	0.7040
V_{od}	no disinfection of animal-transported vehicles	0.2960	0.5676	0.8070
**Entry of CDV-contaminated equipment**				
E_{qpv}	CDV prevalence of using equipment	0.1960	0.3791	0.6191
E_{qopv}	non-CDV prevalence of using equipment	0.3809	0.6209	0.8040
E_{qsh}	sharing equipment (e.g., floor cleaning brush, animal transportation cage) between the tiger and other small wild cat cages	0.4161	0.5973	0.7483
E_{qosh}	don’t sharing equipment between the tiger and other small wild cat cages	0.2491	0.4078	0.5853

Supporting information

Fig S8

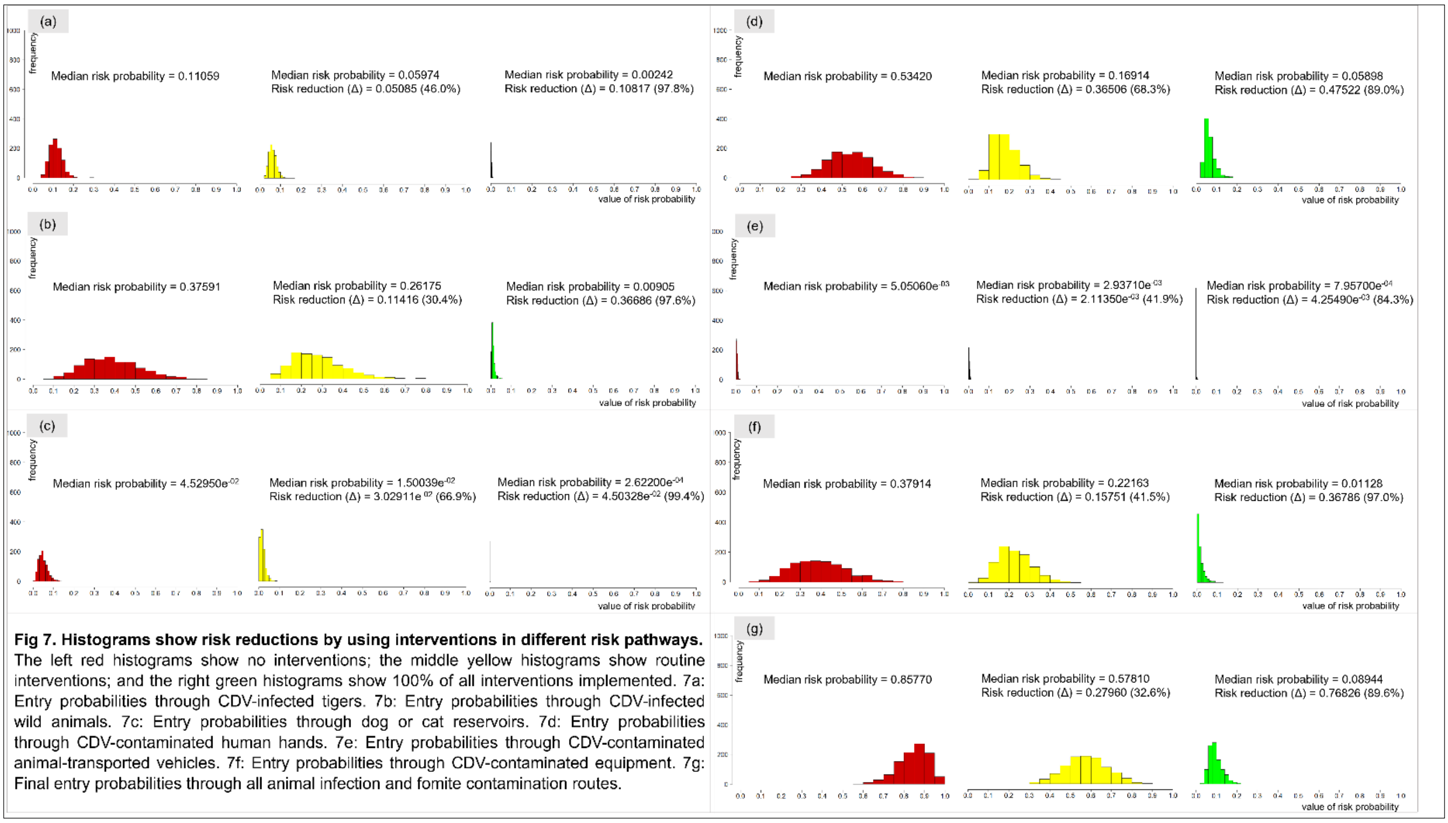

Fig 7. Histograms show risk reductions by using interventions in different risk pathways. The left red histograms show no interventions; the middle yellow histograms show routine interventions; and the right green histograms show 100% of all interventions implemented. 7a: Entry probabilities through CDV-infected tigers. 7b: Entry probabilities through CDV-infected wild animals. 7c: Entry probabilities through dog or cat reservoirs. 7d: Entry probabilities through CDV-contaminated human hands. 7e: Entry probabilities through CDV-contaminated animal-transported vehicles. 7f: Entry probabilities through CDV-contaminated equipment. 7g: Final entry probabilities through all animal infection and fomite contamination routes.

Supporting information

S9 Questionnaires QN1 and QN2

Questionnaire for Research Project (For Station Directors or Veterinarians)

Subject: Quantitative Risk Assessment of Canine Distemper Virus Infection Through Humans, Reservoir Hosts, and Vehicles in Tigers at Wildlife Stations Conducting Wildlife Activities in Thailand

Instructions

This questionnaire is divided into 7 sections:

Sections 1-2: Answer all questions.

Sections 3-7: Answer only the sections relevant to your agency (based on the specific animal species present at your station). If a specific animal group is not present, skip to the next section.

Section 1: General Information

1.1. Is your wildlife station located near a community area?

☐ Located far from a community area

☐ Located near a community area, approximately .... meters/kilometers away

1.2. Does your wildlife station involve wildlife transportation?

☐ No wildlife transportation at all

☐ Wildlife transportation occurs, originating from:

○ Transport from other wildlife stations under the DNP

- Year 2016: Transported from ....
- Year 2017: Transported from ....

- Year 2018: Transported from ....
- Year 2019: Transported from ....
- Year 2020: Transported from ....
- Year 2021: Transported from ....

○ Transport to other wildlife stations under the DNP

- Year 2016: Transported to ....
- Year 2017: Transported to ....
- Year 2018: Transported to ....
- Year 2019: Transported to ....
- Year 2020: Transported to ....
- Year 2021: Transported to ....

○ Transport from other locations (e.g., private sector, animal owners, etc.):

- Year 2016: Transported from ....
- Year 2017: Transported from ....
- Year 2018: Transported from ....
- Year 2019: Transported from ....
- Year 2020: Transported from ....
- Year 2021: Transported from ....

1.3. Does your wildlife station allow external individuals to enter?

☐ No, external individuals are not allowed to enter wildlife station.

☐ Yes, external individuals are allowed to enter wildlife station.

1.3.1. Where do these external individuals come from?

- ○ Officials from the DNP but not staff of your wildlife station

  (On average per month, they enter the station approximately .... times)

- ○ Animal keepers from other locations not under the DNP

  (On average per month, they enter the station approximately .... times)

- ○ Personnel from other agencies visit for observation, study tours, or official matters

  (On average per month, they enter the station approximately .... times)

- ○ Internship students

  (On average per month, they enter the station approximately .... times)

- ○ General public

  (On average per month, they enter the station approximately .... times)

- ○ Others (please specify): ....

  (On average per month, they enter the station approximately .... times)

1.3.2. Which areas of wildlife station are external individuals permitted to enter?

- ○ Wildlife exhibition areas for tourist visits - Who is permitted to enter?
  - ○ Officials from the DNP, but not staff of your wildlife station
  - ○ Animal keepers from other locations not under the DNP
  - ○ Personnel from other agencies visit for observation, study tours, or official matters
  - ○ Internship students
  - ○ General public

- ◯ Others, please specify: ....

◯ Animal quarantine areas - Who is permitted to enter?

- ◯ Officials from the DNP, but not staff of your wildlife station
- ◯ Animal keepers from other locations not under the DNP
- ◯ Personnel from other agencies visit for observation, study tours, or official matters
- ◯ Internship students
- ◯ General public
- ◯ Others, please specify: ....

◯ Animal enclosure areas - Who is permitted to enter?

- ◯ Officials from the DNP, but not staff of your wildlife station
- ◯ Animal care staff from other locations not under the DNP
- ◯ Personnel from other agencies visit for observation, study tours, or official matters
- ◯ Internship students
- ◯ General public
- ◯ Others, please specify: ....

◯ Central office of wildlife stations - Who is permitted to enter?

- ◯ Officials from the DNP, but not staff of your wildlife station

- ○ Animal care staff from other locations not under the DNP
- ○ Personnel from other agencies visit for observation, study tours, or official matters
- ○ Internship students
- ○ General public
- ○ Others, please specify: ....

○ Other areas, please specify .... - Who is permitted to enter?

- ○ Officials from the DNP, but not staff of your wildlife station
- ○ Animal care staff from other locations not under the DNP
- ○ Personnel from other agencies visit for observation, study tours, or official matters
- ○ Internship students
- ○ General public
- ○ Others, please specify: ....

1.4. By what methods has your wildlife station acquired wildlife?

- ☐ Obtained as evidence in wildlife-related illegal cases
- ☐ Donated by individuals who previously owned wildlife
- ☐ Transported from other wildlife stations
- ☐ Acquired through other means, please specify: ....

1.5. Besides tigers, does your wildlife station house other wildlife species?

☐ No other wildlife species

☐ Yes, other wildlife species include:

- ○ Other species in the other wild cat group, please specify: .... (Answer in Section 3, Page 10)
- ○ Species in the wild dog group, please specify: .... (Answer in Section 4, Page 14)
- ○ Species in the civet group, please specify: .... (Answer in Section 5, Page 17)
- ○ Species in the bear group, please specify: .... (Answer in Section 6, Page 21)
- ○ Species in the monkey group, please specify: .... (Answer in Section 7, Page 24)
- ○ Other species, please specify: ....

Section 2: Tigers

2.1. Number of tigers (2016-2021)

Number of tigers categorized by type (individuals)	2016	2017	2018	2019	2020	2021
Number of tigers obtained as evidence in illegal cases combine with number of tigers received through donations						
Number of tigers transported from other wildlife stations						
Number of tigers transported to other wildlife stations						
Number of tigers originally present at the station						
**Total number of tigers**						

2.2. Number of tigers obtained as evidence in legal cases and through donations (individuals)

Methods of CDV testing before entering wildlife station, test results, and vaccination status		2016	2017	2018	2019	2020	2021
CDV testing by ELISA Kit	Positive result						
	Negative result						
CDV testing by RT-PCR	Positive result						
	Negative result						
CDV testing with negative results	Received CDV vaccine						
	Did not receive CDV vaccine						
No CDV testing conducted	Received CDV vaccine						
	Did not receive CDV vaccine						

2.3. Number of tigers transported to wildlife station from other stations (individuals)

Methods of CDV testing before entering wildlife station, test results, and vaccination status		2016	2017	2018	2019	2020	2021
CDV testing by ELISA Kit	Positive result						
	Negative result						
CDV testing by RT-PCR	Positive result						
	Negative result						
CDV testing with negative results	Received CDV vaccine						
	Did not receive CDV vaccine						
No CDV testing conducted	Received CDV vaccine						
	Did not receive CDV vaccine						

2.4. Number of existing tigers (individuals)

Methods of CDV testing before entering wildlife station, test results, and vaccination status		2016	2017	2018	2019	2020	2021
CDV testing by ELISA Kit	Positive result						
	Negative result						
CDV testing by RT-PCR	Positive result						
	Negative result						
CDV testing with negative results	Received CDV vaccine						
	Did not receive CDV vaccine						
No CDV testing conducted	Received CDV vaccine						
	Did not receive CDV vaccine						

2.5. Types of vaccines administered to tigers

Types of Vaccines (Specify brand & trade name)	Animal species	Vaccination Details	Remarks (Upload vaccine photos)
○ MSD/Nobivac®		Vaccinated: .... individuals<br>Out of a total of: .... individuals	
○ MSD/Rabisin®		Vaccinated: .... individuals<br>Out of a total of: .... individuals	
○ Zoetis/Felocell®		Vaccinated: .... individuals<br>Out of a total of: .... individuals	
○ Zoetis/Defensor®		Vaccinated: .... individuals<br>Out of a total of: .... individuals	
○ Zoetis/Vanguard®		Vaccinated: .... individuals<br>Out of a total of: .... individuals	
○ Bayer/Bayovac L®		Vaccinated: .... individuals<br>Out of a total of: .... individuals	
○ Bayer/Bayovac R®		Vaccinated: .... individuals<br>Out of a total of: .... individuals	
○ Boehringer/Recombitek R®		Vaccinated: .... individuals<br>Out of a total of: .... individuals	
○ Others, please specify<br>...<br>...		Vaccinated: .... individuals<br>Out of a total of: .... individuals	

Section 3: Other wild cat species, including leopards/black panthers, clouded leopards, golden cats, fishing cats, flat-headed cats, marbled cats, Asian leopard cats, and jungle cats

3.1 Does your wildlife station have other wild cat species, excluding tiger?

If so, what species?

☐ No other tiger species

☐ Yes, there are other tiger species (please specify the number of individuals in 2021):

- ○ Leopard: .... / Black panther: ....
- ○ Clouded leopard: ....
- ○ Golden cat: ....
- ○ Fishing cat: ....
- ○ Flat-headed cat: ....
- ○ Marbled cat: ....
- ○ Asian leopard cat: ....
- ○ Jungle cat: ....

3.2 Total number of all other species in the wild cat group (excluding tigers) during 2016-2021

**Total number of other wild cats categorized by type (individuals)**	**2016**	**2017**	**2018**	**2019**	**2020**	**2021**
Number of wild cats obtained as evidence in illegal cases combine with number of wild cats received through donations						
Number of wild cats transported from other wildlife stations						
Number of wild cats transported to other wildlife stations						
Number of wild cats originally present at the station						
**Total number of other wild cats**						

3.3 The total number of other wild cat species that were confiscated as case evidence, including the number of wild cats received as donations (individuals).

Methods of CDV testing before entering wildlife station, test results, and vaccination status		**2016**	**2017**	**2018**	**2019**	**2020**	**2021**
CDV testing by ELISA Kit	Positive result						
	Negative result						
CDV testing by RT-PCR	Positive result						
	Negative result						
CDV testing with negative results	Received CDV vaccine						
	Did not receive CDV vaccine						
No CDV testing conducted	Received CDV vaccine						
	Did not receive CDV vaccine						

3.4 The number of other wild cat species that have been transported from other stations (individuals).

Methods of CDV testing before entering wildlife station, test results, and vaccination status		**2016**	**2017**	**2018**	**2019**	**2020**	**2021**
CDV testing by ELISA Kit	Positive result						
	Negative result						
CDV testing by RT-PCR	Positive result						
	Negative result						
CDV testing with negative results	Received CDV vaccine						
	Did not receive CDV vaccine						
No CDV testing conducted	Received CDV vaccine						
	Did not receive CDV vaccine						

3.5. The number of other wild cat species that were originally present (individuals).

Methods of CDV testing before entering wildlife station, test results, and vaccination status		2016	2017	2018	2019	2020	2021
CDV testing by ELISA Kit	Positive result						
	Negative result						
CDV testing by RT-PCR	Positive result						
	Negative result						
CDV testing with negative results	Received CDV vaccine						
	Did not receive CDV vaccine						
No CDV testing conducted	Received CDV vaccine						
	Did not receive CDV vaccine						

3.6. Types of vaccines administered to other wild cat species

Types of Vaccines (Specify brand & trade name)	Animal species	Vaccination Details	Remarks (Upload vaccine photos)
○ MSD/Nobivac®		Vaccinated: .... individuals<br>Out of a total of: .... individuals	
○ MSD/Rabisin®		Vaccinated: .... individuals<br>Out of a total of: .... individuals	
○ Zoetis/Felocell®		Vaccinated: .... individuals<br>Out of a total of: .... individuals	
○ Zoetis/Defensor®		Vaccinated: .... individuals<br>Out of a total of: .... individuals	
○ Zoetis/Vanguard®		Vaccinated: .... individuals<br>Out of a total of: .... individuals	

Types of Vaccines (Specify brand & trade name)	Animal species	Vaccination Details	Remarks (Upload vaccine photos)
◯ Bayer/Bayovac L®		Vaccinated: .... individuals<br>Out of a total of: .... individuals	
◯ Bayer/Bayovac R®		Vaccinated: .... individuals<br>Out of a total of: .... individuals	
◯ Boehringer/Recombitek R®		Vaccinated: .... individuals<br>Out of a total of: .... individuals	
◯ Others, please specify<br>..<br>..		Vaccinated: .... individuals<br>Out of a total of: .... individuals	

Section 4: Wild dog species

4.1 What species in the wild dog group are present at your wildlife station? (Please specify the number of individuals in 2021)

- ☐ Asiatic Jackal ....
- ☐ Dhole ....
- ☐ Others (please specify) ....

4.2 Number of wild dogs during the period 2016–2021

**Total number of wild dogs categorized by type (individuals)**	**2016**	**2017**	**2018**	**2019**	**2020**	**2021**
Number of wild dogs obtained as evidence in illegal cases combine with number of wild dogs received through donations						
Number of wild dogs transported from other wildlife stations						
Number of wild dogs transported to other wildlife stations						
Number of wild dogs originally present at the station						
**Total number of wild dogs**						

4.3 The total number of wild dogs that were confiscated as case evidence, including the number of wild dogs received as donations (individuals)

Methods of CDV testing before entering wildlife station, test results, and vaccination status		**2016**	**2017**	**2018**	**2019**	**2020**	**2021**
CDV testing by ELISA Kit	Positive result						
	Negative result						
CDV testing by RT-PCR	Positive result						
	Negative result						
CDV testing with negative results	Received CDV vaccine						
	Did not receive CDV vaccine						

Methods of CDV testing before entering wildlife station, test results, and vaccination status		2016	2017	2018	2019	2020	2021
No CDV testing conducted	Received CDV vaccine						
	Did not receive CDV vaccine						

4.4 The number of wild dogs that have been transported from other stations (individuals)

Methods of CDV testing before entering wildlife station, test results, and vaccination status		2016	2017	2018	2019	2020	2021
CDV testing by ELISA Kit	Positive result						
	Negative result						
CDV testing by RT-PCR	Positive result						
	Negative result						
CDV testing with negative results	Received CDV vaccine						
	Did not receive CDV vaccine						
No CDV testing conducted	Received CDV vaccine						
	Did not receive CDV vaccine						

4.5. The number of wild dogs that were originally present (individuals)

Methods of CDV testing before entering wildlife station, test results, and vaccination status		2016	2017	2018	2019	2020	2021
CDV testing by ELISA Kit	Positive result						
	Negative result						
CDV testing by RT-PCR	Positive result						
	Negative result						
CDV testing with negative results	Received CDV vaccine						
	Did not receive CDV vaccine						
No CDV testing conducted	Received CDV vaccine						
	Did not receive CDV vaccine						

4.6. Types of vaccines administered to wild dogs

Questionnaire Number: QN01 Date of Information Provided: ....

Wildlife Station Code: ....

Types of Vaccines (Specify brand & trade name)	Animal species	Vaccination Details	Remarks (Upload vaccine photos)
○ MSD/Nobivac®		Vaccinated: .... individuals<br>Out of a total of: .... individuals	
○ MSD/Rabisin®		Vaccinated: .... individuals<br>Out of a total of: .... individuals	
○ Zoetis/Felocell®		Vaccinated: .... individuals<br>Out of a total of: .... individuals	
○ Zoetis/Defensor®		Vaccinated: .... individuals<br>Out of a total of: .... individuals	
○ Zoetis/Vanguard®		Vaccinated: .... individuals<br>Out of a total of: .... individuals	
○ Bayer/Bayovac L®		Vaccinated: .... individuals<br>Out of a total of: .... individuals	
○ Bayer/Bayovac R®		Vaccinated: .... individuals<br>Out of a total of: .... individuals	
○ Boehringer/Recombitek R®		Vaccinated: .... individuals<br>Out of a total of: .... individuals	
○ Others, please specify<br>..<br>..		Vaccinated: .... individuals<br>Out of a total of: .... individuals	

Section 5: Civet species

5.1 What species in the civet group are present at your wildlife station? (Please specify the number of individuals in 2021)

- ○ Small Indian civet ....
- ○ Large Indian civet ....
- ○ Otter civet ....
- ○ Masked palm civet ....
- ○ Small-toothed palm civet ....
- ○ Binturong ....
- ○ Common palm civet ....
- ○ Large-spotted Civet ....
- ○ Prionodon linsang ....
- ○ Spotted Linsang ....
- ○ Banded Palm Civet ....
- ○ Others, please specify ....

5.2 Number of civets during the period 2016–2021

**Total number of civets categorized by type (individuals)**	**2016**	**2017**	**2018**	**2019**	**2020**	**2021**
Number of civets obtained as evidence in illegal cases combine with number of civets received through donations						
Number of civets transported from other wildlife stations						
Number of civets transported to other wildlife stations						
Number of civets originally present at the station						
**Total number of civets**						

5.3 The total number of civets that were confiscated as case evidence, including the number of civets received as donations (individuals)

Methods of CDV testing before entering wildlife station, test results, and vaccination status		2016	2017	2018	2019	2020	2021
CDV testing by ELISA Kit	Positive result						
	Negative result						
CDV testing by RT-PCR	Positive result						
	Negative result						
CDV testing with negative results	Received CDV vaccine						
	Did not receive CDV vaccine						
No CDV testing conducted	Received CDV vaccine						
	Did not receive CDV vaccine						

5.4 The number of civets that have been transported from other stations (individuals)

Methods of CDV testing before entering wildlife station, test results, and vaccination status		2016	2017	2018	2019	2020	2021
CDV testing by ELISA Kit	Positive result						
	Negative result						
CDV testing by RT-PCR	Positive result						
	Negative result						
CDV testing with negative results	Received CDV vaccine						
	Did not receive CDV vaccine						
No CDV testing conducted	Received CDV vaccine						
	Did not receive CDV vaccine						

5.5. The number of civets that were originally present (individuals)

Methods of CDV testing before entering wildlife station, test results, and vaccination status		2016	2017	2018	2019	2020	2021
CDV testing by ELISA Kit	Positive result						

Methods of CDV testing before entering wildlife station, test results, and vaccination status		2016	2017	2018	2019	2020	2021
	Negative result						
CDV testing by RT-PCR	Positive result						
	Negative result						
CDV testing with negative results	Received CDV vaccine						
	Did not receive CDV vaccine						
No CDV testing conducted	Received CDV vaccine						
	Did not receive CDV vaccine						

5.6. Types of vaccines administered to civets

Types of Vaccines (Specify brand & trade name)	Animal species	Vaccination Details	Remarks (Upload vaccine photos)
○ MSD/Nobivac®		Vaccinated: .... individuals<br>Out of a total of: .... individuals	
○ MSD/Rabisin®		Vaccinated: .... individuals<br>Out of a total of: .... individuals	
○ Zoetis/Felocell®		Vaccinated: .... individuals<br>Out of a total of: .... individuals	
○ Zoetis/Defensor®		Vaccinated: .... individuals<br>Out of a total of: .... individuals	
○ Zoetis/Vanguard®		Vaccinated: .... individuals<br>Out of a total of: .... individuals	
○ Bayer/Bayovac L®		Vaccinated: .... individuals<br>Out of a total of: .... individuals	

Types of Vaccines (Specify brand & trade name)	Animal species	Vaccination Details	Remarks (Upload vaccine photos)
○ Bayer/Bayovac R®		Vaccinated: .... individuals<br>Out of a total of: .... individuals	
○ Boehringer/Recombitek R®		Vaccinated: .... individuals<br>Out of a total of: .... individuals	
○ Others, please specify<br>..<br>..		Vaccinated: .... individuals<br>Out of a total of: .... individuals	

Section 6: Bears

6.1 What species in the bear group are present at your wildlife station? (Please specify the number of individuals in 2021)

- ○ Asiatic black bear .... ○ Malayan sun bear ....
- ○ Others, please specify ....

6.2 Number of bears during the period 2016–2021

**Total number of bears categorized by type (individuals)**	**2016**	**2017**	**2018**	**2019**	**2020**	**2021**
Number of bears obtained as evidence in illegal cases combine with number of bears received through donations						
Number of bears transported from other wildlife stations						
Number of bears transported to other wildlife stations						
Number of bears originally present at the station						
**Total number of bears**						

6.3 The total number of bears that were confiscated as case evidence, including the number of bears received as donations (individuals)

Methods of CDV testing before entering wildlife station, test results, and vaccination status		**2016**	**2017**	**2018**	**2019**	**2020**	**2021**
CDV testing by ELISA Kit	Positive result						
	Negative result						
CDV testing by RT-PCR	Positive result						
	Negative result						
CDV testing with negative results	Received CDV vaccine						
	Did not receive CDV vaccine						
	Received CDV vaccine						

Methods of CDV testing before entering wildlife station, test results, and vaccination status		2016	2017	2018	2019	2020	2021
No CDV testing conducted	Did not receive CDV vaccine						

6.4 The number of bears that have been transported from other stations (individuals)

Methods of CDV testing before entering wildlife station, test results, and vaccination status		2016	2017	2018	2019	2020	2021
CDV testing by ELISA Kit	Positive result						
	Negative result						
CDV testing by RT-PCR	Positive result						
	Negative result						
CDV testing with negative results	Received CDV vaccine						
	Did not receive CDV vaccine						
No CDV testing conducted	Received CDV vaccine						
	Did not receive CDV vaccine						

6.5. The number of bears that were originally present (individuals)

Methods of CDV testing before entering wildlife station, test results, and vaccination status		2016	2017	2018	2019	2020	2021
CDV testing by ELISA Kit	Positive result						
	Negative result						
CDV testing by RT-PCR	Positive result						
	Negative result						
CDV testing with negative results	Received CDV vaccine						
	Did not receive CDV vaccine						
No CDV testing conducted	Received CDV vaccine						
	Did not receive CDV vaccine						

6.6. Types of vaccines administered to bears

Types of Vaccines (Specify brand & trade name)	Animal species	Vaccination Details	Remarks (Upload vaccine photos)
○ MSD/Nobivac®		Vaccinated: .... individuals<br>Out of a total of: .... individuals	
○ MSD/Rabisin®		Vaccinated: .... individuals<br>Out of a total of: .... individuals	
○ Zoetis/Felocell®		Vaccinated: .... individuals<br>Out of a total of: .... individuals	
○ Zoetis/Defensor®		Vaccinated: .... individuals<br>Out of a total of: .... individuals	
○ Zoetis/Vanguard®		Vaccinated: .... individuals<br>Out of a total of: .... individuals	
○ Bayer/Bayovac L®		Vaccinated: .... individuals<br>Out of a total of: .... individuals	
○ Bayer/Bayovac R®		Vaccinated: .... individuals<br>Out of a total of: .... individuals	
○ Boehringer/Recombitek R®		Vaccinated: .... individuals<br>Out of a total of: .... individuals	
○ Others, please specify<br>...<br>...		Vaccinated: .... individuals<br>Out of a total of: .... individuals	

Section 7: Monkeys

7.1 What species in the monkey group are present at your wildlife station? (Please specify the number of individuals in 2021)

- ○ Rhesus macaque ....
- ○ Crab-eating macaque ....
- ○ Northern pig-tailed macaque ....
- ○ Southern pig-tailed macaque ....
- ○ Assamese macaque ....
- ○ Stump-tailed macaque ....
- ○ Others, please specify ....

7.2 Number of monkeys during the period 2016–2021

**Total number of monkeys categorized by type (individuals)**	**2016**	**2017**	**2018**	**2019**	**2020**	**2021**
Number of monkeys obtained as evidence in illegal cases combine with number of monkeys received through donations						
Number of monkeys transported from other wildlife stations						
Number of monkeys transported to other wildlife stations						
Number of monkeys originally present at the station						
**Total number of monkeys**						

7.3 The total number of monkeys that were confiscated as case evidence, including the number of monkeys received as donations (individuals)

Methods of CDV testing before entering wildlife station, test results, and vaccination status		**2016**	**2017**	**2018**	**2019**	**2020**	**2021**
CDV testing by ELISA Kit	Positive result						
	Negative result						
CDV testing by RT-PCR	Positive result						
	Negative result						
	Received CDV vaccine						

Methods of CDV testing before entering wildlife station, test results, and vaccination status		2016	2017	2018	2019	2020	2021
CDV testing with negative results	Did not receive CDV vaccine						
No CDV testing conducted	Received CDV vaccine						
	Did not receive CDV vaccine						

7.4 The number of monkeys that have been transported from other stations (individuals)

Methods of CDV testing before entering wildlife station, test results, and vaccination status		2016	2017	2018	2019	2020	2021
CDV testing by ELISA Kit	Positive result						
	Negative result						
CDV testing by RT-PCR	Positive result						
	Negative result						
CDV testing with negative results	Received CDV vaccine						
	Did not receive CDV vaccine						
No CDV testing conducted	Received CDV vaccine						
	Did not receive CDV vaccine						

7.5. The number of monkeys that were originally present (individuals)

Methods of CDV testing before entering wildlife station, test results, and vaccination status		2016	2017	2018	2019	2020	2021
CDV testing by ELISA Kit	Positive result						
	Negative result						
CDV testing by RT-PCR	Positive result						
	Negative result						
CDV testing with negative results	Received CDV vaccine						
	Did not receive CDV vaccine						
	Received CDV vaccine						

Methods of CDV testing before entering wildlife station, test results, and vaccination status		**2016**	**2017**	**2018**	**2019**	**2020**	**2021**
No CDV testing conducted	Did not receive CDV vaccine						

7.6. Types of vaccines administered to monkeys

Types of Vaccines (Specify brand & trade name)	Animal species	Vaccination Details	Remarks (Upload vaccine photos)
○ MSD/Nobivac®		Vaccinated: .... individuals<br>Out of a total of: .... individuals	
○ MSD/Rabisin®		Vaccinated: .... individuals<br>Out of a total of: .... individuals	
○ Zoetis/Felocell®		Vaccinated: .... individuals<br>Out of a total of: .... individuals	
○ Zoetis/Defensor®		Vaccinated: .... individuals<br>Out of a total of: .... individuals	
○ Zoetis/Vanguard®		Vaccinated: .... individuals<br>Out of a total of: .... individuals	
○ Bayer/Bayovac L®		Vaccinated: .... individuals<br>Out of a total of: .... individuals	
○ Bayer/Bayovac R®		Vaccinated: .... individuals<br>Out of a total of: .... individuals	
○ Boehringer/Recombitek R®		Vaccinated: .... individuals<br>Out of a total of: .... individuals	
○ Others, please specify<br>..		Vaccinated: .... individuals<br>Out of a total of: .... individuals	

Questionnaire Number: QN02 Date of Information Provided: ....

Wildlife Station Code: ....

Questionnaire for Research Project (For Wild Animal Keepers of Wildlife Stations)

Subject: Quantitative Risk Assessment of Canine Distemper Virus Infection Through Humans, Reservoir Hosts, and Vehicles in Tigers at Wildlife Stations Conducting Wildlife Activities in Thailand

Instructions<br>This questionnaire is divided into two sections:<br>Section 1 pertains to the risk of contact with canine distemper virus prior to wild animals being brought into wildlife station. Section 2 pertains to the risk of contact with canine distemper virus within wildlife station, including the animal quarantine and enclosure areas.<br>Please respond to all the questions in the questionnaire as follows.

Section 1: Risk of contact with canine distemper virus before being brought into wildlife station

1.1 Information of domestic dogs around the wildlife breeding station / wildlife rescue center

1.1.1. Have domestic dogs been regularly seen around the station?

☐ No sightings (Skip to question 1.1.3) ☐ Sighted (Proceed to question 1.1 .2)

1.1.2. If domestic dogs have been seen around the station, were they present throughout the year?

1.1.2.1. If yes, approximately how many months per year are domestic dogs seen around the station? .... months

1.1.2.2. In one month, how many times are domestic dogs sighted, and how many dogs are seen?

Number of sightings: .... times, Number of dogs: ....

1.1.3. Have any domestic dogs infected with canine distemper been seen in the area around the wildlife breeding station?

☐ Never seen ☐ Seen; approximately .... times/month

1.1.4. Can dogs regularly enter the station? If yes, through which routes?

....

....

....

....

....

....

1.2. Information of domestic dogs around the private residences of wildlife station staff

1.2.1. Are the private residences of the staff located within the station premises?

☐ No, the residences are outside the station, approximately .... meters/kilometers away.

☐ Yes, the residences are within the station premises.

1.2.2. In the area around the private residences of the staff:

Number of dogs kept: .... Number of cats kept: ....

1.2.3. Have any dogs infected with canine distemper been seen in the area around the private residences of the staff?

☐ Never seen ☐ Seen; approximately .... times/month

1.3. Information of individuals who have direct contact with tigers or have access to the tiger enclosure area within the wildlife breeding station / wildlife rescue center

**1.3.1. Duties of the staff at the wildlife breeding station / wildlife rescue center:**		**Number of staff**
Total number of staff working in tiger enclosure areas		....
If the duties of the staff are categorized by the species / group of tigers, please specify:		
• Species / Group		....
• Species / Group		....
• Species / Group		....
• Species / Group		....
• Species / Group		....
**Animal Caretaker / Keeper** (Directly responsible for tigers: feeding, cleaning enclosures, assisting with transfers between enclosures or stations)	Number of dogs kept at personal residences	....
	Number of cats kept at personal residences	....
**Veterinarian Responsible for Tigers**	Number of dogs kept at personal residences	....
	Number of cats kept at personal residences	....
**Visitors Who Access the Tiger Enclosure Area**	Number of dogs kept at personal residences	....
	Number of cats kept at personal residences	....
**Number of Staff in Other Roles (Unrelated to Animal Care)**	Number of dogs kept at personal residences	....

1.3.1. Duties of the staff at the wildlife breeding station / wildlife rescue center:		Number of staff
	Number of cats kept at personal residences	....

1.3.2. Do animal caretakers/veterinarians responsible for tigers wash their hands and change clothes before working in the enclosure?

☐ Do not wash hands or change clothes before working in the enclosures

☐ Wash hands and change clothes before working in the enclosures

If working 10 times, hands are washed approximately .... times

If working 10 times, clothes are changed approximately .... times

1.3.3. After working with tigers or in the enclosures, how often do caretakers / veterinarians wash their hands?

In 10 work times, hands are washed approximately .... times

1.3.4. Do visitors or other staff not involved with animal care wash their hands before entering the animal enclosures?

☐ Do not wash hands before entering the animal enclosure area

☐ Wash hands before entering the animal enclosure area

If entering 10 times, hands are washed approximately .... times.

1.4. Information of wild animal transport vehicles

1.4.1. Number of times wild animal transport vehicles are used for moving animals between wildlife breeding stations / wildlife rescue centers: approximately .... times/month

1.4.2. During canine distemper outbreaks, were wild animal transport vehicles used between stations?

☐ No usage

☐ Used; approximately .... times/month

1.4.3. Are wild animal transport vehicles parked in designated parking areas, and are they cleaned?

☐ Not parked in designated areas

Parked in other areas for approximately .... days/month

○ Not cleaned, and no disinfectant used after transporting animals

○ Cleaned but no disinfectant used after transporting animals

Approximately .... times/month

○ Cleaned and disinfectant used after transporting animals

Approximately .... times/month

☐ Parked in a designated parking area for approximately .... days/month

○ Not cleaned, and no disinfectant used after transporting animals

○ Cleaned but no disinfectant used after transporting animals

Approximately .... times/month

○ Cleaned and disinfectant used after transporting animals

Approximately .... times/month

1.4.4. Are there other locations where staff park wild animal transport vehicles?

☐ No

☐ Yes, parked at:

○ Private residence ○ Animal farm (specify animal species: ....

○ Roadside ○ Along the street

○ Gas station ○ Other locations (please specify: ....

Section 2: Risk of contact with canine distemper virus within the station, including the quarantine area and housing area

2.1. If tigers are brought into the wildlife breeding station / wildlife rescue center (from confiscations, donations, or transports from other stations):

2.1.1. In one year, tigers are brought into the station approximately .... times.

2.1.2. Is there a quarantine process for health observation / management before introducing tigers into the housing area?

☐ No quarantine, but observation / health management is conducted directly in the tiger housing area, which is adjacent to enclosures housing existing animals of the station or within the same vicinity.

☐ No quarantine, but observation / health management is conducted directly in the tiger housing area, located away from enclosures housing existing animals of the station by approximately .... meters.

☐ Quarantine is conducted before introducing the tigers into the housing area. For each intake, quarantine is managed as follows:

○ Quarantine is conducted every time before introducing into the housing area.

○ Quarantine is conducted only some of the time before introducing into the housing area.

2.1.3. If tigers are brought into the station 10 times, how many times is quarantine conducted before introducing them into the housing areas? Approximately .... times

2.1.4. Duration of the quarantine period: Approximately .... days/months

2.1.5. Health management practices for tigers during the quarantine period include:

....

....

....

....

....

2.2. After tigers are brought into the station, quarantined, and found to be in good health, some later display illness or canine distemper symptoms after being transferred to the housing area. This is observed in approximately .... tigers per year.

2.3. The number of staff working in both tiger enclosures and other wild animal enclosures:

Total: .... individuals. Other wild animal enclosures staff must work with include:

....

....

....

2.4. Is there shared equipment between tiger enclosures and other wild animal enclosures?

☐ No shared equipment between tiger enclosures and other wild animal enclosures.

☐ Yes, shared equipment between tiger enclosures and other wild animal enclosures:

- ○ Animal feeding equipment
  - ○ Food scoops
  - ○ Food containers, such as ....
  - ○ Food transport carts
  - ○ Water containers, such as ....
  - ○ Others, please specify ………………………………………………..……………..

- ○ Cleaning equipment shared between enclosures
  - ○ Brooms
  - ○ Floor scrub brushes
  - ○ Water buckets
  - ○ Cage scrubbing tools, such as ....
  - ○ Others, please specify …………………………………………………………..
- ○ Restraint or animal transport equipment shared between enclosures
  - ○ Animal transport stretchers
  - ○ Ropes
  - ○ Nets
  - ○ Squeeze cages
  - ○ Towels
  - ○ Tranquilizer darts for shooting or blowing
  - ○ Others, please specify …………………………………………………………..

2.4.1. <u>On average, in approximately 1 month:</u>

2.4.1.1. Frequency of using the following equipment:

- ○ Animal feeding equipment: approximately .... times/month
- ○ Animal enclosure cleaning equipment: approximately .... times/month
- ○ Restraint or animal transport equipment: approximately .... times/month

2.4.1.2. Frequency of shared equipment use between tiger enclosures and other wild animal enclosures:

- ○ Animal feeding equipment: approximately .... times/month
- ○ Animal enclosure cleaning equipment: approximately .... times/month
- ○ Restraint or animal transport equipment: approximately .... times/month

2.5. Within approximately 1 month, were domestic dogs from outside the station observed entering the station and walking in the animal enclosure areas?

☐ No domestic dogs were observed entering the animal enclosure areas.

☐ Domestic dogs were observed entering the animal enclosure areas, as follows:

○ Domestic dogs entering the tiger enclosures: approximately .... times/month.

○ Domestic dogs entering enclosures of other wild animal species (please specify the species whose enclosures the dogs entered): .... approximately .... times/month.

2.6. Number of enclosures/pens and the distance between tiger enclosures and enclosures for other wild animals:

2.6.1. Total number of tiger enclosures: .... enclosures.

2.6.2. Animal species near tiger enclosures: ....

2.6.3. Distance between tiger enclosures and other wild animal enclosures: approximately .... meters/kilometers.

2.6.4. Are there other wild animal enclosures located less than 6 meters from the tiger enclosures?

☐ No

☐ Yes, as follows:

Species .... Number of enclosures ....

Species .... Number of enclosures ....

Species .... Number of enclosures ....

Species .... Number of enclosures ....

2.7. Draw a layout of the tiger enclosures and the enclosures of other wild animal species nearby, specifying the number and approximate distances.
