# Supplementary material for: Quantitative risk assessment and interventional recommendations for preventing canine distemper virus infection in captive tigers at selected wildlife stations in Thailand
Source: PLoS One. 2025 Apr 17;20(4):e0320657. doi: 10.1371/journal.pone.0320657 (PMC12005548; doi:10.1371/journal.pone.0320657)
Supplement: Attachment — Submitted filename: Response to Reviewers.pdf [file pone.0320657.s003.pdf]

PONE-D-24-40378

Quantitative risk assessment and interventional recommendations for preventing canine distemper virus in captive tigers at selected wildlife stations in Thailand

Response to reviewers

We appreciate your careful and thorough review of our manuscript. Your insightful comments and recommendations have greatly enhanced its clarity and quality.

In this study, our quantitative risk assessment employed a stochastic model to estimate the probability of CDV introduction to captive tigers in the DNP wildlife stations through potential pathways of infection and contamination. Based on your valuable feedback, we have revised and refined the manuscript to ensure it is more robust and better aligned with your suggestions. In addition, we have refined the manuscript's writing with help from a native English speaker by Enago to enhance clarity and readability. The certificate of language editing is attached for the revised submission.

Reviewer 1:

Line 25: add the scientific name of the tiger.

Response: We have added the scientific name of the tiger "*Panthera tigris*" in Line 25.

Line 31: the phrase 'ranging from 0.716 to 0.950 (5th to 95th percentile).' seems unnecessary in the summary, as it does not provide much information as it does not contrast with other standard errors detected.

Response: We have removed it in Line 30.

Line 34: in relation to 'interventions', perhaps it could be specified that they are interventions in an epidemiological context in zoos/captive breeding stations.

Response: We have specified the interventions in an epidemiological context that were used in captive wildlife stations in Lines 33-43 as follows:

Risk probabilities were compared among those without intervention, with routine intervention at wildlife stations, and with full intervention implementation. Implementing all interventions at the

captive wildlife stations significantly reduced the risk of CDV introduction. These interventions included control measures such as quarantining and isolating infected animals and providing treatment to reduce infectiousness. Preventive measures included screening tests for healthy individuals for early detection of asymptomatic or pre-symptomatic cases, preventing further spread or complications, CDV vaccination campaigns, and promoting hand hygiene among staff and visitors. Environmental interventions involve restricting dogs and cats from accessing tiger enclosures, disinfecting animal transport vehicles, using separate equipment for each cage, etc. Together, these interventions lowered the median risk of CDV introduction to 0.089, representing an 89.6% risk reduction.

Line 39: in relation to ‘wildlife stations’, I would add zoos and private collections.

Response: We would like to clarify why we used “wildlife stations”: In the context of Thailand, there are clear differences in the management of wildlife stations, zoos, and private collections. The DNP wildlife stations where we collected data consist of wildlife breeding centers and wildlife rescue stations, which have similar management practices. These stations emphasize maintaining natural-like environments for the animals, minimizing human interaction to reduce stress, and ensuring the animals’ welfare. Limited public access is maintained to avoid disturbance. Zoos, in contrast, focus on creating safe and enriching environments for animals while also providing educational experiences for the public. They are typically open to visitors, often with an entrance fee, and their operations are often geared towards education, recreation, and conservation awareness. Private collections, which require permits from the DNP and must comply with wildlife laws, vary greatly in their management depending on the owner’s goals. While they may serve conservation purposes, they often face challenges in providing sufficient expertise, resources, or facilities for the animals, which can impact the overall welfare of the wildlife. Management in private collections is more variable compared to wildlife stations or zoos, as it is largely determined by the individual owner’s objectives and capacity. Our study focused only on wildlife breeding centers and rescue stations, not zoos and private sectors.

Additionally, we added the following sentences that informed the different types of wildlife stations, zoo, and private sectors: “Meanwhile, captive tigers are housed in wildlife stations and zoos and by private owners [9-11]. The government has implemented strict laws to protect tigers in Thailand [12]. The Department of National Parks, Wildlife, and Plant Conservation (DNP) oversees wildlife breeding and rescue centers (referred to as wildlife stations in this study), and the Zoological Park Organization of Thailand (ZPOT) manages governmental zoos.” Please see Lines 75-80.

Line 41: the phrase ' ..... and it can be applied to protect even non-captive tiger populations.' should be removed from this summary. It is not specified what these interventions in the natural habitat should be, nor is it referred to throughout the article.

Response: We have removed this phrase, and we also made the sentences flow more smoothly in Lines 43-46 as follows:

This approach assessed CDV infection risks and adapted interventions to specific situations at wildlife stations. Consistent implementation of these measures is essential to minimize CDV spread. Wildlife stations must strictly implement these interventions as standard procedures to protect the health of captive tigers.

Line 50: how is this probability found? is this an average probability? Explain or rephrase the sentence, as it gives the impression that all routes have the same probability of being entry routes.

Response: We have rephrased the sentence in Line 54 as follows: "The results revealed that the virus could enter wildlife stations with a high probability of 85.8%, representing the combined risk probability across all entry routes."

Line 59: 'can also be contracted' would be better replaced by 'could be transmitted through'.

Response: We have changed the phrase in Lines 63-65 to "CDV is spread through the air by infected coughs or sneezes and could be transmitted through contact with bodily fluids [2]."

Line 61: with regard to the phrase 'the virus can even infect other carnivores like foxes, raccoons, bears, and large felids', add the scientific names of the species that appear for the first time throughout the manuscript. Note that this virus can be detected in more than 60 species of species belonging to the Order Carnivora, and others outside this order.

Response: We have added the scientific names of the species and rephrased the sentence in Lines 66-69. Although puppies and unvaccinated dogs are most at risk, all dogs are susceptible to the virus [2]. It can also infect other animals within the order Carnivora, including foxes (*Vulpes vulpes*), raccoons (*Procyon lotor*), bears (*Ursus* spp.), and large felids (*Panthera* spp.) and species outside this order, such as the rhesus monkey (*Macaca mulata*), collared anteater (*Tamandua tetradactyla*), and Asian elephant (*Elephas maximus*) [2].

Line 68: a reference to support this sentence is missing.

Response: We have added the references in Line 76 to support this sentence as "Meanwhile, captive tigers are housed in wildlife stations and zoos and by private owners [9-11]."

9. Wildlife Conservation Office, Department of National Park, Wildlife and Plant Conservation (DNP). National Action Plan for Tiger Conservation [Internet]. 2022 [cited 2024 Dec 18]. Available from: https://portal.dnp.go.th/DNP/FileSystem/download?uuid=5e0f2d2e-b49a-4395-92b2-a7166579f535.pdf.

10. The Zoological Park Organization, Thailand. Zoo animal population online database [Internet]. 2020 [cited 2021 Oct 21]. Available from: https://www.zoothailand-service.com/quantity/population/1.

11. Environmental Investigation Agency. Cultivating demand: The growing threat of tiger farms [Internet]. 2017 [cited 2024 Dec 18]. Available from: https://eia-international.org/report/cultivating-demand-growing-threat-tiger-farms/.

Line 69: a reference is missing to support this sentence.

Response: We have added a reference in Line 77 to support this sentence as "The government has implemented strict laws to protect tigers in Thailand [12]."

12. Wildlife Conservation Office, Department of National Park, Wildlife and Plant Conservation (DNP). Ministerial Regulation designating certain types of wild animals as protected animals, B.E. 2024 [Internet]. 2019 [cited 2024 Dec 18]. Available from: https://portal.dnp.go.th/DNP/FileSystem/download?uuid=8a059247-3011-4aaa-bbd8-39bbb9b337ef.pdf.

Line 72: it would be appropriate to update the information in Table S1, excluding stations without tigers.

Response: We have excluded the wildlife stations without tigers, as updated in Table S1.

Line 72: scientific name of 'white tigers'.

Response: White tigers from zoos are the Bengal tigers (*Panthera tigris tigris*). The white tiger has a distinctive white fur color caused by a pigment abnormality. It is often found in the Bengal tiger, whose fur is usually orange. Thus, we decided to include them (seven white tigers) in the total 45 tigers in ZPO zoos (Please see Line 81). We also made it more apparent that the

Zoological Park Organization of Thailand (ZPOT) manages governmental zoos (Please see Lines 79-80).

Line 73: Revise the sentence 'For conservation value'.

Response: We revised and rewrote these sentences to "In tropical and subtropical ecosystems, tigers serve as an umbrella and iconic species, and their conservation supports broader biodiversity by maintaining ecological balance and protecting coexisting species [13]. As apex predators, they regulate prey populations, contributing to their ecosystem health [13]." Please see Lines 84-87.

Line 74: missing reference to support this sentence.

Response: We have added and changed the reference number to 13 in Line 86 and 14 in Line 87.

13. Dhakal M and Baral H. Tiger conservation in South Asia: lessons from Teari Arc landscapes, Nepal. Paper presented at the 2nd International Conference on Tropical Biology "Ecological restoration in Southeast Asia: challenges, gains, and future directions." Bogor-Indonesia. October 12-13, 2015. 1-14.

14. Smithsonian's National Zoo & Conservation Biology Institute. Tiger: fact sheet [Internet]. [Cited 2025 Jan 21]. Available from: https://nationalzoo.si.edu/animals/tiger.

Line 76: missing reference to support this sentence.

Response: We have added a reference number 15 in Line 88 as follows:

However, wild tigers are facing an additional threat from CDV, which is associated with illness and death in large felids [15].

15. Bodgener J, Sadaula A, Thapa PJ, Shrestha BK, Gairhe KP, Subedi S, et al. Canine distemper virus in tigers (*Panthera tigris*) and leopards (*P. pardus*) in Nepal. Pathogens. 2023;12(2):203.

Line 77: Put that it is in Siberia Amur Tigers (*Pantera tigris sibirica*). There is a missing reference to support this sentence.

Response: We have specified species of tigers in Lines 88-90 and added a reference as follows:

CDV poses a potential threat to the extinction of Amur tigers (*Panthera tigris altaica*) with small populations, particularly those in Russia [14].

Lines 77-79: I think these sentences should be reworded to explain more briefly the outbreak in Siberian tigers.

Response: We have reworded these sentences in Lines 90-95 to “A significant CDV outbreak affected Amur tigers in 2004 and 2010 within the Sikhote-Alin Biosphere Reserve in Russia, leading to a dramatic population decline from 25 individuals in 2008 to only 9 in 2012. Infected tigers exhibited abnormal behaviors, such as fearlessness and neurological symptoms, which caused human-wildlife conflicts and fatalities. This outbreak highlighted the susceptibility of small tiger populations to diseases introduced from reservoirs of more abundant species, such as domestic dogs and wild carnivores [14].”

Lines 79-82: These sentences lack the proper context here. I would place them above where captive breeding centres are discussed,

Response: We have removed them to the end of the paragraph where captive wildlife stations are discussed. Please see Lines 81-83.

Line 90: On ‘eighty eight’, I would choose between presenting the numerical information in word or in number.

Response: We used the numerical information in number in Lines 102-104 as follows:

“Most of the 156 affected tigers were Siberian tigers taken from private collectors. A total of 88 tigers died; the majority of deaths were suspected to be caused by CDV infection, with 31.88% (22 out of 69 dead tested tigers) primarily attributed to CDV infection.”

Line 91: ‘suspected of CDV infection’ .... what is the prevalence?. There is a missing quotation for this sentence.

Response: We have added the suspected CDV infection prevalence among deaths in Line 104 as follows:

“A total of 88 tigers died; the majority of deaths were suspected to be caused by CDV infection, with 31.88% (22 out of 69 dead tested tigers) primarily attributed to CDV infection.”

Line 94: add ‘captives’ before ‘large felids’.

Response: We have added “captives’ large felids” and removed “species” in Line 107.

Line 97-98: This sentence ‘The risk assessment results are helpful for the management of the quarantined animals, sick animal management, and viral particles spread into the environment.’ would be relocated to line 95, before ‘We used’.

Response: We have relocated this sentence to Lines 108-110.

Line 105: put 7 instead of seven.

Response: We have put 7 instead of seven in Line 118.

Line 106: insert 2 instead of two.

Response: We have put 2 instead of two in Line 119.

Line 112: define ‘semi-structured’.

Response: We have defined ‘semi-structured’ in Lines 126-129 as follows:

Data were collected from 2016 to 2021 through interviews using semi-structured questionnaires. These questionnaires contained a mix of predefined questions with specific response options (structured parts) alongside open-ended questions that allowed respondents to provide more detailed responses.

Lines 113-115: ‘We used various sources of information, including health and husbandry records of wildlife stations, laboratory databases, published literature, public databases, relevant websites, and standard guidelines.’ has this information been used to compile the survey? or what for? The survey used should be provided as supplementary material.

Response: The mentioned sources of information in Lines 137-141 (health and husbandry records, laboratory databases, published literature, public databases, relevant websites, and standard guidelines) were not utilized to compile the survey. Instead, they were used as the input parameters and probabilities incorporated into the quantitative stochastic models employed in this study (please see Supplementary S6, Tables 2-4). Therefore, we would change the sentences as follows:

Nevertheless, information from various other sources, such as health and husbandry records of wildlife stations, laboratory databases, published literature, public databases, relevant websites, and standard guidelines, were used as input parameters and probabilities incorporated into the quantitative stochastic models employed in this study.

We also replaced the sentence "The interviews were conducted on-site and online." after defining semi-structured questionnaires in Line 129 to enhance flow and readability. We have provided the questionnaire survey that was approved by Mahidol University Central Institutional Review Board (MU-CIRB) in supplementary S9.

Line 129: 'housing' and 'Cages' (Fig 2) are used indiscriminately for the same thing. Define each word in order to be able to identify the spaces. Or use one of them.

Response: We chose to use housing areas instead of cages in Line 153 because they mean the facilities designed to provide shelter, protection, and a suitable environment for wildlife species which allow them to exhibit natural behaviors. Thus, we changed the words 'Cages' in Figs 2 and 4 to 'housing areas'.

Line 133: 'CDV can enter to the wildlife stations through'.

Response: We have added 'wildlife stations' after 'CDV can enter' in Line 159.

Line 135: add 'because not all transmission routes can infect tiger cages' after 'feed.'

Response: We have added this phrase and refined the sentences in Lines 158-161 to as follows:

"Given that not all transmission routes can infect tigers in the housing areas, CDV can enter wildlife stations through infected tigers, other wild animals, domestic dog or cat reservoirs, human contamination (hands, bodies, and feet), animal-transported vehicles, equipment, and tigers' food."

Line 136: add 'Following this guide,' before 'Tigers....'

Response: We have added 'Following this guideline,' before 'tigers…' in Line 163.

Line 139: This information is confusing: 'The presence of domestic dogs and cats around wildlife stations and their access to tigers or other wild animals' cages were also considered in the entry pathway.', as in Fig 2 this pathway does not appear to be able to enter cages.

Response: We would like to explain more that the entry pathway of domestic dogs and cats cannot access tigers or other wild animals' cages directly, but in some wildlife stations, they can access the housing areas or in front of tigers or wild animals' cages. Therefore, we are concerned about this possibility of an entry pathway because CDV aerosol transmission between animals can happen in proximity within 6 meters (20 feet). We then have added a sentence to clarify this point in Lines 166-168 as follows: "The presence of domestic dogs and cats near wildlife stations and their ability to access housing areas of tigers or other wild animals were also considered in the entry pathway because CDV aerosol transmission between animals can occur."

Line 144: in relation to 'tiger food' is not considered as a netted pathway in Fig. 2.

Response: Yes, tiger food was not considered in a physical pathway (Fig. 2) because tiger food doesn't have CDV by itself. However, tiger food can be contaminated with CDV through human hands. This type of contamination has been included in the human hand pathway, and thus, tiger food was excluded from the pathway.

Line 146: in relation to 'food tongs' is not considered as a netted pathway in Fig. 2.

Response: Similarly, food tongs were excluded in a physical pathway (Fig 2) because they do not carry CDV alone and are already part of the wildlife stations' equipment, rather than being an external item introduced from outside. However, food tongs can become contaminated with CDV through human hands' visitors, and this contamination is addressed in the human hand pathway.

We have changed words in Line 172 for more clearness to 'by touching food tongs with their hands'.

Line 148: No comment on operators authorised to enter cages.

Response: We have clarified operators are authorized to enter cages in Lines 175-178 as follows:

In addition, staff members responsible for tiger care do not enter the tiger cages directly, as tigers are wild animals with instinctive aggression. Staff members do not make physical contact with the tigers. Cleaning tasks, such as washing floors or cleaning water ponds, are generally performed only after the tigers have been moved to another cage.

Line 149: Replace 'cubs' with 'cages'. Add 'external' before 'human'.

Response: We used 'cubs' intentionally (please see Line 179) because we meant that no tiger cubs or baby tigers were presented during our survey of wildlife stations. Therefore, there was no possibility of external human body contact with tiger cubs, such as through hugging or holding them.

We have made the sentence clearer and reduced any confusion about the meaning of the word “cubs” in Lines 178-180 as follows:

“During the survey of wildlife stations, no tiger cubs were present; thus, contamination through external human body contact, such as hugging or holding them, was not possible.”

Line 156: In relation to what is stated in this paragraph, operators and outsiders are not allowed to contaminate the tigers' food?

Response: We have changed words and added sentences to clarify this point in Lines 187-190 as follows:

CDV contamination via food handlers or outsiders was also excluded, as food preparation staff members work only in designated areas with strict hygiene protocols, and outsiders never handle the feed. In instances where feeding activities occur at certain wildlife stations, tools are utilized to avoid direct contact.

Line 161: add after ‘equations’ ‘for transmission through tigers, other wild or domestic animals, fences and human hands are presented in’

Response: We have added in Lines 194-196, “The biological pathways and sub-equations for transmission through tigers, other wild or domestic animals, human hands, animal transport vehicles, and equipment are presented in S2-S5 Figs.”

Line 166: replace ‘six pathways through CDV infection or contamination from the outer to the destined wildlife’ by ‘six pathways through which CDV can infect or contaminate from the outer to the destined wildlife station’.

Response: We have replaced this phrase in Lines 200-202 with “six pathways through which CDV can infect or contaminate from the outer to the destinated wildlife stations.”

Line 171: perhaps it would be better to use ‘use the entry route to’ rather than ‘entered’.

Response: We have changed ‘entered’ to ‘use the entry pathway to’ in Line 206.

Line 173: add ‘pathways’ after ‘entry’.

Response: We have added ‘pathway’ after ‘entry in Line 207.

Line 184: perhaps in material and methods it would be interesting to homogenise the term ‘entry route’ by using either ‘entry pathway’ or ‘entry route’.

Response: We have used only the term ‘entry pathway’ in material and methods for homogeneity. (Please see Lines 218, 285, 291, 295)

Line 187: add ‘CDV infection’ before ‘risk’.

Response: We have added ‘CDV infection’ before ‘risk’ in Line 221.

Line 192: add ‘CDV infection’ before ‘risk’.

Response: We have added ‘CDV infection’ before ‘risk’ in Line 226.

Line 203: define the terms ‘efficacy’ and ‘coverage’ in relation to vaccination.

Response: We have defined the term ‘efficacy’ and ‘coverage’ (Line 237) in more detailed as follows (please see Lines 248-254):

Regarding vaccination, the term “efficacy” refers to the reduction rate in disease incidence among vaccinated individuals compared with unvaccinated individuals, calculated as follows: $VE = ((ARU-ARV)/ARU) * 100$, where ARU is the attack rate in the unvaccinated population, and ARV is the attack rate in the vaccinated population [17].

17. Orenstein WA, Bernier RH, Dondero TJ, Hinman AR, Marks JS, Bart KJ, et al. Field evaluation of vaccine efficacy. Bull World Health Organ. 1985;63(6):1055-68.

“Coverage” refers to the proportion of the target population that has been vaccinated. In this study, vaccine coverage was calculated based on questionnaire data collected from wildlife stations, detailing the number of vaccinated tigers and other wildlife species.

Supporting information (S6) includes Tables 2 and 3, which present the vaccine efficacy having the detailed reference of CDV attack rates in vaccinated and unvaccinated tigers and civets, respectively, along with reference vaccine coverage data.

Line 208: why have single-use gloves not been considered as a risk mitigation measure?

Response: In Line 241, single-use gloves are an effective measure for reducing risk; however, based on practical operations in wildlife stations, handwashing is a more feasible and suitable approach. Using gloves could increase costs and may not be appropriate for the context of wildlife stations in Thailand. Therefore, we recommend handwashing as the primary intervention to mitigate CDV-contaminated human hand risks.

Line 211: why have protocols for proper disinfection of equipment not been considered as a risk mitigation measure?

Response: In Line 244, protocols for proper disinfection of equipment are recommended to reduce the risk of CDV contamination. However, questionnaire data showed that while regular cleaning of equipment is practiced, some wildlife stations still use shared equipment between animal enclosures or species. For example, brushes used for cleaning tiger enclosures are also used for small carnivores, and transport cages used for tigers are later reused for primates. These practices highlight gaps in implementing consistent disinfection protocols across all wildlife stations. Therefore, we recommend prioritizing the primary intervention of avoiding shared equipment between animal enclosures or species.

Line 222: add ‘on entry routes I and II’.

Response: We have added ‘on entry routes I and II’ after ‘for risk reductions’ in Lines 262-263.

Lines 277-280: add all scientific names of the detailed species.

Response: We have added all scientific names of the detailed species in Lines 318-327.

Line 288: why only contaminated equipment has been considered and not infected wild animals? The results show that there are minimal differences between these two routes of entry, and furthermore, the rest of the routes are well below the first three considered.

Response: We revised the heading in Lines 329-330 to reduce confusion, changing it from “Probability of CDV entry into captive wildlife stations by animal infections and fomite

contaminations" to "Probability of CDV entry into captive wildlife stations via different pathways", and the sentences to adjust the prioritization and made it more appropriate in Lines 335-338 as follows:

The second and third highest probabilities were not much different through CDV-contaminated equipment (P_{R_e}), with a median probability of 0.379 (range, 0.196-0.619, 5th-95th percentiles), and CDV-infected wild animals (P_{R_a}), with a median probability of 0.376 (range, 0.181-0.622, 5th-95th percentiles).

Line 309: How do you explain the negative impact on the risk of transmission of animal transports?

Response: We have discussed this point in the discussion, please see Lines 563-566, as follows:

"The negative correlation between the entry of animal transport vehicles and the overall probability of CDV entry may stem from the fact that the distance over which animals are transported is inversely related to the ability of the virus to survive. However, factors such as heat, dry conditions, and disinfectants can effectively inactivate CDV [31]"

Line 322: Figure 5 is poorly defined, and it is very difficult to read the legends accompanying each graph.

Response: We have remade Figure 5 to make it clearer and easier to read the legends.

Line 328-331: in relation to vaccination, the discussion should highlight that vaccination with attenuated strains of CDV has a high risk of post-vaccination CDV in some species, and that risk mitigation is much more effective with test-based surveillance for infection in animals.

Response: We have provided further discussion on a high risk of post-vaccination CDV in some species and the test-based surveillance for infection in animals, as you suggested in Lines 518-521, as follows:

"However, vaccination with attenuated CDV strains was associated with an increased risk of post-vaccination CDV infection in untested species, highlighting the importance of thorough safety evaluations before administration [14, 34]. Implementing test-based surveillance for infection in animals is a crucial strategy to minimize these risks."

Line 338: Missing definition in Figure 6, as in Figure 5.

Response: We have added the definition in Figure 6 in Line 386, as follows:

Fig 6 illustrates probabilities of entry and risk reductions employing various patterns of risk-mitigated interventions in all six entry scenarios.

We also added a description of Figure numbers (6A and 6B) for greater clarity in Lines 387–389, along with additional details on the box plot colors, elaborating on the impact of interventions to reduce the risk of virus entry into wildlife stations. Please refer to Lines 387 and 401.

Line 342: Can you explain better how it is possible to test an animal for CDV at the beginning of the journey without waiting for the result of the test? if it is positive, it cannot travel....... Rephrase the sentence to better explain this.

Response: We have explained more for the result interpretation in Lines 392-395 as follows:

Testing at the origin is crucial. Animals that are tested positive should not be transported. Follow-up testing at the destination offers additional benefits, particularly if the animal was asymptomatic or a carrier during transport. This two-staged testing approach significantly enhanced the ability to identify and manage CDV risks effectively.

Line 350: add (Fig 6A, B) at the end of the sentence.

Response: We have added '(Fig 6A and 6B)' at the end of the sentence in Line 404.

Line 401: bibliographical references 14, 15, 16, 17, 20, 21 and 22 do not appear in the text of the Discussion. Reference 23 is incorrectly numbered, as it appears after reference 25. References 18, 19, 23 and 24 are incorrectly numbered in the text as they appear after others with higher numbering.

Response: We appreciate your looking carefully. We have reordered all references already.

Line 407: In addition, disposable gloves should be worn when handling tigers.

Response: We have added this sentence in Line 469.

Line 408: I would suggest as above that CDV-infected animals should be in second place of importance, as there is little difference with contaminated equipment, and furthermore, in the

sensitive analysis it is more likely (almost twice as likely) to be a route of entry than contaminated equipment.

Response: Thank you for your suggestion; we appreciate it and are in full agreement. Therefore, we then reordered the entry of other CDV-infected wild animals to second high-risk and CDV-contaminated equipment to third importance as follows:

Lines 470-482: The introduction of other CDV-infected wild animals exhibited the second highest risk of CDV entry to captive tigers. Sensitivity analysis revealed that this pathway is more than twice as likely to contribute to CDV entry compared with contaminated equipment. Various interventions have been implemented to mitigate this risk, including CDV testing, isolating and treating sick animals, and ensuring effective vaccination protocols. Stress during animal transportation between stations can suppress the immune system, potentially triggering latent diseases such as CDV infection [27]. Performing CDV tests before entry to wildlife stations significantly reduces this risk compared with testing after entry. However, the best practice is to perform CDV testing both before and after entry. In addition, sourcing protocols for animals from exporting stations should include ensuring the absence of distemper in terrestrial carnivores within the previous 12 months and enforcing a 1-month pre-export and post-arrival quarantine at the importing station [16]. These measures, combined with ongoing CDV testing, are effective in monitoring and minimizing disease transmission risks [28].

Lines 483-491: The entry of CDV-contaminated equipment poses the third highest risk to captive tigers. Sharing equipment, such as floor-cleaning brushes or transport cages, can lead to the spread of CDV through contaminated surfaces. Strict protocols should be followed to reduce this risk, including avoiding the sharing of equipment between tiger and wild animal enclosures. Implementing such measures has decreased the risk of CDV contamination by 97.02%. This precaution aligns with earlier CDV outbreaks in captive African wild dogs and farmed civets, where contaminated equipment or human handlers were recognized as potential indirect contact pathways of transmission [38-40]. Strict biosecurity measures are essential to mitigate this risk and safeguard the health of captive tigers.

Lines 492-502: The entry probability of CDV-infected tigers is the fourth highest risk for captive tigers and has less effect on the final probability of entry, following contaminated human hands, other CDV-infected wild animals, and contaminated equipment. This lower probability of entry and effect may be due to the relatively low prevalence of CDV in the healthy tiger populations in Thailand. If a tiger is infected with CDV at its place of origin, it must be treated until fully recovered

before transport, which is already routine practice in normal situations. Although it is considered a lower priority, the strategies for mitigating this risk aligned closely with those employed in managing the entry of other CDV-infected wild animals. These strategies include performing CDV tests before and after entry, isolating and treating infected tigers, and administering effective vaccines. However, the implementation of a combination of these interventions can further and significantly reduce this risk.

Line 414: Reference 31 is on rhesus monkeys, and 33 on civets in China, so it must be a mistake that they are referencing a case in African wild dogs. Check.

Response: Please accept our mistake. Additional references for the case of African wild dogs have been added to Reference 39. Please refer to Lines 487-490 as follows:

“This precaution aligns with earlier CDV outbreaks in farmed rhesus monkeys, captive African wild dogs, and farmed civets, where contaminated equipment or human handlers were recognized as potential indirect contact pathways of transmission [38-40].”

Line 416: I think it would be the second most important net route. If it is not, I would like a coherent justification.

Response: As your suggestion, we revised in Lines 470-482, as follows:

“The introduction of other CDV-infected wild animals exhibited the second highest risk of CDV entry to captive tigers. Sensitivity analysis revealed that this pathway is more than twice as likely to contribute to CDV entry compared with contaminated equipment. Various interventions have been implemented to mitigate this risk, including CDV testing, isolating and treating sick animals, and ensuring effective vaccination protocols. Stress during animal transportation between stations can suppress the immune system, potentially triggering latent diseases such as CDV infection [27]. Performing CDV tests before entry to wildlife stations significantly reduces this risk compared with testing after entry. However, the best practice is to perform CDV testing both before and after entry. In addition, sourcing protocols for animals from exporting stations should include ensuring the absence of distemper in terrestrial carnivores within the previous 12 months and enforcing a 1-month pre-export and post-arrival quarantine at the importing station [16]. These measures, combined with ongoing CDV testing, are effective in monitoring and minimizing disease transmission risks [28].”

Lines 428-434: this paragraph is out of context here. It would be an introductory paragraph to the discussion. Also, references 18 and 19 are misnumbered in this position.

Response: We would like to elaborate and emphasize the intervention of isolating and treating symptomatic animals, as well as providing a few details about the various clinical symptoms of CDV. Additionally, it is important to address the period during which animals may remain infectious or continue to shed the virus even after recovering from illness. This information is crucial for establishing effective quarantine period, either after the animals arrive at a new station or before they leave their original station. Proper understanding and management of these factors can significantly reduce the risk of CDV transmission during animal movement.

Therefore, we have positioned this context in Lines 501-508.

Also, references 18 and 19 have been corrected to 30-31.

Line 458: reference 39 is incorrectly numbered.

Response: We have corrected a reference from 39 to 37 in Line 528.

Line 483: reference 24 is incorrectly numbered.

Response: We have corrected a reference from 24 to 47 in Line 562.

Lines 497-498: add the scientific names of species that have not appeared in the text.

Response: We have added the scientific names of species in Lines 576-578, as follows:

Past CDV outbreaks have devastated various wildlife species, including silver-backed jackals (*Canis mesomelas*), bat-eared foxes (*Otocyon megalotis*), African wild dogs (*Lycaon pictus*), and Tanzania’s lion (*Panthera leo*) in East Africa [50].

Line 575: this reference is easy to find? if not, it should be replaced by another accessible one.

Response: This reference 13 in Line 669 can be easily accessed through the following link (https://www.cifor-icraf.org/knowledge/publication/5942/). The PDF file is available at the bottom of the page.

Line 656: I could not find reference 39 in the text.

Response: Reference 39 has been corrected to 37 and is located in Line 528 in the paragraph on vaccination, the discussion part.

Reviewer 2:

Major comments:

1. It would be beneficial to include the questionnaire used in the interviews as supplementary material. Providing access to the full questionnaire would enhance transparency and offer a clearer understanding of the data collection process and its structure.

Response: Our questionnaires were originally in Thai, but we have translated them into English and included them in the supporting information section (please see S9).

2. The rationale behind the selection of specific categories in the analysis requires further clarification. Why were these particular categories chosen, and how do they align with the risk assessment model's assumptions?

- Line 158-160: i) CDV-infected tigers, ii) CDV-infected other wild animals, iii) dog or cat reservoirs, iv) CDV-contaminated human hands, v) animal-transported vehicles' wheels or mudguards, and vi) equipment contaminated with CDV

Response: Firstly, we have reviewed the transmission routes of canine distemper viruses, which can occur through both infection and contamination. Infection takes place in susceptible species, including both carnivores and non-carnivores. Transmission route between animals is aerosol or direct contact. Contamination occurs when fomites come into contact with the virus, promoting the indirect transmission of the disease. CDV can spread through contaminated objects or surfaces (fomites), such as cages, food and water bowls, veterinary tools, and even human clothing. These items can carry the virus, particularly when exposed to respiratory droplets, urine, or feces.

After understanding the transmission routes, we proceeded to outline the physical pathways within wildlife stations and identify all possible routes through which the CDV virus could enter the station. These pathways include infected tigers, other susceptible wild species carrying the virus, dogs and cats that can enter the station, CDV outbreaks in the surrounding area, contamination

through humans, vehicles used to transport wild animals, and equipment used in wild animal enclosures. By utilizing data from the questionnaire, we were able to understand the management practices of the wildlife stations and identify the most potential entry routes for CDV, aligning with the management approaches of each station. This process led to the identification of the most plausible pathways for the virus to enter wildlife stations. This is how we selected the virus entry routes and formulated my assumptions. Subsequently, we outlined the biological pathways or scenario trees for each entry route, estimated the relevant parameters, and integrated them into the quantitative stochastic risk assessment model.

We provided the rationale behind the selection of specific categories used in the risk assessment, in a paragraph named 'Quantitative stochastic risk assessment model' after Figure 2 (please see Lines 158-196).

For the data availability, the model might also assume the availability and quality of certain types of data, such as infection rates (prevalence), historical outbreaks, or current health conditions of the animals in the station. The sources of information are in Lines 270-275 and in supporting information S6 (Tables 2-4).

These assumptions, including transmission routes, species susceptibility, environmental factors, behavioral factors, and data availability, help the model estimate risks. Still, they also shape the accuracy and applicability of the results, which directly impacts the reliability of the risk assessment. Understanding and clearly defining these assumptions is crucial in any risk analysis.

2.1. Were all wild animals CDV-infected in the study (or "susceptible to CDV" Line 276)? Were there any non-infected wild animals that came into or could have come into contact with the tigers?

Response:

In Lines 101-106 of the introduction part, we have mentioned the CDV outbreak in two captive wildlife stations in Thailand. The outbreak mostly found death in tigers, with positive cases also detected in Asiatic black bears, pig-tailed macaques, and leopard cats. These three species, however, showed no clinical symptoms of the disease. Additionally, other animal species found in wildlife stations are also susceptible to CDV infection or could potentially serve as carriers of the virus. This observation aligns with previous reports indicating that CDV can infect a wide range of species within the order Carnivora, as well as species outside this order (please see Lines 66-69).

There were non-CDV infected wild animals that could have come into contact with the tigers, such as birds and reptiles. However, these animals were kept separate from the tiger enclosures and did not have direct contact with the tigers.

2.2. Regarding pathway ii), which wild animals are specifically included in this category? I recommend listing these animals along with their scientific names for clarity.

Response: The wild animals that are specifically included in the pathway category II, are wild cats (Asiatic golden cats, *Catopuma temminckii*;, fishing cats, *Prionailurus viverrinus*; leopard cats, *Prionailurus bengalensis*; clouded leopards, *Neofelis nebulosa*; leopards, *Panthera pardus*; marbled cats, *Pardofelis marmorata*; and jungle cats, *Felis chaus*), wild dogs (Asiatic jackals, *Canis aureus*; and dholes, *Cuon alpinus*), civets (small Indian civet, *Viverricula indica*; large Indian civet, *Viverra zibetha*; large-spotted civet, *Viverra megaspila*; common palm civet, *Paradoxurus hermaphroditus*; and masked palm civet, *Paguma larvata*), binturongs (*Arctictis binturong*), bears (Asiatic black bear, *Ursus thibetanus*; Malayan sun bear, *Helarctos malayanus*), and monkeys (crab-eating macaque, *Macaca fascicularis*; pig-tailed macaque, *Macaca nemestrina*; rhesus macaque, *Macaca mulatta*; and stump-tailed macaque, *Macaca arctoides*) (please see in Lines 317-327).

2.3. What is the practical and biological difference between categories i) and ii)? Why were wild animals and the domestic dog/cat categories separated into distinct biological pathways?

Response: The reason wild animals and domestic dogs/cats were separated into distinct biological pathways is that wild animals, including those already present in the wildlife station or newly introduced, are managed within the same premises as the tigers. These animals may share common areas, such as enclosures or caretakers, and use shared equipment, which increases the likelihood of cross-contamination. On the other hand, domestic dogs and cats come from outside the station, often from surrounding areas or visitors, and do not share the same care or management practices with the tigers, making their potential for direct contact with the tigers and exposure to CDV different from that of the wild animals.

2.4. I have a similar question regarding pathways v) and vi). What is the practical and biological distinction between "v) animal-transported vehicles' wheels or mudguards" and "vi) equipment contaminated with CDV"?

Response: The practical and biological distinction between "v) animal-transported vehicles' wheels or mudguards" and "vi) contaminated equipment" lies in their direct contact with the tigers. The vehicle only reaches the exterior of the enclosures and does not come into direct contact with the tigers themselves. In contrast, equipment such as cleaning brushes or other tools can access the interior of the enclosures, allowing for direct contact with surfaces or areas that the tigers interact with. Therefore, contaminated equipment has a higher potential for direct transmission of the virus to the tigers.

Minor comments:

Introduction

Line 98: I suggest expanding the sentence as follows: „and preventing the spread of viral particles into the environment."

Response: We have expanded this sentence in Lines 108-110 as follows:

The risk assessment results help in the management of quarantined and sick animals and the prevention of the spread of viral particles into the environment.

Materials and methods

Line 105: I suggest expanding the sentence as follows: „seven station"

Response: We have expanded this sentence in Line 118 as follows:

This study focused on 7 stations located in different regions in Thailand, which have more than 2 tigers (Fig 1).

Line 112-117: I understand that the data will be presented in detail later; however, to improve transparency, the "Data Collection" section could be expanded. For instance, it would be helpful to include details such as the total number of interviews conducted and the distribution of completed interviews across different stations. This additional information would provide a clearer understanding of the methodology.

Response: We have provided more information in the "Data Collection" about details of interviews conducted and the distribution of complete interviews across different stations as follows (Please see Lines 129-136):

The plan was to interview one director or veterinarian per wildlife station and approximately three animal keepers per station, or as many as were available. Across seven wildlife stations, a total of interviews was conducted. This included interviews with directors from four stations and veterinarians from three stations using questionnaire number 1 (QN1). In addition, due to variations in the number of staff at different stations, a total of 18 interviews with keepers were conducted. These comprised 15 tiger keepers, 2 keepers of other susceptible wild animals (1 bear keeper and 1 monkey keeper), and 1 animal husbandry staff member

Line 135, 144, 146, 155: tiger food or feed?

Response: We have changed words 'feed' in these lines to 'tigers' food'.

Results

Line 276-279: I recommend providing the precise identification of species, including their scientific names, to ensure clarity and accuracy.

Response: We have provided the precise identification of species, including their scientific names in Lines 317-327.

Line 298: Were the dogs and cats also infected with CDV?

Response: Yes, dogs can be infected with CDV. However, domestic dogs are the primary reservoirs of CDV among wild animals (please see Line 544). Cats can become infected with CDV, but the virus generally causes asymptomatic symptoms (please see Lines 549-550). Therefore, we input the entry pathway from dogs and cats as they are the reservoirs of CDV.

Discussion

Line 399-401: I suggest replacing the bacterial examples with studies focused on viruses. If the bacterial references remain in the text, I recommend revising the phrase '80% of germs can get on your hands and then go on to other things' to something like: 'can be transferred to hands during contact and potentially contaminate objects or surfaces'

Response: We revised the sentences to get a better understanding as follows:

Lines 453-455: This value is consistent with the results of a study reporting that 80% of germs can be transferred to hands during contact and can potentially contaminate objects or surfaces [19].

19. SC Johnson Professional USA, Inc. Hand hygiene: best practice [Internet]. 2024 [cited 2025 Jan 4]. Available from: https://www.scjp.com/en-gb/hand-hygiene-best-practice-1.

Additionally, we added more information focusing on the effective cleansing of viruses as follows:

Lines 455-463: The use of plain or antiseptic soap effectively eliminates germs; even the use of plain soap and water alone can reduce bacteria by up to 92% [20-23]. Handwashing with soap and water or using alcohol-based hand rubs reduces the number of H1N1 virus particles to levels undetectable in cultures and approximately 100 virus copies/mL in PCR tests, achieving a reduction rate of >99.99% in virus particles on hands [24]. H1N1 and CDV are negative-sense RNA viruses that share structural similarities, including lipid envelopes and surface proteins that are essential for entering host cells. They also have similar modes of transmission, including respiratory droplets, direct contact, and fomites [24]. These similarities make them highly susceptible to disinfectants that target lipid envelopes, such as soap.

24. Grayson ML, Melvani S, Druce J, Barr IG, Ballard SA, Johnson PD, et al. Efficacy of soap and water and alcohol-based hand-rub preparations against live H1N1 influenza virus on the hands of human volunteers. Clin Infect Dis. 2009;48: 285-91.

Line 496-498: I suggest including the scientific names of the species that have not been mentioned previously in the text.

Response: We have added the scientific names of the species that have not been mentioned previously in Lines 576-578.
